# Divergence of annual and perennial species in the Brassicaceae and the contribution of cis‐acting variation at *FLC* orthologues

**DOI:** 10.1111/mec.14084

**Published:** 2017-03-22

**Authors:** C. Kiefer, E. Severing, R. Karl, S. Bergonzi, M. Koch, A. Tresch, G. Coupland

**Affiliations:** ^1^ Max Planck Institute for Plant Breeding ResearchPlant Developmental BiologyCarl‐von‐Linné Weg 1050829CologneGermany; ^2^ Department of Biodiversity and Plant SystematicsCentre for Organismal StudiesINF 34569120HeidelbergGermany; ^3^ Wageningen UR Plant BreedingWageningen University and Research CentreDroevendaalsesteeg 16708 PBWageningenThe Netherlands; ^4^ Cologne BiocenterUniversity of CologneZülpicher Str. 47b50674CologneGermany

**Keywords:** annual, *Arabis*, *FLC*, flowering time, *PEP1*, perennial

## Abstract

Variation in life history contributes to reproductive success in different environments. Divergence of annual and perennial angiosperm species is an extreme example that has occurred frequently. Perennials survive for several years and restrict the duration of reproduction by cycling between vegetative growth and flowering, whereas annuals live for 1 year and flower once. We used the tribe Arabideae (Brassicaceae) to study the divergence of seasonal flowering behaviour among annual and perennial species. In perennial Brassicaceae, orthologues of *FLOWERING LOCUS C* (*FLC*), a floral inhibitor in *Arabidopsis thaliana*, are repressed by winter cold and reactivated in spring conferring seasonal flowering patterns, whereas in annuals, they are stably repressed by cold. We isolated *FLC* orthologues from three annual and two perennial *Arabis* species and found that the duplicated structure of the *A. alpina* locus is not required for perenniality. The expression patterns of the genes differed between annuals and perennials, as observed among *Arabidopsis* species, suggesting a broad relevance of these patterns within the Brassicaceae. Also analysis of plants derived from an interspecies cross of *A. alpina* and annual *A. montbretiana* demonstrated that *cis*‐regulatory changes in *FLC* orthologues contribute to their different transcriptional patterns. Sequence comparisons of *FLC* orthologues from annuals and perennials in the tribes Arabideae and Camelineae identified two regulatory regions in the first intron whose sequence variation correlates with divergence of the annual and perennial expression patterns. Thus, we propose that related *cis*‐acting changes in *FLC* orthologues occur independently in different tribes of the Brassicaceae during life history evolution.

## Abstract 2

see also the Perspective by Friedman

## Introduction

Life history varies greatly among higher organisms and can diverge rapidly during evolution (Partridge & Harvey 1988). Two major life history strategies have been described, which are represented by semelparous organisms that reproduce only once in their lifetime and iteroparous organisms that undergo several reproductive periods (Silvertown & Charlesworth 2001). In plants, these life strategies are often referred to as monocarpic (semelparous) or polycarpic (iteroparous) and are defined by the rate of reproduction and probability of survival at each stage of the life cycle. Most monocarpic plants are annual, completing their life cycle within 1 year, while polycarpic plants are usually perennial, surviving for many years and entering the reproductive phase multiple times. Evolutionary transitions between perenniality and annuality have occurred often among higher plants. In most cases, annuality seems to be derived from perenniality (Hu *et al*. 2003; Grillo *et al*. 2009), but the reverse has also been described (Tank & Olmstead 2008).

Variation in life history is associated with adaptation to different environments. Annuals are usually found in open, dry habitats prone to seasonal drought but where seedlings have high survival rates if germination is appropriately timed. By contrast, perennials are more commonly found in mesic habitats where seedling mortality is high (Stebbins 1950; Silvertown & Charlesworth 2001). For example, perennial populations of *Mimulus guttatus* (Scrophulariaceae) have access to moisture throughout the year, while annual populations grow in areas that are arid during the summer months, and reciprocal transplant experiments indicate that each type is adapted to its respective environment (Hall & Willis 2006).

Theoretical models support the proposal that life history evolves to optimize the number of offspring produced while minimizing the costs of reproduction (Stearns 2000; Friedman & Rubin 2015). A deeper understanding of life history evolution and more specifically of the transition from perennial to annual requires knowledge of the developmental features that change during the transition and the associated underlying genetics. A critical feature is the initiation of reproduction through the induction of flowering. Perennials cycle multiple times through vegetative and reproductive states, which requires repeated decisions on the fate of individual meristems. These decisions involve, at least in part, flowering‐time genes whose roles are extended in perennials compared to annuals (Friedman & Rubin 2015). These mechanisms of flowering control have been studied in several perennial species and compared to that of the well‐established annual model species *Arabidopsis thaliana* (Albani & Coupland 2010; Wang *et al*. 2011; Andres & Coupland 2012; Iwata *et al*. 2012; Koskela *et al*. 2012; Bergonzi *et al*. 2013; Zhou *et al*. 2013).

*PERPETUAL FLOWERING 1* (*PEP1*), the *A. alpina* orthologue of *A. thaliana FLOWERING LOCUS C* (*FLC*), has critical roles in the perennial life cycle. FLC is a MADS box transcription factor that delays flowering before vernalization (Michaels & Amasino 1999; Sheldon *et al*. 1999). *PEP1*, in addition to preventing flowering before vernalization, restricts flowering to a short episode after vernalization by causing reversion to vegetative growth after flowering (Wang *et al*. 2009; Castaings *et al*. 2014). This additional function of *PEP1* is conferred by a distinct pattern of transcriptional regulation compared to *FLC*. The latter is stably repressed by vernalization allowing *A. thaliana* to flower continuously after vernalization. In contrast, *PEP1* is only temporarily repressed by vernalization and rises in expression again when plants are returned to warm. This cyclical repression of flowering is proposed to contribute to the perennial life cycle by preserving meristems for vegetative growth upon the return to warm temperatures (Wang *et al*. 2009). Similar cycling patterns of expression of *FLC* orthologues were described in other perennial Brassicaceae species (Aikawa *et al*. 2010; Kemi *et al*. 2013). Repression of expression of both *FLC* and *PEP1* during vernalization correlates with accumulation of trimethylation on lysine 27 of histone 3 (H3K27me3) starting from a region referred to as the nucleation region (Bastow *et al*. 2004; Wang *et al*. 2009; Angel *et al*. 2011; Yang *et al*. 2014).

At *FLC,* this modification remains after vernalization stably repressing gene expression, whereas in *A. alpina,* it disappears after the return of plants to warm, correlating with reactivation of *PEP1* (Bastow *et al*. 2004; Sung & Amasino 2004; Finnegan & Dennis 2007; Wang *et al*. 2009). Repression of *FLC* is also regulated by noncoding RNAs expressed *in cis* during vernalization (Swiezewski *et al*. 2009; Heo & Sung 2011; Csorba *et al*. 2014). COOLAIR is an antisense RNA that is conserved at *PEP1* (Swiezewski *et al*. 2009; Castaings *et al*. 2014; Csorba *et al*. 2014), whereas COLDAIR is a sense RNA expressed from the first intron and associated with a sequence important for repression of *FLC* transcription during vernalization called the vernalization response element (VRE; Sung *et al*. 2006; Heo & Sung 2011). *PEP1* has a more complex structure than *FLC*, so that the first exon is duplicated in tandem along with the proximal promoter and part of the first intron giving rise to two overlapping transcripts (Albani *et al*. 2012). In perennial *Arabidopsis lyrata,* the *FLC* locus is tandemly duplicated, while in perennial *Arabidopsis arenosa,* it is partially triplicated (Nah & Chen 2010). This indicates that rearrangements at *FLC* orthologues might contribute to the rapid evolution of life history in the Brassicaceae (Alonso‐Blanco & Méndez‐Vigo 2014).

Understanding trait evolution requires systematic analysis of closely related species within a well‐developed phylogenetic framework. This approach has proven effective in, for example, identifying the mechanisms and direction of evolution of wing pigmentation patterns in the *Drosophila* genus (Prud'homme *et al*. 2006; Arnoult *et al*. 2013). The Brassicaceae provide similar advantages for studying the evolution of annual and perennial life history. Several genera in this family such as *Arabidopsis*,* Brassica*,* Draba* and *Arabis* contain both annual and perennial species. Notably, annual life history and perennial life history have diverged independently several times in the *Arabis* and *Draba* genera of the tribe Arabideae, which is the largest tribe of the Brassicaceae and comprises ~500 species (Couvreur *et al*. 2010; Al‐Shehbaz *et al*. 2011; Karl *et al*. 2012). Typically, perennial species‐rich groups are sister to annual species‐poor groups, and it has been suggested that life history changes are a major driving factor for diversification in the Arabideae (Karl & Koch 2013).

Comparative analyses of annual and perennial species within the Arabideae provide an opportunity to study the repeated divergence of these traits within relatively short evolutionary timescales. *Arabis alpina* has been used as a model genetic system to study perennial traits, its genome has been sequenced (Willing *et al*. 2015), and it exhibits a broad arctic–alpine distribution range originating from migrations out of Asia Minor (Koch *et al*. 2006; Ansell *et al*. 2011; Karl *et al*. 2012).

Here, we perform an intensive phylogenetic analysis of the main branches of the Arabideae represented by 16 taxa and relate this to the life history of individual species. These experiments demonstrate that *A. montbretiana* is an annual sister of perennial *A. alpina* and that their sister is the perennial *Arabis nordmanniana*. Consistent with their respective life histories, the *FLC* orthologue of *A. montbretiana* is stably repressed by vernalization in contrast to *PEP1* of *A. alpina*. The close phylogenetic relationship of these two species allowed us to perform interspecies crosses and to demonstrate that *cis*‐acting variation contributes to the different patterns of expression of the *FLC* orthologues in these species. Detailed sequence comparisons of *FLC* orthologues isolated from species across the Arabideae and Camelineae tribes identified regions in the first intron that we propose contribute to the divergence of gene regulation between annual and perennial Brassicaceae species.

## Materials and methods

### Plant material

Plant material was obtained from Birol Mutlu, Turkey (*A. montbretiana*, BM7968, voucher HEID809801, RK043 in Karl & Koch 2013; Jipei Yue, Kunming, China (*Scapiarabis setosifolia*, RK025 in Karl & Koch 2013), Maarten Koornneef, MPIPZ Cologne, Germany (*Arabis purpurea*), Santiago Martin Bravo (*Arabis nova* subsp. *iberica*) and the Botanical Garden Heidelberg (*Arabis alpina*, RK001 and A‐Kili; *Arabis aucheri* RK041; *Arabis auriculata*,* Arabis collina* B‐2006‐0564 and JR045, *Arabis hirsuta* JR38, *Arabis nordmanniana* RK219, *Arabis purpurea* RK004, *Arabis verna* RK212, *Aubrieta canescens* subsp. *macrostyla* RK214, *Draba aizoides* Dra 002, *Draba hispanica* Dra 0026, *Pseudoturritis turrita* RK237 (all numbers referring to Karl & Koch 2013; *Draba nemorosa* B‐2002‐0157). As comparison, the *A. alpina* acc. Pajares (Wang *et al*. 2009) was used. Accession details are given in Table S1 (Supporting information).

### Generation of an *Arabis montbretiana* × *Arabis alpina* F1 hybrid

To investigate the flowering behaviour of hybrids between annuals and perennials, *A. montbretiana* flowers were emasculated and cross‐pollinated with *A. alpina* acc. Pajares pollen. One viable plant was obtained through embryo rescue (adapted from Sauer & Friml 2008). Details are given in Supplementary Methods (Supporting information). For verification of the hybrid identity of the F1 plant, one of the phylogenetic markers used for the phylogenetic analysis (*ITS*) was amplified as described above and Sanger‐sequenced using the forward and reverse PCR primers. Sequences were analysed for double signals in positions being polymorphic in both parents.

### Growth and vernalization conditions

Plants were cultivated on soil [Balster, Sinntal‐Altengronau, Germany (Type Mini‐Tray)] under a photoperiod of 16 h and a temperature of 20 °C during the day (minimum 18 °C at night) in three different glasshouse (GH1, GH2, GH3, Supplementary Methods, Supporting information). Plants were watered daily and fertilized regularly with Wuxal Super 8‐8‐6 and/or Wuxal Top K 21%, or a comparable fertilizer mix (Supplementary Methods, Supporting information).

For vernalization, two vernalization rooms were used (Viessmann, Allendorf, Germany, or Fitotron by Weiss Technik, UK) which all had a constant temperature of 4–5 °C and were run with short photoperiods of 8 h (light sources in Supplementary Methods V1, V2, Supporting information). During vernalization, plants were not fertilized and watered only when necessary.

### Cultivation of plants used for expression analyses

Seeds of *A. montbretiana*,* A. alpina* acc. Pajares, *A. auriculata* and *A. nordmanniana* were either stratified on wet filter paper or sown directly on soil and stratified for 3–4 days at 4 °C in darkness. Four to six plants were grown in the glasshouse (GH1, Supplementary Methods, Supporting information) under the conditions described above. Vernalization treatment was performed 3 (annuals) or 6 weeks (perennials) after seed germination for 12 weeks (Viessmann, Allendorf, Germany, V1, Supplementary Methods, Supporting information). The ages at which plants were exposed to vernalization were selected because *A. alpina* acc. Pajares has a juvenile phase of 5 weeks during which flowering cannot be induced. The annuals on the other hand were found to have a shorter juvenile phase (for details on *A. montbretiana* and *Arabis auriculata*, see the Results and Supplementary Results sections, Supporting information). At the end of the vernalization period, plants were transferred back to the glasshouse conditions described above. Cuttings from the *A. montbretiana* **×** *A. alpina* hybrid were grown in the same way. For the experiment on the lines containing the introgressed *AmFLC* region, seeds were stratified for 4 days at 4 °C in darkness and then germinated on filter paper. Seedlings were transferred to soil and then cultivated under glasshouse conditions for 8 weeks (GH3, V2, Supplementary Methods, Supporting information). Plants were vernalized for 12 weeks at 5 °C. After vernalization, plants were returned to glasshouse conditions. Vernalization time‐courses were run in two replicates.

### Validation of life history

All accessions except *Scapiarabis setosifolia*,* Pseuditurritis turrita*,* Draba aizoides*,* Arabis purpurea* RK004 and *Arabis alpina* RK001 were cultivated under glasshouse conditions (GH1, Supplementary Methods, Supporting information). After flowering, it was determined whether the plant underwent complete senescence (annual) or continued to grow vegetatively (perennial). These results were reproduced in the other two glasshouses used in this study.

### Assessment of vernalization response

After germination, plants were grown either constantly under glasshouse conditions (GH1, GH2, Supplementary Methods, Supporting information) or exposed to vernalization 3 or 6 weeks after germination for 12 weeks (V1, Supplementary Methods, Supporting information). Flowering was scored as total leaf number (TLN) on the main shoot under control conditions or after vernalization. For *A. montbretiana* and *A. auriculata*, also the age at which the plant responds to vernalization was determined experimentally. For this experiment, *A. montbretiana* was grown under long‐day glasshouse conditions. Plants were shifted into vernalization at an age ranging from 1 to 6 weeks or left in long‐day conditions without vernalization. After 12 weeks, plants were returned to warm temperatures and long‐day conditions and TLN at flowering was scored. In a second experiment, *A. montbretiana* plants were grown in a plant growth chamber under 16 h long days (Grow Bank, CLF Climatic, Wertingen, Germany) at constant 22 °C and a light intensity of 150 μE. They were then vernalized for 12 weeks at 1–6 weeks after germination, as described above. A similar experiment was run for *Arabis auriculata*, which was shifted at two different ages (after 3 and 6 weeks of growth in GH1), and a control was maintained under long‐day glasshouse (GH1) conditions and not exposed to vernalization.

### DNA extraction

For PCR and sequencing, genomic DNA was extracted with the DNeasy Plant Mini or Maxi Kit (Qiagen, Germany) following the manufacturer's protocol. DNA concentration and quality were checked by measuring OD 260 and OD 260/280 using a Nanodrop photometer (Thermo Fisher Scientific, USA). If the DNA concentration was too low, DNA was concentrated by evaporation using a Concentrator plus apparatus (Eppendorf, Hamburg, Germany). The quality of the DNA used for whole‐genome sequencing of *A. montbretiana* and *A. nordmanniana* was also checked on an agarose gel [0.5% agarose in TAE (40 mm Tris, 20 mm acetate, 1 mm EDTA)].

### Amplification and sequencing of phylogenetic marker sequences, alignment and phylogenetic reconstruction

Four nuclear and two plastidic marker sequences were amplified and sequenced for use in phylogenetic reconstruction (for material, see Table S1, Supporting information). All of the selected phylogenetic marker sequences were successfully used for phylogenetic reconstruction in previous studies (White *et al*. 1990; Taberlet *et al*. 1991; Mummenhoff *et al*. 1997; Koch *et al*. 2000; Dobeš *et al*. 2004; Duarte *et al*. 2010). As orthologues of *At2g13360* had only been used in a small study to demonstrate its value as a phylogenetic marker, its orthologue was identified in the genome assembly of *A. alpina* acc. Pajares (Willing *et al*. 2015). Synteny of the respective genomic regions around *At2g13360* of *A. thaliana* and its orthologue in *A. alpina* was analysed using the program gata (Nix & Eisen 2005). When synteny was confirmed, primers were designed using the program primer3 (Rozen & Skaletsky 2000) amplifying exons 1 to 3 including all introns (product ~1 kb, primers in Table S2, Supporting information). Primer sequences, including the ones for the amplification of *ITS* (White *et al*. 1990; Mummenhoff *et al*. 1997), trnL (Taberlet *et al*. 1991), the trnL‐F IGS (Taberlet *et al*. 1991; Dobeš *et al*. 2004), *CHS* (Koch *et al*. 2000) and *ADH* (Koch *et al*. 2000), are given in Table S2 (Supporting information). Phylogenetic marker sequences were amplified by PCR from each species (see supplement for method, Supporting information). PCR products were separated on agarose gels which confirmed that only one PCR product was present, purified and sequenced directly or after cloning (see supplement for method, Supporting information) by Sanger sequencing. Electropherograms of the obtained sequences were controlled for quality using the program SeqMan (DNASTAR, Lasergene).

Both chloroplast markers and if necessary both parts of the *ADH* fragment were combined prior to aligning and were analysed as a unit. Sequences of the six utilized phylogenetic markers (ITS, *trn*L, *trn*L‐F, *CHS, ADH* and orthologue of *At2g13360*) were aligned with clustalx (Thompson *et al*. 1997) and subsequently adjusted by hand. For the markers *CHS*,* ADH* and orthologue of *At2g13360*, only the exons of the sequences were employed for the analyses (alignment in Table S3, Supporting information). After model testing (Table S4, Supporting information), Bayesian MCMC analyses (Yang & Rannala 1997) were performed with the mpi (message parsing interface) version of mrbayes version 3.1.2 (Ronquist & Huelsenbeck 2003). Additionally, a maximum parsimony analysis was run using both co‐orthologues of the nuclear phylogenetic markers of *A. nordmanniana* (alignment in Table S5 , Supporting information). Details are given in Supplementary Methods (Supporting information).

### Contig assembly of *A. montbretiana* and *A. nordmanniana* and identification of *FLC* orthologues in five Arabideae taxa

For *A. montbretiana* and *A. nordmanniana*, Illumina libraries with an insert size of 400 bp were constructed, sequenced by Illumina sequencing (120 bp, paired ends), and contig assemblies based on 185 (*A. montbretiana*) and 415 million reads (*A. nordmanniana*) were generated using the clc genomics workbench version 4.7 or version 8.5, respectively, using standard settings. The contig assemblies were indexed as blast libraries and searched using *PEP1* (Wang *et al*. 2009) as query. In the case of *A. nordmanniana*, the blast search resulted in several blast hits to *FLC* or flanking genes. When the contigs were assembled, two co‐orthologues of *FLC* were generated. The assembly of the individual contigs was confirmed by PCR and Sanger sequencing of the PCR fragments. The genome size of *A. nordmanniana* was estimated by kmer‐depth. The *A. nordmanniana* assembly was annotated using the maker pipeline (Cantarel *et al*. 2008). orthomcl (Li *et al*. 2003) was used to define orthologous groups. Details of the settings and methods used for the *A. nordmanniana* assembly and annotation are described in Supplementary Methods and Results (Supporting information).

Regions containing the *FLC* orthologues from *A. auriculata*,* A. purpurea* and *A. nova* subsp. *iberica* were each amplified by PCR (primers in Table S2, Supporting information) as three overlapping fragments. The first fragment spanned a region from the adjacent upstream gene (orthologue of *At5g10150*) and ended at the beginning of the first intron (amplification of intergenic region between orthologue of *At5g10150* and *FLC* orthologue), and the second covered the region containing a part of exon 1, intron 1 and a part of exon 2 (amplification exon 1 to exon 2). The third PCR fragment contained a part of exon 2 and ended downstream of exon 7 (amplification exon 2 to exon 7). Various combinations of primers were used for Sanger sequencing the PCR products before or after cloning into the pGEM‐T vector (see Supplementary Methods, Supporting information). Primers were designed manually or using the program primer3 (Rozen & Skaletsky 2000). To follow the structural evolution of the FLC orthologues, sequences were aligned by mVISTA (Mayor *et al*. 2000; Frazer *et al*. 2004) and compared to *A. thaliana FLC*. Alignments were adjusted manually. Exons (by using cDNA data) and sequences with homology to known regulatory elements from *FLC* were identified and annotated. The annotations were used as a basis for generating locus schemes that were used for following the structural evolution of *FLC* orthologues. gata (Nix & Eisen 2005), mvista (Mayor *et al*. 2000; Frazer *et al*. 2004), yass (Noe & Kucherov 2005) and clustalw (Larkin *et al*. 2007; Goujon *et al*. 2010; McWilliam *et al*. 2013) were used for different aspects of sequence analysis (details in Supplementary Methods, Supporting information).

### Expression analysis of *PEP1*,* AmFLC*,* AauFLC* and *AnFLC‐A* and *AnFLC‐B*

Leaf samples of *A. alpina* acc. Pajares, *A. auriculata*,* A. montbretiana* and *A. nordmanniana* were taken between ZT 4 and ZT 7 before (3 or 6 weeks in long‐day conditions depending on the experiment), at the end (after 12 weeks) and after vernalization (2, 3, 4 weeks in long day after vernalization depending on the experiment) in two biological replicates grown at different times (only one replicate was used for *A. alpina* because this confirmed data published by Wang *et al*. 2009). One young leaf from the main shoot was harvested at each time point, and samples were pooled from four to six plants, except for *A. nordmanniana* where one individual was monitored in each replicate.

Total RNA was extracted using the rneasy mini kit (Qiagen, Germany). The obtained RNA was treated with DNase (Ambion, Thermo Fisher Scientific, USA) and then transcribed into cDNA using the Superscript II Reverse Transcription Kit (Invitrogen, Thermo Fisher Scientific, USA). All kits were used according to the manufacturer's protocols.

All qPCRs were performed in a volume of 20 or 15 μL containing 2 μL cDNA, 2/1.5 μL 10× PCR buffer (Invitrogen, Thermo Fisher Scientific, USA), 1 μL Eva green (Biotium, USA), 2.5 mm MgCl_2_, 0.5 mm dNTPs, and 0.2 μL My Budget Taq polymerase (Bio‐Budget Technologies GmbH, Krefeld, Germany) or Taq DNA polymerase (Invitrogen, Thermo Fisher Scientific, USA). PCRs were run on a Bio‐Rad (California USA) Realtime PCR cycler or Roche iCycler using the primers published previously for *A. alpina* (Wang *et al*. 2009 and Bergonzi *et al*. 2013; primer names *PEP1* (qPCR) and *PP2A* (qPCR) as reference gene; Table S2, Supporting information) in triplicates or quadruplicates. If necessary, primer sequences were adjusted to point mutations present in the priming sites in the different species (cDNA sequences of *FLC* orthologues in Fig. S1 (Supporting information); *A. nordmanniana* primers *AnFLC‐A* (qPCR), *AnFLC‐B* (qPCR) and *AnRAN3* (qPCR) as reference gene; *A. montbretiana* primers *AmFLC* (qPCR) and *PP2A* (qPCR) as reference gene; *A. auriculata* primers *AauFLC* (qPCR), *AauPP2A* (qPCR) as reference gene; primer sequences in Table S2, Supporting information). As there is no mutation in the priming site for *PP2A* between *A. alpina* and *A. montbretiana*, both *AmPP2A* and *AaPP2A* were measured simultaneously. Data were analysed using the program excel 2013 (Microsoft Office) by the delta Ct method. The standard deviation was calculated separately for triplicates or quadruplicates of reference and target gene and then combined by error propagation. For easier display in the figures, the expression at 0 weeks of vernalization was set to 100% and expression at the other time point was recalculated relative to this time point. Efficiency of the PCR was determined by standard curves.

### Genotyping of the *A. montbretiana* **×** *A. alpina* F1 and resulting introgression lines

Based on the genomic information available (Willing *et al*. 2015; Kiefer *et al*. in preparation), length polymorphism markers distributed across the complete genomes were designed to distinguish *A. alpina* and *A. montbretiana*. To confirm the cross of *A. montbretiana* **×** *A. alpina* and initial genotyping of the subsequent generations, 40 length polymorphism markers were used (Table S8, Supporting information). For genotyping the introgression lines further, 84 markers were employed (Table S8, Supporting information). Length polymorphism markers were amplified in multiplexing PCRs containing two to three primer pairs (2 μL GoTaq buffer (Promega, Wisconsin, USA), 2 μL template, 1 μL MgCl_2_ (25 mm), 0.4 μL dNTPs (10 mm), 0.4 μL of each primer pair (each primer 10 μm), 0.05 μL GoTaq (Promega, Wisconsin, USA), 3.35 μL water), separated on 3% agarose gels (TAE) and scored as heterozygous or homozygous for either parent. *Arabis alpina* and *A. montbretiana* were used as control. All primers were designed using the program primer3 (Rozen & Skaletsky 2000). For the introgression lines, genotypes were plotted using the program flapjack 1.016.03.04 (Milne *et al*. 2010).

### Estimates of evolutionary divergence of the first intron of *FLC* orthologues

All calculations on (average) evolutionary divergence of sequences of the first intron of the *FLC* orthologue from *A. alpina* acc Pajares and *A. montbretiana* (number of base substitutions per site) were performed in mega5 (Tamura *et al*. 2011) using the maximum composite likelihood model (Tamura *et al*. 2004). In the analysis referring to the alignment used for Fig. 4, the alignment was split at position 10 332 into the ‘5’ part’ (10 332 alignment positions; 2755 positions without missing data and gaps) and the ‘3’ part’ (10 333 to end >4167 alignment positions; 4018 positions without gaps).

### Analysis of SNP and indel distribution between *PEP1* and *AmFLC*

To define SNP evolutionary patterns among *PEP1* and *AmFLC*,* AlFLC1* and *FLC*,* AmFLC* and *AuFLC* as well as *PEP1* and *AnFLC‐A* SNPs were scored for each alignment position in a Microsoft Excel table. Positions including gaps, missing data, ambiguous data or where more than two possibilities were present were excluded from the analysis. SNPs were then analysed by a sliding window approach. The window size was 100 bp, and the window was shifted by 20 bp or 1 bp. We used a binomial test to examine the over‐ or underrepresentation of SNPs within each window, comparing the actual number of observed SNPs with the distribution of SNPs, given an overall SNP occurrence rate. *P*‐values were converted into expected false discovery rates (FDR) to account for multiple testing. An FDR of <20% was considered significant. Insertions were coded as one mutation and also scored by a sliding window approach using the same window size and shift as above.

## Results

### Phylogenetic reconstruction based on annual and perennial representatives of the Arabideae

**Figure 1:**
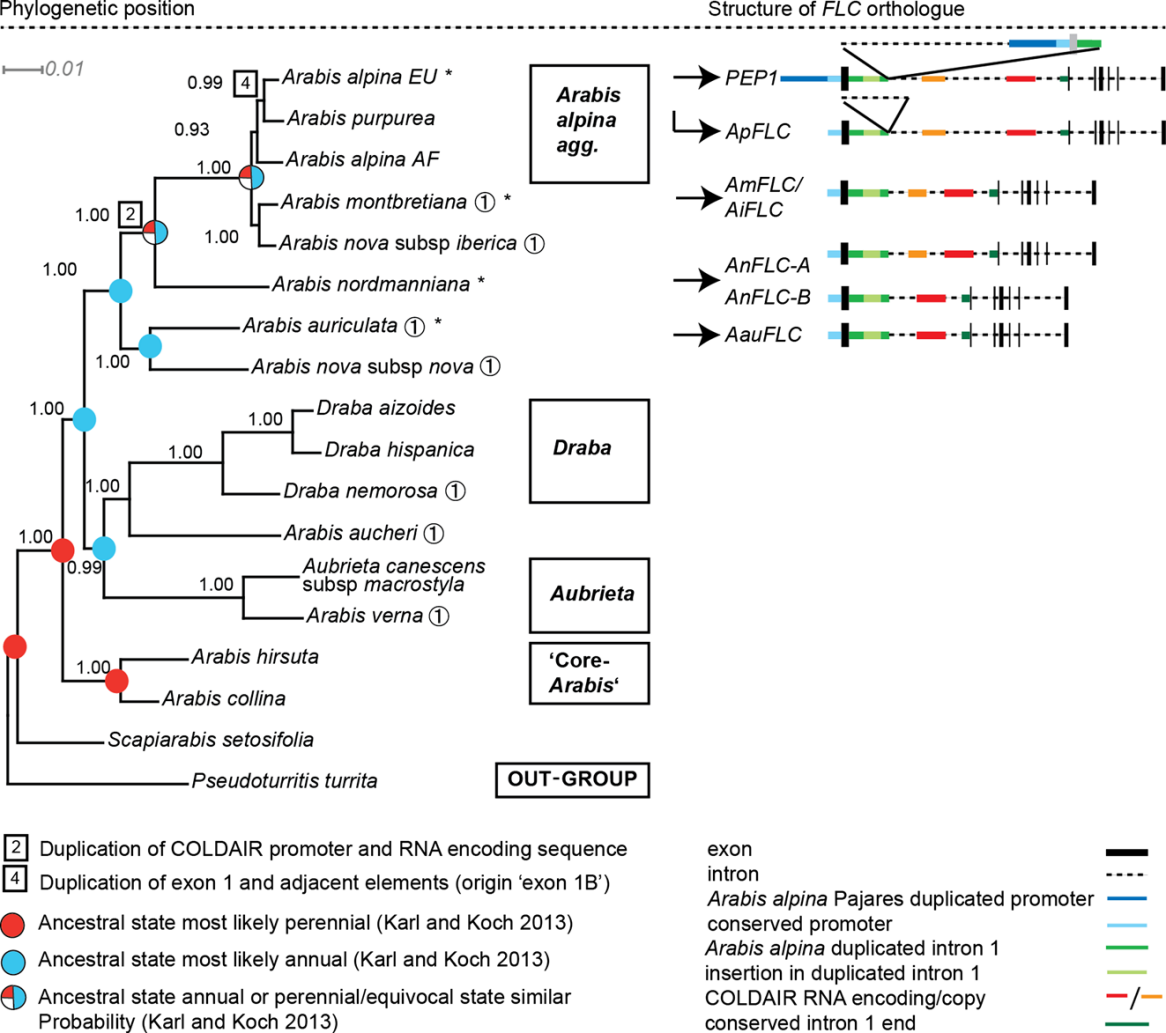
Left: Bayesian phylogenetic analysis of 17 representative annual and perennial species of the Arabideae based on a concatenated alignment of *ITS*,* trnL*,* trnLF*,*CHS*,*ADH* and *At2g13360*. Names of groups and/or larger genera of which only representatives were used in the phylogenetic reconstruction are given in boxes. Annuals are indicated by an encircled 1, posterior probability values are given at all nodes. *Arabis alpina* and *Arabis montbretiana* are confirmed to be sister species and together are sisters to the perennial *A. nordmanniana*. The reconstructed ancestral state for life history (Karl & Koch 2013) has been overlaid on the tree. Expression was studied in species indicated by *. Right: Structural evolution of *FLC* orthologues in the Arabideae. Throughout the branch leading to *A. alpina*, the structure of the locus becomes increasingly more complex. However, there is no relation of locus complexity and life cycle as *A. nordmanniana* loci orthologous to FLC have similar structures to that found in annual *A. montbretiana*. Colours are explained in the legend below the locus schemes. The sequence labelled as COLDAIR RNA encoding was defined by homology to *A. thaliana* (Heo & Sung 2011).

To study the evolution of life history in the Arabideae, a phylogenetic tree was constructed using selected annual and perennial species representing all known major lineages of the Arabideae (Karl *et al*. 2012; Karl & Koch 2013). The phylogenetic tree based on four nuclear and two plastidic markers was fully resolved, and all splits were well supported (Fig. 1).

Results indicate that the species aggregate containing the perennial model *A. alpina* is sister to the annuals *A. montbretiana* and *A. nova* subsp. *iberica*. Moreover, perennial *A. nordmanniana*, which represents four to five perennial polycarpic species (Karl & Koch 2013), is sister to the clade containing *A. alpina* and *A. montbretiana*. Thus, the tree is consistent with previous studies (Karl *et al*. 2012; Karl & Koch 2013) but is based on additional phylogenetic markers and incorporates all annual and perennial species of interest in one analysis. In addition, life history was plotted on the phylogeny for each taxon used in the phylogenetic reconstruction (Figs 1 and S2, Supporting information).

This analysis is consistent with perenniality being the ancestral state and annuality having evolved independently in several lineages due to severe environmental change, as proposed previously (Karl & Koch 2013), but does not unequivocally exclude the possibility that annuality was ancestral in exceptional cases.

Structural evolution of *FLC* orthologues in the Arabideae. *FLC* orthologues in perennial *Arabidopsis* and *Arabis* species exhibit more complex gene structures when compared to annual *Arabidopsis thaliana* (Wang *et al*. 2009; Nah & Chen 2010; Albani *et al*. 2012; Kemi *et al*. 2013). Consistent with this observation, *A. alpina PEP1* has a complex, duplicated gene structure (Wang *et al*. 2009; Albani *et al*. 2012). Therefore, a mechanistic link might exist between complexity of the *FLC*/*PEP1* gene and life history. To further test this hypothesis, *FLC* orthologues were examined in a wider range of annual and perennial *Arabis* species. Six *Arabis* species (*A. alpina*,* A. purpurea*,* A. montbretiana*,* A. nova* subsp. *iberica*,* A. nordmanniana* and *A. auriculata*) were tested for their vernalization response to determine whether they are likely to harbour functional *FLC* alleles. Plants were exposed to vernalization treatment of 4 °C for 12 weeks at different times after germination and then returned to 20 °C under long photoperiods until flowering. Control plants were not exposed to vernalization but maintained under long photoperiods. Except for *A. purpurea*, all tested taxa showed either an obligate (*A. alpina*,* A. nordmanniana*) or a facultative (*A. auriculata*,* A. montbretiana*,* A. nova* subsp. *iberica*; Table S6, Supporting information) vernalization response and were therefore assumed to harbour at least one active *FLC* orthologue. Furthermore, *A. montbretiana* and *A. auriculata* responded to vernalization from soon after germination, and therefore did not exhibit a detectable juvenile phase (Fig. S3 , Supporting information). Three *FLC* orthologues were sequenced either directly after PCR amplification or after cloning (*A. purpurea* = *ApFLC*,* A. nova* subsp. *iberica* = *AiFLC*,* A. auriculata* = *AauFLC*). Another two *FLC* orthologues were identified in contig assemblies based on Illumina sequence data (*A. montbretiana* = *AmFLC*,* A. nordmanniana* = *AnFLC‐A* and *AnFLC‐B*; two genes because the analysed *A. nordmanniana* accession is tetraploid; for method and result of assemblies, see Supplementary Methods and Results, Supporting information). The structures of the loci were then compared to *PEP1* and plotted on the phylogeny (Fig. 1, right panel). All analysed orthologues comprised seven exons, except *PEP1* in which exon 1 is duplicated (Wang *et al*. 2009; Albani *et al*. 2012). Furthermore, no other duplicated regions were detected in the *FLC* orthologues as determined from the number of obtained PCR fragments or based on the results of blast searches performed on the contig assemblies. Therefore, *AnFLC‐A* and *AnFLC‐B*,* AauFLC*,* AiFLC* and *AmFLC* have simple gene structures, similar to *FLC* in *A. thaliana*, except that on the phylogeny separating *A. auriculata* and the other species, a sequence in the first intron showing homology to *A. thaliana COLDAIR* (Heo and Sung 2011) was duplicated. Therefore, among the analysed *Arabis* species, there is no correlation between *FLC* duplication or complexity and life history.

### Differential expression of *FLC* orthologues isolated from four *Arabis* species correlates with life history

**Figure 2:**
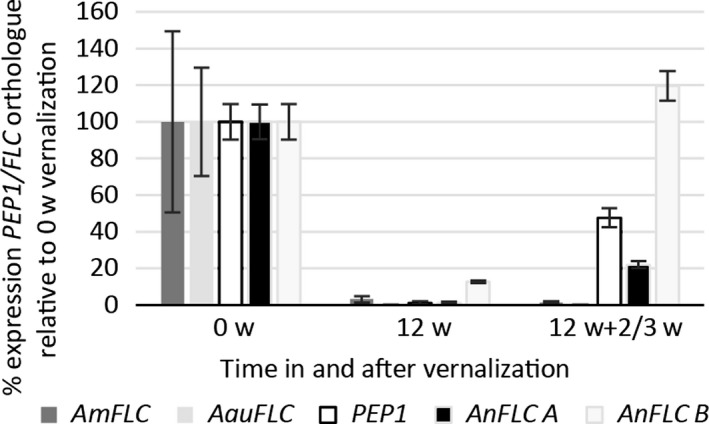
Relative expression of *FLC* orthologues in the perennials *A. alpina* (*PEP1*) and tetraploid *A. nordmanniana* (*AnFLC‐A* and *AnFLC‐B*) as well as in the annuals *A. montbretiana* (*AmFLC*) and *A. auriculata* (*AauFLC*). All examined orthologues are expressed before vernalization (0w = 0 weeks vernalization after growth at warm temperature for 3 or 6 weeks after germination) and repressed by vernalization (12 wv = 12 weeks vernalization at 4 °C). However, only in the perennial species are the *FLC* orthologues derepressed after vernalization because in the annual species, they stay stably repressed (12 wv + 2/3w = 12 weeks vernalization followed by 2 or 3 weeks at warm temperature). Expression values are relative to the respective orthologues of *PP2A* or *RAN3* (*A. nordmanniana*) and relative to 0 weeks (0w) of vernalization. For each sample, leaves of four to six plants were pooled, error bars represent standard deviation of three or four technical qPCR replicates (*n* = 3–4). A biological replicate is given in the supplement (Fig. S4, Supporting information).

Transcription of *PEP1* of *A. alpina* (Wang *et al*. 2009) as well as of the *FLC* orthologues of perennial *Arabidopsis lyrata* (Kemi *et al*. 2013) and *Arabidopsis halleri* (Aikawa *et al*. 2010) is reactivated after vernalization, while in *A. thaliana*,* FLC* is stably repressed upon return to warm temperatures (Bastow *et al*. 2004; Sung & Amasino 2004; Finnegan & Dennis 2007). To evaluate the extent to which *FLC* expression pattern is correlated with life history, we analysed the expression of the *FLC* orthologues of *A. montbretiana*,* A. nordmanniana* (allele specific for *AnFLC‐A* and *AnFLC‐B*) and *A. auriculata* using *PEP1* as a control. In all four species, expression was detected before vernalization and was strongly reduced at the end of vernalization (Figs 2 and S4, Supporting information). Upon transfer to warm temperatures after vernalization, however, only the alleles from perennial species (*AnFLC‐A* and *AnFLC‐B*,* PEP1*) increased in expression, although to different extents, while the alleles from the annual species (*AauFLC* and *AmFLC*) were stably repressed (Figs 2 and S4; Data S1, Supporting information). These data demonstrate that the *FLC* orthologues follow a different expression pattern in annual and perennial *Arabis* taxa, consistent with data obtained from perennial and annual *Arabidopsis* species (Bastow *et al*. 2004; Sung & Amasino 2004; Finnegan & Dennis 2007; Aikawa *et al*. 2010; Kemi *et al*. 2013). *Arabidopsis* and *Arabis* belong to two different evolutionary lineages of the family (Couvreur *et al*. 2010), and therefore, our data demonstrate that a difference in repression of *FLC* orthologues between annual and perennial taxa after vernalization is found widely in the Brassicaceae.

### Construction of hybrids between *A. montbretiana* and *A. alpina* and analysis of the basis of the divergence in *AmFLC* and *PEP1* mRNA patterns

**Figure 3:**
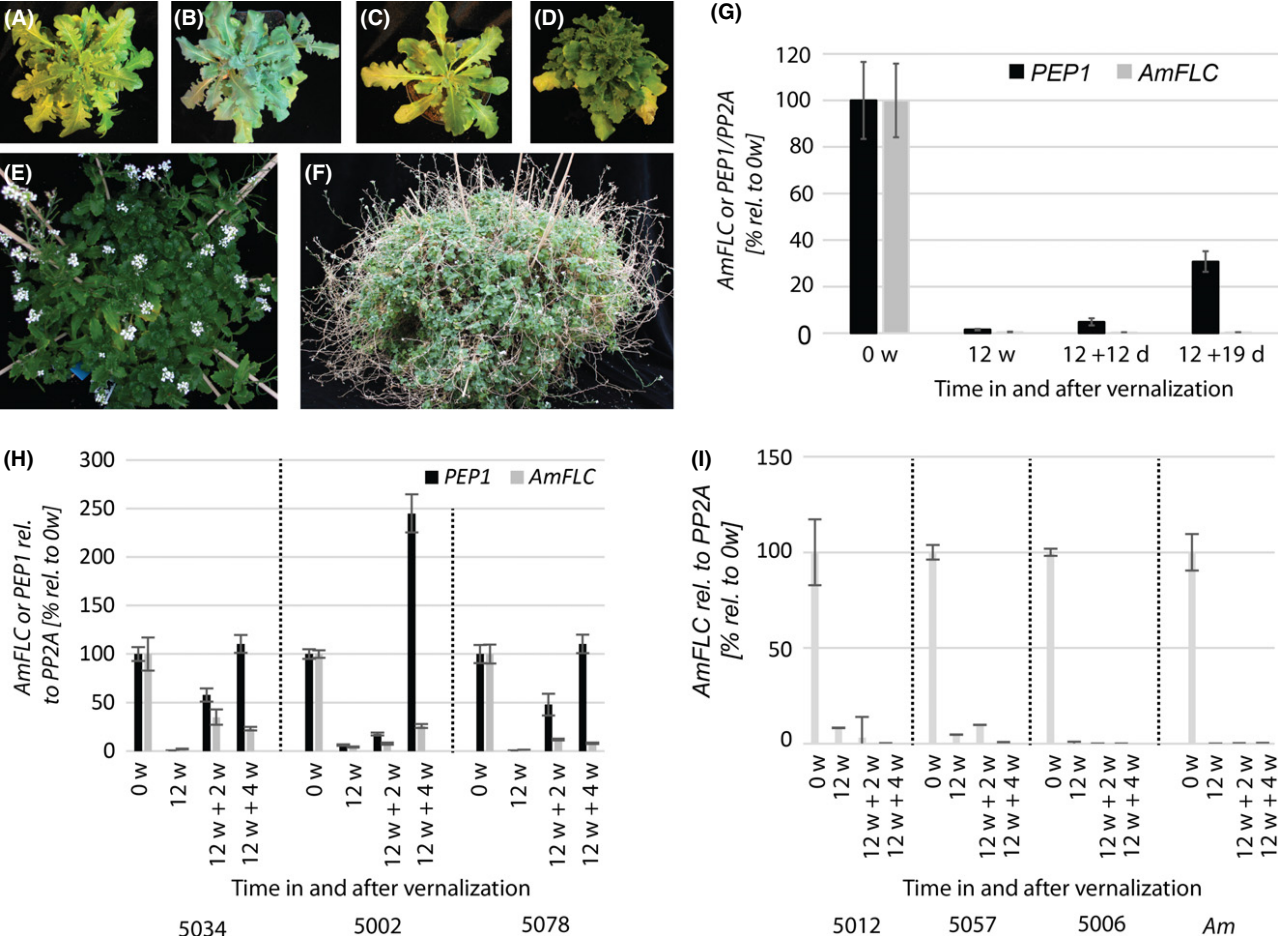
*Arabis montbretiana* (A), *A. alpina* (B) and *A. montbretiana* **×** *A. alpina* (C) at 50 days after germination. D–F *A. montbretiana* **×** *A. alpina* at (D) the onset of flowering at 99 days after germination, (E) fully flowering but not setting viable seeds after self‐pollination 129 days after germination, (F) perpetually growing and flowering 427 days after germination. (G) Relative expression of *AmFLC* and *PEP1* through a vernalization cycle in two cuttings obtained from the hybrid in D–F. Both *FLC* orthologues are expressed before vernalization (0w = 0 weeks) and repressed by vernalization (12w = 12 weeks vernalization). After vernalization and return to warm temperatures (12d = 12 days and 19d = 19 days in warm), *AmFLC* stays stably repressed while *PEP1* rises again in expression. Thus, in the interspecies hybrid, both *FLC* orthologues follow the expression pattern observed in the parents indicating that the differential expression is *cis*‐mediated. Expression is expressed relative to *PP2A* and relative to 0 weeks of vernalization as percentage. Error bars represent the propagated error of the standard deviation calculated for two biological replicates with four technical replicates (*n* = 3–4) each. H‐I Relative expression of *PEP1* and/or *AmFLC* through a vernalization cycle in three introgression lines obtained from back crossing the hybrid in D–F to *A. alpina*. Expression is relative to *PP2A* and then expressed as % relative to expression at 0‐w vernalization. Error bars represent the standard deviation derived from three or four technical (*n* = 3–4) replicates. Different introgression lines represent biological replicates. H Analysis of three independent heterozygous plants (5034, 5002, 5078). The orthologues show different behaviours after vernalization and maintain the same or similar expression pattern that they show in the parental species (Fig. 2). I Analysis of three different *AmFLC* homozygous plants (5012, 5057, 5006). *AmFLC* displays the same stable expression pattern after vernalization as in the *A. montbretiana* control.

To study the regulation of life history as well as the inheritance of *PEP1* and *AmFLC* expression patterns, *A. alpina* acc. Pajares and *A. montbretiana* were crossed. The flowers of *A. montbretiana* were emasculated and cross‐pollinated with *A. alpina* acc. Pajares pollen, but no viable seeds were formed. Therefore, an embryo rescue protocol was applied (Sauer & Friml 2008). By this approach, one plant was successfully grown from approximately 100 cultured immature seeds and its hybrid identity was confirmed by sequencing and length polymorphism markers (Tables S7 and S8, Supporting information). The hybrid was vernalized for 12 weeks to ensure that it flowered. When returned to warm temperatures and long‐day (LD) conditions, the plant flowered abundantly from all shoots (Fig. 3D–F). Axillary shoots were produced from all leaf axils generating a plant that constantly increased in size and flowered perpetually from all branches. Siliques were formed, but all seeds aborted early in development and no viable seeds could be obtained. Therefore, the hybrid could not be reliably scored as annual or perennial because it did not complete the life cycle by forming seeds.

The basis of the differential expression patterns of *PEP1* and *AmFLC* is unknown. The generated hybrid, however, offers the opportunity to test whether the patterns of *AmFLC* and *PEP1* transcription differed due to *cis*‐acting variation by analysing their expression after vernalization in the same genetic background (Wittkopp *et al*. 2004). *AmFLC* and *PEP1* mRNA levels were evaluated in two cuttings before, at the end of and subsequent to a 12‐week‐vernalization cycle using gene‐specific primers designed based on polymorphisms detected between the two genes. Both *PEP1* and *AmFLC* mRNAs were expressed before vernalization and repressed during vernalization (Fig. 3G). However, upon transferring the plants to warm LDs at the end of vernalization, *AmFLC* mRNA remained at very low levels, while abundance of *PEP1* mRNA increased (Fig. 3G). In conclusion, in the interspecies hybrid, each gene exhibits the expression pattern characteristic of the parent from which it originated. This result suggests that *cis*‐acting sequence variation at *PEP1* and *AmFLC* confers the observed difference in expression pattern in the hybrid. To verify this hypothesis, *AmFLC* introgression lines that were largely homozygous for *A. alpina* but retained a genomic segment including *AmFLC* were constructed by backcrossing to *A. alpina* three times and selecting for inheritance of *AmFLC* (Fig. S5, Supporting information). Three plants heterozygous and three plants homozygous for the *AmFLC* genomic segment were exposed to vernalization along with *A. montbretiana* as a control. *AmFLC* was stably repressed after vernalization in the homozygous *AmFLC* introgression line (Fig. 3I) and in the heterozygotes was much more strongly repressed than *PEP1* (Fig. 3H). These results again indicate that both *AmFLC* and *PEP1* responded differently to vernalization when in the same genetic background. Taken together, the analyses of the hybrid and of the introgression lines demonstrate that *cis*‐acting variation contributes to the differential behaviour of *AmFLC* and *PEP1* after vernalization.

### SNPs between *PEP1* and *AmFLC* are distributed nonrandomly

The different expression patterns of *PEP1* and *AmFLC* involve variation in *cis*‐acting elements. To identify polymorphisms that might contribute to the different expression patterns, the nucleotide sequences of the genomic loci were compared. Comparison of the *AmFLC* genomic DNA sequence with the cDNA showed that the protein is encoded by a single gene spanning 7 exons (Fig. S6A, B, Supporting information), as is FLC in *A. thaliana*. Furthermore, alignment of the *AmFLC* cDNA to that of *PEP1* demonstrated that in *A. alpina* acc. Pajares, the splice site at the *PEP1* intron 2–exon 3 junction is shifted, producing an additional 27 bp in the mRNA and therefore a predicted 9 amino acid insertion in the protein. In addition, five nonsynonymous substitutions are present within the coding sequence (Fig. S1, Supporting information).

**Figure 4:**
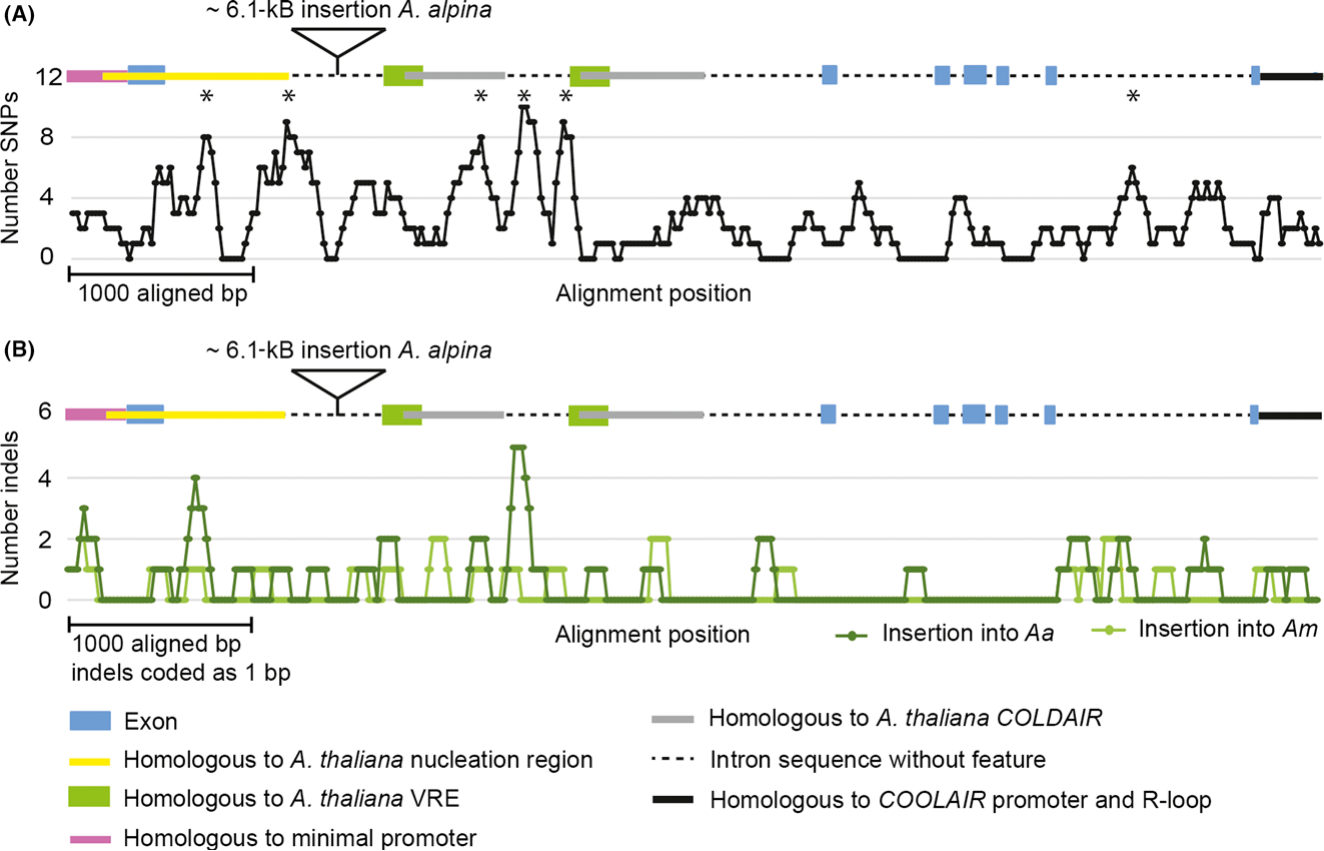
Distribution of SNPs (A) and indels (B) among *PEP1* and *AmFLC* analysed by a sliding window approach (window size 100 bp, shift 20 bp). For the analysis of SNP distribution, all indels were excluded, while for the distribution analysis of indels, all indels were coded as 1 irrespective of their length; exons as well as sequence features homologous to regulatory elements known from *FLC* are annotated on top of the graph as a colour‐coded scheme. Nucleation region is the sequence homologous to the region showing elevated H3K27me3 before vernalization in *Arabidopsis* (Yang *et al*. 2014); COLDAIR represents the regions homologous to the COLDAIR RNA‐encoding region (Heo & Sung 2011); vernalization response element (VRE) indicates the region homologous to the VRE of *A. thaliana* identified in Sung *et al*. (2006); peak regions with windows containing significantly more SNPs than expected in a random distribution are marked by *.

In total, 176 SNPs and 57 indels were detected between *AmFLC* and *PEP1* by comparing the genic regions spanning ~280 bp upstream of the start codon (of exon 1A in *PEP1*; Fig. S6C, D, Supporting information) that include sequences homologous to the functional promoter of *FLC* (Sheldon *et al*. 2002), all exons and introns as well as ~400 bp downstream of the stop codon. This number of SNPs and indels meant that no simple polymorphism could be identified as impairing a *cis*‐acting element conferring the differential expression of *AmFLC* and *PEP1* after vernalization. Therefore, the distribution of SNPs and indels between *AmFLC* and *PEP1* was examined by a sliding window approach to test whether these occurred at higher frequencies in specific genic regions (Fig. 4A, B, Supporting information). Six clusters of SNPs found in this analysis differed significantly (*P* ˂ 0.1) from the expected random distribution (Fig. S7, Supporting information). Five of these clusters were located in the 5′ part of the first intron leading to a higher evolutionary distance of this segment of the intron between *A. alpina* and *A. montbretiana* than for the 3′ part (0.044 vs. 0.017).

To obtain insight into the possible functional relevance of the clustered polymorphisms, the loci were annotated by comparison with *FLC*, in which several *cis*‐regulatory regions have been identified in intron 1 (Fig. 4, Tables S9, S10, S11, S12 and Figs S8, S9, S10, S11, Supporting information). A region showing homology to the nucleation region (Angel *et al*. 2011; Yang *et al*. 2014) (Fig. S11, Supporting information) as well as duplicated regions showing homology to the VRE (Sung *et al*. 2006) (Fig. S10, Supporting information) and the sequence encoding COLDAIR RNA (Heo & Sung 2011) were identified. The duplicated regions showing homology to the VRE and the COLDAIR RNA‐encoding sequence showed 79% (VRE and COLDAIR) identity to each other in *A. alpina* and 73% (VRE) and 77% (COLDAIR) in *A. montbretiana* (Table S12, Supporting information). The six statistically significant peaks in SNP frequency detected in the sliding window analysis coincided with the nucleation region, the region between the nucleation region and the more 5′ located segment showing homology to the VRE and the COLDAIR RNA‐encoding sequence (COLDAIR‐like 1) and the sequence separating COLDAIR‐like 1 from the second segment showing homology to the VRE (Fig. 4A).

Overall, the higher conservation of the 3′ part of intron 1 is consistent with it being required for transcription of *FLC* (Sheldon *et al*. 2002). By contrast, the higher variation in the 5′ part of intron 1, which includes sequences showing homology to the nucleation region and to the COLDAIR RNA‐encoding sequence of *FLC*, suggests that divergence within these segments could be responsible for the species‐specific expression patterns of *PEP1* and *AmFLC*.

### SNP distribution patterns detected by comparing annual and perennial species pairs in different tribes of the Brassicaceae

**Figure 5:**
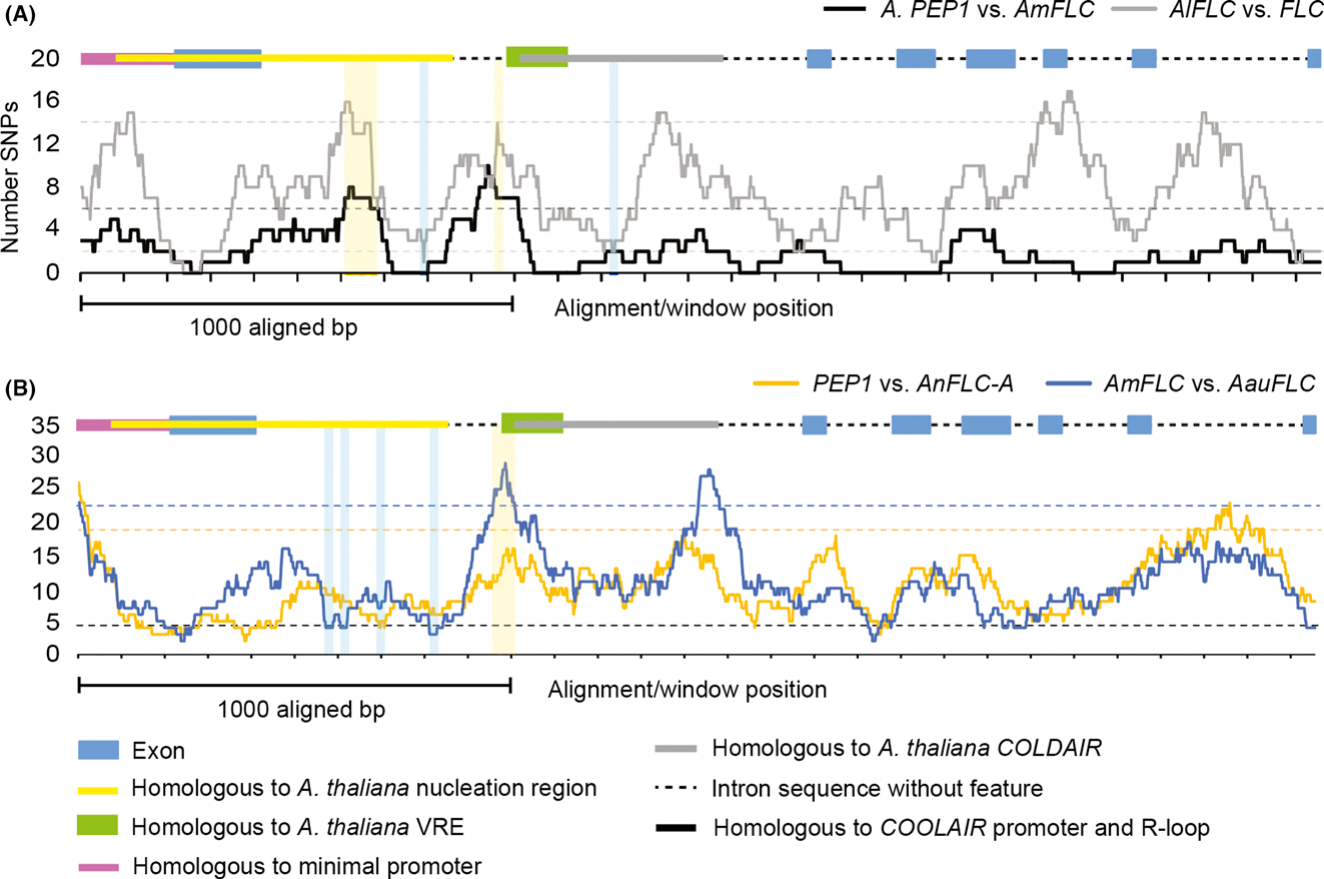
Comparison of SNP distribution in (A) *PEP1* vs. *AmFLC* and *AlFLC1* vs. *FLC* and (B) *AmFLC* vs. *AauFLC* and *PEP1* vs. *AnFLC‐A* by a sliding window analysis (window size 100 bp, shift 1 bp). For the analysis of SNP distribution, all indels were excluded; exons as well as sequence features homologous to regulatory elements known from *FLC* are annotated on top of the graph as a colour‐coded scheme. Exclusion of gap columns leads to deletion of elements which are duplicated only in *PEP1*,* AmFLC* and *AnFLC‐A,* and therefore, the schematic overview of the locus is shorter than the locus schemes in Fig. 4. Nucleation region is the sequence homologous to the region showing elevated H3K27me3 before vernalization in *Arabidopsis* (Yang *et al*. 2014); COLDAIR represents the regions homologous to the COLDAIR RNA‐encoding region (Heo & Sung 2011); vernalization response element (VRE) indicates the region homologous to the VRE of *A. thaliana* identified in (Sung *et al*. 2006); dashed lines represent thresholds above or below which more or fewer SNPs than expected by chance occur: (A) grey dashed line at 14 SNPs threshold for more and at two SNPs for fewer SNPs than expected by chance in *AlFLC1* vs. *FLC*; line at six SNPs threshold for more SNPs than expected by chance for *PEP1* vs. *AmFLC*; no window with significantly fewer SNPs, (B) blue dashed line significantly more SNPs than expected by chance for *AmFLC* vs. *AauFLC*, orange dashed line significantly more SNPs than expected by chance for *PEP1* vs. *AnFLC‐A*, black dashed line fewer SNPs than expected for both curves. Peak regions with windows containing significantly more SNPs than expected in a random distribution are shaded in yellow; regions containing significantly fewer SNPs than expected in a random distribution are shaded in blue.

*FLC* orthologues are also differentially expressed after vernalization in other closely related annual and perennial species, so that, for example, *FLC* is stably repressed after vernalization in *A. thaliana* (Sheldon *et al*. 2000) while the expression of its orthologue from *A. lyrata* (*AlFLC1*) increases again after vernalization (Kemi *et al*. 2013). Therefore, to broaden the analysis of *FLC* orthologues, the genomic DNA sequences of *FLC*,* AlFLC1*,* AmFLC*,* PEP1*,* AnFLC‐A* and *AauFLC* were aligned, gap columns were excluded, and SNP distribution was scored using a sliding window approach. The analysis of *FLC* and *AlFLC1* resulted in a SNP distribution pattern similar to the one described for the comparison of *AmFLC* and *PEP1* (Fig. 5A). Several peaks in SNP frequency containing more SNPs than the expected random distribution were detected in the comparison of each species pair (Fig. 5A). Several of these peaks in SNP frequency are specific to only one of the species comparisons, but two were detected in the same regions in both comparisons. One of the common SNP frequency peaks was detected in a region homologous to the nucleation region (Yang *et al*. 2014), and the second was present upstream of the VRE (Sung *et al*. 2006) (Figs 5A, S19 and S20, Supporting information). Sequence differences in these two regions might contribute to the different expression patterns of *FLC* orthologues detected in annual and perennial species, because they are highly polymorphic in two independent occurrences of divergence of annual and perennial patterns.

**Figure 6:**
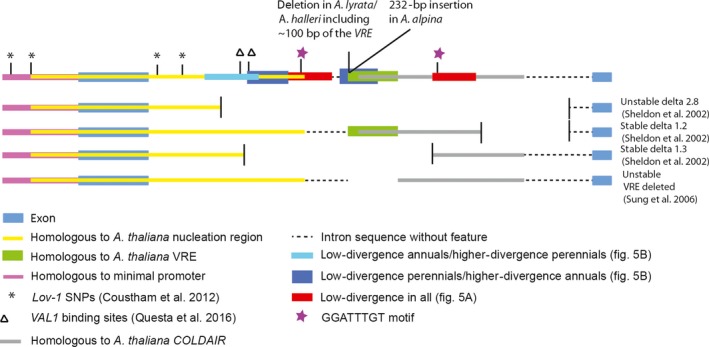
Summary of the regulatory regions of *FLC* and their variation among annual and perennial taxa. Top: Schematic representation of the *FLC* locus of *A. thaliana* including the minimal promoter, exon 1, intron 1 and exon 2 indicating the positions of SNPs, binding sites and regulatory elements that were identified in other studies as well as regions containing putative regulatory elements identified in this study. Lower diagrams depict segments of three reporter gene deletions reported by Sheldon *et al*. (2002) that showed unstable or stable repression after vernalization. The lowest diagram shows a deletion mutant in which *FLC* was not stably repressed after vernalization and defines the VRE (Sung *et al*. 2006).

As a further control to test the functional significance of the two regions described above, we also examined whether they are highly polymorphic between related species that do not differ in life history. To this end, SNP frequencies were analysed in comparisons of the *FLC* orthologues of the closely related annual taxa *A. montbretiana* and *A. auriculata* as well as the closely related perennials *A. alpina* and *A. nordmanniana* (Fig. 5B). The peaks in SNP frequency obtained in these analyses were also compared to those detected in Fig. 5A. The region adjacent to the 5′ end of the VRE, which was highly polymorphic in the comparisons of the annual and perennial species (Fig. 5A), was also detected as variable in comparisons of *FLC* orthologues of the two annual species but not in comparing the two perennial species (Figs 5B and S20, Supporting information). Therefore, this sequence could be functionally conserved in perennials and diverge independently in annuals, so that it contains a high frequency of SNPs in comparisons of annuals and perennials or of annuals. By contrast, the polymorphic segment detected in the nucleation region by comparing annuals and perennials (Fig. 5A) showed fewer SNPs than expected in a random distribution when the orthologues of either the two genes from perennials (*AnFLC‐A* and *PEP1*) or the two genes from annuals (*AmFLC* and *AauFLC*) were compared (Figs 5B, S22 and S23, Supporting information). Variation in this segment of the nucleation region at the 5′ end of intron 1 could therefore be involved specifically in the divergence of annual and perennial patterns of regulation of *FLC* orthologues. Our sequence analysis of *FLC* orthologues from the Arabideae and Camelineae therefore identifies this region in the nucleation region and another at the 5′ end of the VRE as likely to be involved in the divergence of annual and perennial patterns of expression of FLC orthologues (Figs 6, S19 and S20, Supporting information).

### Detection of common regulatory elements

The regions harbouring significantly fewer SNPs than expected might define regions that are important for *FLC* regulation in both annuals and perennials. These regions were therefore examined in all four alignments shown in Fig. 5A, B. No regions harbouring fewer SNPs than expected were detected in comparisons of *AmFLC* and *PEP1*, perhaps because many of the sequence windows in this comparison were 100% identical. However, the analysis of *FLC* and *AlFLC1* identified six regions harbouring fewer SNPs than expected, and three of these regions were located in intron 1. The first was located at the end of the nucleation region, the second in the region homologous to the COLDAIR RNA‐encoding sequence and the third at the very end of the first intron (Figs 5A and S21, Supporting information). The comparison of *AmFLC* and *AauFLC* also detected the segment at the end of the nucleation region as harbouring fewer SNPs than expected (Fig. 5B). Interestingly, comparison of sequences of *FLC* and *AlFLC1* in the segment homologous to the nucleation region and the COLDAIR RNA‐encoding sequence revealed that each segment includes the motif GGATTTGT and both of these motifs are perfectly conserved in all six species analysed (Fig. S21, Supporting information). Therefore, this sequence element could have a common function in *FLC* regulation across all taxa (Fig. 6).

## Discussion

To study the divergence of life history, a phylogeny of 17 annual and perennial species from the tribe Arabideae was constructed using a combination of six different phylogenetic marker systems. This highly resolved and supported phylogeny was congruent to one that previously fully resolved the backbone of the tribe (Karl & Koch 2013), but the phylogeny described here also included *D. nemorosa* and *A. nova* subsp. *iberica*. These species were grouped together with their previously described relatives, which are other *Draba* species and *A. montbretiana*, respectively (Jordon‐Thaden *et al*. 2010; Karl *et al*. 2012). The phylogeny of the Arabideae is therefore robust and provides a framework for studying the evolution of annual and perennial life history, which have diverged within several of the established clades.

### Evolution of *FLC* structure

Remarkable sequence variation at *FLC* genes occurs within and between Brassicaceae species, and increasing complexity of these loci has been suggested to be linked to perenniality in *A. alpina* and in *Arabidopsis* species (Alonso‐Blanco & Méndez‐Vigo 2014). Indeed, through the phylogenetic branch leading from annual *A. auriculata* to *A. alpina*, the orthologous *FLC* loci increased in complexity. *AauFLC* showed a similar simple structure to *FLC* of *A. thaliana*, while *PEP1* from perennial *A. alpina* acc. Pajares was the most complex locus incorporating a partial duplication that includes exon 1 and generates two overlapping transcripts (Albani *et al*. 2012). Furthermore, *AmFLC* and *AiFLC* from annual *A. montbretiana* and *A. nova* subsp. *iberica* showed simple structures similar to *A. thaliana FLC*, further strengthening this hypothesis. However, in perennial tetraploid *A. nordmanniana*, single‐copy *AnFLC* genes were found that showed simple exonic structures similar to *FLC*, although they contained a duplication of part of intron 1 related to COLDAIR (Heo and Sung 2011) that was also present in *AmFLC* and *AiFLC*. Therefore, at least in the Arabideae, there does not seem to be a strict association between gene or exon duplication at the *FLC* locus and perenniality.

### Correlation between annualism and *FLC* expression pattern

In perennials, flowering is strongly repressed at particular times during the life cycle, ensuring episodes of vegetative and reproductive development, whereas annuals usually flower more rapidly and do not return to vegetative development. In the Brassicaceae, the vernalization response pathway was proposed to play several roles in conferring these episodes of vegetative and reproductive development in the context of the perennial life cycle (Wang *et al*. 2009; Aikawa *et al*. 2010; Kemi *et al*. 2013). Furthermore, annual and perennial species in the Brassicaceae also differ in the age at which they acquire competence to flower in response to vernalization (Wang *et al*. 2011; Bergonzi *et al*. 2013; Zhou *et al*. 2013). Consistent with this conclusion, *A. montbretiana* and *A. auriculata* behaved in a similar way to annual *A. thaliana* and flowered in response to vernalization treatments given within 1 week of germination. Thus, the annuals *A. montbretiana*,* A. auriculata* and *A. thaliana* show similar flowering responses and differ from perennial *A. alpina* in that floral induction through vernalization is less restrained by age.

In *A. thaliana*,* FLC* expression is stably repressed during vernalization, so that upon return to warm temperatures, its expression stays low ensuring that the plant is stably induced to flower (Michaels & Amasino 1999; Bastow *et al*. 2004). By contrast, in several perennial Brassicaceae species, levels of expression of orthologues of *FLC* cycle in response to temperature, so that they fall in cold and rise again in subsequent periods of warm (Wang *et al*. 2009; Aikawa *et al*. 2010; Kemi *et al*. 2013). Thus, the cycling pattern of *FLC* expression correlates with episodes of vegetative growth and flowering in the perennials. The functional significance of these patterns was demonstrated genetically in *A. alpina* (Wang *et al*. 2009; Albani *et al*. 2012). The *FLC* orthologues of annuals *A. montbretiana* and *A. auriculata* were also found to be stably repressed after vernalization, similar to the pattern of expression of *FLC* found in *A. thaliana* and contrasting with the perennial pattern of *PEP1* regulation in *A. alpina* and *A. nordmanniana*. In tetraploid *A. nordmanniana*, both *FLC* orthologues were found to be upregulated to different extents after vernalization, which might be a *cis*‐mediated effect caused by any of the numerous SNPs that differ between the two alleles. Thus, consistent with their flowering behaviour, annual and perennial species differ in the patterns of expression of *FLC* orthologues. This stable repression in response to vernalization must have evolved independently more than once during the diversification of the Arabideae and in the *Arabidopsis* genus.

### Evolution of *FLC* regulation among annual and perennial Brassicaceae species

Differences in gene expression arise during evolution through *cis*‐acting changes in the gene of interest or by alterations in the activity of *trans*‐acting factors that contribute to the regulation of its expression (Emerson & Li 2010). These possibilities can be distinguished by the construction of hybrids, in which both orthologues of the gene of interest are present but the genetic background encoding *trans*‐acting factors is identical (Wittkopp *et al*. 2004; de Meaux *et al*. 2005). If the orthologues show different expression patterns in the parental species and these are retained in the hybrid, then *cis*‐acting changes must contribute to the differential expression. In the hybrid of *A. montbretiana* and *A. alpina* as well as in introgression lines derived from the hybrid, *PEP1* and *AmFLC* were expressed in different patterns after vernalization, as observed in the parental species. Therefore, the differential expression must involve *cis*‐acting changes. Scans of SNP frequency were performed between *FLC* orthologues to search for putative regulatory elements that have diverged between annual and perennial species and to identify conserved sequences that may have a common function in *FLC* regulation in annual and perennial taxa.

#### Expression of FLC in warm temperatures

Transgenic approaches based on deletion analysis of a *FLC::GUS* transgene demonstrated that 272 bp of promoter as well as ~200 bp at the 5′ end and ~525 bp at the 3′ end of intron 1 are sufficient for transcription of *FLC* before vernalization (Sheldon *et al*. 2002). Our sequence comparisons included 295 bp of *FLC* sequence upstream of the ATG, and the comparisons of *FLC* and *AlFLC1* or *PEP1* and *AnFLC‐A* identified one region containing significantly more SNPs than expected. No known motifs were present in this region. However, the 272‐bp minimal promoter of *FLC* contained three putative bZIP recognition motifs (Sheldon *et al*. 2002) of which only one is highly conserved in all six taxa included in the analysis and is therefore likely to be functionally important. A later study reported a binding motif that is involved in recognition of the *FLC* promoter by SUF4, a component of the FRIGIDA complex that increases *FLC* transcription in *A. thaliana* (Choi *et al*. 2011). However, the binding motif, which is located upstream of the minimal promoter (Sheldon *et al*. 2002), was mutated or absent in *A. alpina* and entirely absent in *A. montbretiana*. Therefore, either regulation of *FLC* by the FRIGIDA complex does not occur in the *Arabis* genus or the binding site for the SUF4 complex is not conserved.

#### Repression of FLC by vernalization

The same segments of intron 1 required for expression of *FLC* prior to vernalization were also described as being important for its repression by vernalization (Sheldon *et al*. 2002). Later studies demonstrated that the 5′ end of intron 1 overlaps with a region referred to as the nucleation region, at which H3K27 accumulates most rapidly and to high levels during vernalization (Yang *et al*. 2014). The same region contains two RY elements which act as binding sites of the transcriptional repressor VAL1 that nucleates silencing at the *FLC* locus by interacting with the apoptosis‐ and splicing‐associated protein (ASAP) complex and maybe Polycomb Repressive Complex 1 (PRC1) (Qüesta *et al*. 2016; Yuan *et al*. 2016). One of the RY elements was perfectly conserved across all six taxa in our analysis, consistent with a highly conserved function, while the second one was mutated in *A. alpina*, but only in one of the two tandem copies of the first exon. Sequence variation in the nucleation region was proposed to cause differences in the kinetics of vernalization because four SNPs in the *A. thaliana* accession *Lov‐1* were identified in this region, including two in the 5′ end of intron 1, and shown to be associated with requirement for an extended vernalization period to cause silencing of *FLC* after vernalization (Coustham *et al*. 2012). The first SNP in intron 1 was conserved among the *Lov‐1* accession and all taxa included in this study, and only the *FLC* allele of *A. thaliana Columbia* was different, suggesting that among *A. thaliana* accessions, the Lov‐1 SNP is ancestral. The second SNP in intron 1 was variable across taxa. In this position, *Lov‐1*,* PEP1* and *AmFLC* were the same, while *AnFLC‐A*,* AlFLC1*,* AauFLC* and *FLC* from *A. thaliana Columbia* shared the same base in this position. No regions with significantly more or fewer SNPs than expected in a random distribution were found to overlap with the two SNPs in intron 1 detected in the study of the Lov‐1 accession (Qüesta *et al*. 2016). However, the more 5′ VAL1 binding site was located within the region harbouring fewer SNPs than expected in annuals, while the second VAL1 binding site was located in the overlap of the regions harbouring fewer SNPs than expected in annuals or perennials (Fig. 6). The antisense RNA COOLAIR which plays an initial role in downregulation of *FLC* in response to cold (Swiezewski *et al*. 2009) is conserved in *A. alpina* (Castaings *et al*. 2014).

#### Maintenance of FLC repression after vernalization

Deletion analysis identified four regions within the first intron of *FLC* that are required for stable repression of transcription after vernalization (Sheldon *et al*. 2002). Another study identified the vernalization response element (VRE) whose deletion leads to unstable repression of *FLC* (Sung *et al*. 2006). Subsequently, parts of the VRE were reported to act as the promoter of a noncoding sense RNA named COLDAIR that interacts with components of the PRC2 complex in vitro (Heo and Sung 2011). The COLDAIR RNA‐encoding sequence also included one of the candidate regions for conferring stable repression of *FLC* after vernalization identified by Sheldon *et al*. (2002). However, there are some contradictions between the reporter construct data (Sheldon *et al*. 2002) and the analysis of the VRE mutant (Sung *et al*. 2006) that are summarized in Fig. 6. The comparison of *AlFLC1* and *FLC* identified two regions within the first intron that contained fewer SNPs than expected in a random distribution. One of the regions was also found in the comparison of *AauFLC* and *AmFLC*, whereas in *PEP1* and *AmFLC* comparisons, no fragments harbouring fewer SNPs than expected were found. Identification of the same region in two sequence comparisons in different evolutionary lineages, Camelineae and Arabideae, indicates that this region may be of general importance in the regulation of *FLC* and its orthologues. Interestingly, both conserved regions in the *A. thaliana* comparison with *A. lyrata* contained the same perfectly conserved sequence motif (GGATTTGT), and these were also present in all four *Arabis* species. The deletion analysis of Sheldon *et al*. (2002) is consistent with a role for this highly conserved ‘GGATTTGT’ motif in stable repression of *FLC*. In this study, deletion of the region including both ‘GGATTTGT’ motifs caused unstable repression after vernalization, while constructs where only one copy of the motif was deleted were still stably repressed after vernalization. Thus, these motifs might act redundantly in conferring stable repression of *FLC* by vernalization. If these motifs do contribute to stable repression of *FLC* in annual *A. thaliana*, then perennial taxa such as *A. alpina* must also contain some of the motifs contributing to stable repression in annuals.

#### Reactivation of FLC orthologues after vernalization

No *cis*‐regulatory elements have been described that are involved in the reactivation of *FLC* orthologues in perennials after vernalization. Unstable repression of *FLC* occurs upon deletion of the VRE (Sung *et al*. 2006), and therefore, a loss of function of the VRE in the perennial lineages could confer reactivation after vernalization. However, this is apparently in contradiction to the hypothesis that perenniality is the ancestral state. Nevertheless, parts of the VRE are indeed deleted in *A. halleri* and *A. lyrata,* and in *A. alpina,* a 232‐bp insertion is found at the 5′ end of the VRE. On the other hand, although expression *of AnFLC‐A* and *AnFLC‐B* was unstable after vernalization, its VRE sequence contained no insertions or deletions compared to that of *FLC*. The SNP scans and statistical tests identified regions that harbour significantly more or fewer SNPs than expected by chance. The combination of SNP scans comparing annual and perennial taxa and SNP scans within pairs of closely related annual and perennial taxa identified a segment within intron 1 at the 3′ end of the nucleation region that was particularly divergent within the annual–perennial pairs but more strongly conserved among annuals in one segment and among perennials in another segment. Therefore, this region might contain at least two regulatory elements that vary during evolution and one of which could be related to reactivation of *FLC* after vernalization (Fig. 6). The identified region could be tested by transgenic approaches to directly examine its functional significance.

A possible hypothesis from these studies is that perennials contain two types of regulatory elements that confer stable repression or reactivation of *FLC* orthologues after vernalization. The annual expression pattern could then evolve several times independently by recurrent deactivation of a perennial element required for reactivation. Thus, mutation would provide a flexibility in regulation of *FLC* orthologues that could rapidly evolve along with shifts from perennial to annual life history. The phylogenetic framework and sequence analysis presented here provide a basis for further testing of this hypothesis.

C.K. and G.C. designed research and wrote manuscript; G.C. and M.K. provided plant material; C.K. and S.B. performed vernalization shift experiment; C.K. helped in DNA extraction, genotyping, expression analysis, estimates on evolutionary divergence, SNP distribution analysis, sequencing of *FLC* orthologues, *A. nordmanniana* assembly, amplification of phylogenetic marker sequences, generation and verification of interspecies hybrid, *A. montbretiana* contig assembly and *AmFLC* contig detection and analysis; R.K. and C.K. involved in alignment and phylogenetic analysis; E.S. and C.K. involved in generation of length polymorphism markers; C.K. involved in A. montbretiana and A. nordmanniana contig assembly and FLC contig detection; E.S. involved in preparation of *A. montbretiana* assembly for submission, *A. nordmanniana* read preparation, genome annotation, k‐mer analysis, ortho‐MCL analysis; A.T. helped in statistical test on SNP distribution.

## Data accessibility

Whole‐genome shotgun projects: deposited at GenBank under the Accession nos LNCG00000000 (*A. nordmanniana*) and LNCH00000000 (*A. montbretiana*).

DNA sequences: KC814706;JQ919880;GU181934;GU181999;JQ919835;JQ919813;JQ919857;KC814698;JQ919879;HM046191;HM046243;HM046216;KC814699;JQ919881;GU182084;GU181952;GU182017;KC814700;JQ919882;KC814707;JQ919884;HQ646785;HQ646724;FJ187937;FJ188234;FJ188083;KC814701;JQ919885;HM046193;HM046245;HM046218;KC814708;JQ919886;KF547353;KF547826;KF547624;KC814702;JQ919887;JQ919839;JQ919817;JQ919861;KC814709;JQ919888;KC814703;JQ919889;GU181938;GU182003;GU182089;GU181957;GU182022;KC814713;JQ919899;JQ919840;JQ919818;JQ919862;KC814704;JQ919890;HQ646642;HQ646763;HQ646702;KC814705;JQ919891;KF547181;GU202868;GU202800;KC814710;JQ919893;JQ919844;JQ919822;JQ919866;KC814711;JQ919894;JQ919845;JQ919823;JQ919867;KC814712;JQ919896;KU525030;KU525030;KU525030;KU525030;KU525030;KU525030;KU321504;KU321505;KU321506;KU321507;KU321508;KU321509;KU321510;KU321511;KU321512;KU321513;KU321514;KU321515;KU321516;KU321517;KU321518;KU321519;KU321520;KU321353;KU321354;KU321355;KU321356;KU321357;KU321358;KU321359;KU321360;KU321361.

## Supporting information


**Table S1** Details of accessions used in this study stating country of origin, provider of seeds and sequences used in the phylogenetic analysis and their genebank accession numbers; for the pairs of grey shaded accessions marker sequences were used in combination to obtain a full set for the phylogenetic reconstructions.
**Table S2** Primers used in this study for amplification of phylogenetic marker sequences, qPCR and amplification of the mRNA of FLC orthologues as well as the genomic loci.
**Table S3** Alignment of marker sequences used for phylogenetic analysis; the alignment is given in nexus format and partitions are indicated at the end of the file.
**Table S4** Results of Model Testing and Bayesian analyses for the individual single marker dataset and the combined dataset
**Table S5** Alignment of marker sequences (ADH, CHS, *At2g13360*) used for the maximum parsimony analysis including both co‐orthologues of *A. nordmanniana* for each marker. Introns were not included in the analysis and therefore deleted.
**Table S6** Vernalization response of selected Arabideae; plants were grown either in long day for several months or in long day for 6 weeks, transferred into vernalization for 12 weeks and then returned to warm temperatures and long day conditions.
**Table S7** Polymorphic sites in *Arabis alpina* Pajares and *Arabis montbretiana* and the polymorphisms found in the hybrid individual in the ITS confirming the hybrid identity of the plant.
**Table S8** Primers used for genotyping the F1 plant and introgression lines carrying a genomic fragment including *AmFLC*.
**Table S9** Number of SNPs and indel polymorphisms detected in different features of *PEP1* versus *AmFLC*,* Am* = *A. montbretiana*,* Aa* = *A. alpina*,* At* = *A. thaliana* (exons, regulatory sequences known from *FLC* (proximal promoter (Sheldon *et al*. 2002); nucleation region is the sequence homologous to the region showing elevated H3K27me3 before vernalization in *Arabidopsis* (Yang *et al*. 2014); *COLDAIR* is the regions homologous to the COLDAIR RNA encoding region (Heo & Sung 2011); Vernalization Responsive Element (VRE) indicates the region homologous to the VRE of *A. thaliana* identified in (Sung *et al*. 2006)).
**Table S10** Alignment of the *PEP1*,* AmFLC*,* AlFLC1* and *FLC* loci comprising a partial sequence of the next upstream gene or its orthologues from *A. thaliana* (*At5 g10150*), all intergenic sequence between *At5 g10150* and its orthologues and *FLC* and orthologues as well as all sequences orthologues/homologous to exons and introns of *FLC* (including indels) and approximately 500 bp of sequence downstream of the stop codon.
**Table S11** Summary of the comparison of *AtFLC*,* AmFLC, AlFLC1* and *AaPEP1*.
**Table S12** Comparison of *VRE*/*VRE‐like* and *COLDAIR*/*COLDAIR‐like* sequences found in the first intron of *PEP1* and *AmFLC*; intron 1 in *A. alpina* refers to the sequence homologous to *A. montbretiana* intron 1.
**Table S13** Associated *p*‐values for the respective SH tests based on the different single marker datasets.
**Table S14** Alignment of both *FLC* orthologues from *A. nordmanniana* (*AnFLC A* and *B*) to *PEP1* and *AmFLC*; a stretch of N was inserted between the two contigs joined to form *AnFLC A*. The alignment includes a partial sequence of the orthologue of *At5 g10150*, exons 1 (A and B in *A. alpina*) to 7 including all intronic sequences (partial intron 6 in *AnFLC A*) as well as ~280‐bp sequence downstream of the stop codon (includes the COOLAIR promoter (Castaings *et al*. 2014; Csorba *et al*. 2014; Swiezewski *et al*. 2009).
**Fig. S1** Alignment of cDNA sequences of *AmFLC*,* AauFLC* and *PEP1* used for designing primers for qPCR.
**Fig. S2** Maximum Parsimony (MP) phylogenetic reconstruction calculated in MEGA5 (Tamura *et al*. 2011) based on the orthologues of *ADH*,* CHS* and *At2g13360*.
**Fig. S3** Vernalization response of (A) *A. montbretiana* and (B) *A. auriculata* measured in leaf number.
**Fig. S4** Biological replicates of expression data shown in Fig. 2.
**Fig. S5** Genotypes of introgression lines (*A. montbretiana* in *A. alpina* acc. Pajares background) used in the expression analysis of *AmFLC* and *PEP1* for confirming the results obtained from the two cuttings of the original F1 plant (*A. montbretiana* x *A. alpina* acc. Pajares).
**Fig. S6** (A) Dot plot of the *AmFLC* genomic region (last exon upstream gene until the end of the COOLAIR promoter) vs. *AmFLC* cDNA; 7 exons can be detected, no exon has been duplicated (B, C, D) Comparison of the *PEP1*,* AmFLC* and *FLC* sequences by GATA plots.
**Fig. S7** Statistical test on the SNP distribution in the alignment of *AmFLC* and *PEP1*.
**Fig. S8** Alignment of the proximal promoter region including UTR of *FLC*,* AmFLC* and *PEP1*; the proximal promoter is defined as the region homologous to what was described as *FLC* minimal promoter (Sheldon *et al*. 2002).
**Fig. S9** Alignment of *COLDAIR* and *A. montbretiana* and *A. alpina COLDAIR ‐like 1* and *COLDAIR ‐like 2*; yellow positions indicate ancestral positions maintained in *COLDAIR ‐like 1* while pink positions indicate conservation in *COLDAIR ‐like 2*; more ancestral positions are maintained in *COLDAIR ‐like 1*; COLDAIR‐likes are defined as regions showing homology to the *Arabidopsis* COLDAIR sequence (Heo & Sung 2011).
**Fig. S10** Alignment of *VRE* and *A. montbretiana* and *A. alpina VRE ‐like 1* and *VRE ‐like 2*; VRE‐like sequences are defined as sequences showing homology to the Vernalization Response Element of *FLC* in *Arabidopsis thaliana* (Sung *et al*. 2006).
**Fig. S11** Alignment of the *A. montbretiana* and *A. alpina* region homologous to the nucleation region in *A. thaliana* defined by the ChIP primers from (Yang *et al*. 2014).
**Fig. S12** *Arabis montbretiana* life cycle monitored under greenhouse conditions.
**Fig. S13** Alignment of *PEP1* exon 1A and 1B and *AmFLC* exon 1 along with adjacent intron sequences; the 416 bp used to differentiated copy 1A and copy 1B are indicated by $ while the SNPs differentiating the duplicated segment of intron 1 in PEP1 are marked by *
**Fig. S14** Kmer abundance distribution.
**Fig. S15** Ends of concatenated contigs which were found to show homology to *FLC* and which were shown to be part of the same molecule (*AnFLC B*) based on PCR and subsequent Sanger sequencing; All contigs showed overlapping ends.
**Fig. S16** Statistical test on the SNP distribution in the alignment of (A) *AmFLC* and *PEP1* and (B‐C) *FLC* and *AlFLC1*. The test measures whether the observed SNP number in a window is different from a random distribution (A and B more SNPs than expected, C fewer SNPs than expected); the left panel shows the number of SNPs per window, the right panel shows the –log10 of the associated p‐values; the dashed line is the threshold above which statistical significance was reached; values were corrected for FDR.
**Fig. S17** Statistical test on the SNP distribution in the alignment of (A) *AnFLC‐A* and *PEP1* and (B) *AauFLC* and *AmFLC*.
**Fig. S18** Statistical test on the SNP distribution in the alignment of (A) *AnFLC‐A* and *PEP1* and (B) *AauFLC* and *AmFLC*.
**Fig. S19** Sequence alignment of six Brassicaceae species in the segment showing more SNPs than expected by chance in the nucleation region from Fig. 5A.
**Fig. S20** Sequence alignment of six Brassicaceae species in the segment showing more SNPs than expected by chance upstream/in the region homologous to the VRE from Fig. 5A.
**Fig. S21** Sequence alignment of six Brassicaceae species in the segment showing fewer SNPs than expected in a random distribution in the comparison of *AlFLC1* and *FLC* showing homology to (A) the end of the nucleation region and (B) COLDAIR from Fig. 5A; the conserved GGATTTGT‐motif is indicated a red square.
**Fig. S22** Sequence alignment of six Brassicaceae species in the segment showing fewer SNPs than expected by chance within the region showing homology to the nucleation region in the annual couple (*AauFLC‐AmFLC*) from Fig. 5B.
**Fig. S23** Sequence alignment of six Brassicaceae species in the segment showing fewer SNPs than expected by chance within the region showing homology to the nucleation region in the perennial couple (*PEP1‐AnFLC‐A*) from Fig. 5B

**Data S1** Ct values and evaluation of qPCR data
