# Supplementary material for: Divergence of annual and perennial species in the Brassicaceae and the contribution of cis‐acting variation at FLC orthologues
Source: Mol Ecol. 2017 Mar 22;26(13):3437–57. doi: 10.1111/mec.14084 (PMC5485006; doi:10.1111/mec.14084)
Supplement: Supplementary file 1 — Table S1 Details of accessions used in this study stating country of origin, provider of seeds and sequences used in the phylogenetic analysis and their genebank accession numbers; for the pairs of grey shaded accessions marker sequences were used in combination to obtain a full set for the phylogenetic reconstructions. Table S2 Primers used in this study for amplification of phylogenetic marker sequences, qPCR and amplification of the mRNA of FLC orthologues as well as the genomic loci. Table S3 Alignment of marker sequences used for phylogenetic analysis; the alignment is given in nexus format and partitions are indicated at the end of the file. Table S4 Results of Model Testing and Bayesian analyses for the individual single marker dataset and the combined dataset Table S5 Alignment of marker sequences (ADH, CHS, At2g13360) used for the maximum parsimony analysis including both co‐orthologues of A. nordmanniana for each marker. Introns were not included in the analysis and therefore deleted. Table S6 Vernalization response of selected Arabideae; plants were grown either in long day for several months or in long day for 6 weeks, transferred into vernalization for 12 weeks and then returned to warm temperatures and long day conditions. Table S7 Polymorphic sites in Arabis alpina Pajares and Arabis montbretiana and the polymorphisms found in the hybrid individual in the ITS confirming the hybrid identity of the plant. Table S8 Primers used for genotyping the F1 plant and introgression lines carrying a genomic fragment including AmFLC. Table S9 Number of SNPs and indel polymorphisms detected in different features of PEP1 versus AmFLC, Am = A. montbretiana, Aa = A. alpina, At = A. thaliana (exons, regulatory sequences known from FLC (proximal promoter (Sheldon et al. 2002); nucleation region is the sequence homologous to the region showing elevated H3K27me3 before vernalization in Arabidopsis (Yang et al. 2014); COLDAIR is the regions homologous to the COLDAIR RNA enc [file MEC-26-3437-s001.pdf]

Supplementary Material

Divergence of annual and perennial species in the Brassicaceae and the contribution of cis-acting variation at *FLC* orthologues

C Kiefer[1], E Severing[1], R Karl[3], S Bergonzi[2], M Koch[3], A Tresch[1,4], G Coupland[1,5]

[1] Max Planck Institute for Plant Breeding Research, Plant Breeding Research, Plant Developmental Biology, Carl-von-Linné Weg 10, 50829 Cologne, Germany

[2] Wageningen UR Plant Breeding, Wageningen University and Research Centre, Droevendaalsesteeg 1, 6708PB, Wageningen, The Netherlands

[3] Centre for Organismal Studies, Department of Biodiversity and Plant Systematics, INF 345, 69120 Heidelberg, Germany

[4] University of Cologne, Cologne Biocenter, Zülpicher Str. 47b, 50674 Cologne, Germany

key words: flowering time, annual, perennial, *Arabis*, *PEP1*, *FLC*

[5] **Author for correspondence**: George Coupland, Max Planck Institute for Plant Breeding Research, Plant Developmental Biology, Carl-von-Linné Weg 10, 50829 Cologne, Germany, telephone 0049-221-5062-205, fax 0049-221-5062-207, coupland@mpipz.mpg.de

- Supplementary Methods and Results p. 2 - 6

- Supplementary Figures and Tables p. 7 - 51

SUPPLEMENTARY METHODS AND RESULTS

Plant cultivation.

GH1:

photoperiod 16h, photoperiod extended and light supplemented if necessary (light source Osram HQI-BT 400W/D (white), NAV400W (orange/red)), 20 °C day and 18°C night, soil produced by Balster, Sinntal-Altengronau, Germany (Type Mini-Tray with 1kg /m³ compound fertilizer + 1kg /m³ Osmocote start 8 weeks (Scouts)), plants were watered daily and fertilized once per week with Wuxal Super 8-8-6 diluted to 4%.

GH2:

photoperiod 16h, photoperiod extended and light supplemented if necessary (lamp PHILIPS MGR400 with light bulb HPIT 400Watt, white light), 20°C day and night, soil as in GH1, plants were watered daily and fertilized weekly with either Wuxal Super 8-8-6 or Wuxal Top K 21%.

GH3:

photoperiod 16h, photoperiod extended and light supplemented if necessary (lamps by Philips, Germany, MGR400 SONT 400W (yellow) and MGR400 HPIT 400 (white), fertilized daily with Kristalon Scarlet 7.5 + 12 + 36 + 4.5 stock solution 12.8 kg/100 L and Calcinit 15%N, 26% CAO stock solution 9.3kg/100L, mixed 50:50 and diluted to 1.2 µS).

V1:

room by Viesmann, Allendorf, Germany, photoperiod 8h (4x Regiolux lamps for wet rooms, EVG 1x58 W, with Osram 18W/840 in the center of the room, 4x Ludwig lamps for wet rooms, EVG Dim, with Osram 58W,840, +3/4 °C during day and night, plants were not fertilized and watered only when necessary

V2:

vernalization room Fitotron by Weiss Technik, UK, light source Philips GreenPower LED production module deep red/white 120 LO), +5°C during day and night, plants were not fertilized and watered only when necessary

Alignment and phylogenetic reconstruction. Electropherograms of the obtained sequences were controlled for quality using the program SeqMan (DNA Star, Lasergene).

Both chloroplast markers and if necessary both parts of the *ADH* fragment were combined prior to aligning and were analyzed as a unit. Sequences of the six utilized markers (ITS, *trn*L - *trn*L-F, *CHS*, *ADH* and orthologue of *At2g13360*) were aligned with ClustalX (Thompson et al. 1997) and subsequently adjusted by hand. For the markers *CHS*, *ADH* and orthologue of *At2g13360* only the exons of the sequences were employed for the analyses. Bayesian MCMC analyses (Yang and Rannala 1997) were performed with the mpi (message parsing interface) version of MrBayes v.3.1.2 (Ronquist and Huelsenbeck 2003). At first all six markers were run independently. Then a Shimodaira-Hasegawa test (Shimodaira and Hasegawa 1999) was performed to test for congruency of the respective tree topologies (table S13). The initial number of topologies was very small (n=5), what might have biased the levels of significance. Therefore 100 random topologies were added to the congruency tests. Following the SH-Test the alignments of all markers were combined into one

dataset. The corresponding annotated alignment is given in table S3. The combined dataset was divided into five unlinked partitions, to apply the best fitting evolutionary model to each locus. The respective models were chosen using jModeltest v0.1.1 (Posada and Crandall 1998), relying on the Akaike information criterion (AIC).

In the Bayesian analysis based on the combined dataset four simultaneous runs with four chains each were run for 1 million generations. In each run 1001 trees was sampled. When computing the consensus tree (50% Majority Rule) the first 25% of the trees sampled were discarded (burn-in = 250). Completion of the Bayesian runs was determined using the web-based program AWTY (Nylander et al. 2007). The chosen evolutionary models and applied parameters for all Bayesian analyses (combined dataset and single marker datasets) are given in table S4.
For the phylogenetic reconstruction including both co-orthologues of *ADH*, *CHS* and *At2g13360* in a concatenated alignment (introns deleted, alignment length 3163 positions, table S3) a Maximum Parsimony (MP) analysis was performed through the program MEGA (Tamura et al. 2011). A consensus tree was calculated from the two most parsimonious trees. Branches with less than 50% bootstrap support (1,000 replicates) were collapsed. The Close-Neighbour-Interchange method was used for search (Nei and Kumar 2000), 10 initial trees (random addition of sequences) were generated and the MP search level determining the extensiveness of the MP search was set to 3. The tree was calculated as unrooted but displayed as rooted using *Pseudoturritis turrita* as outgroup.
All sequences used for phylogenetic analysis were submitted to genebank. The respective accession numbers are given in the accession table (table S1).

Life cycle of *A. montbretiana* under controlled conditions. The life history of *A. montbretiana* is of particular interest because it is a sister species of the model perennial *A. alpina* (reference accession Pajares, hereafter referred to as *A. alpina* acc. Pajares). Previously *A. montbretiana* was described as an annual plant (Mutlu 2004), however the life cycle was not described in detail under controlled conditions. Therefore growth and development of *A. montbretiana* were closely monitored under greenhouse conditions. After germination the plants grew as a rosette (fig. S12A, B) until bolting when the main shoot elongated and the plants started to produce flowers (fig. S12C). Upon flowering, axillary shoots started to grow and flower in a basipetal direction (fig. S12C and D). At the end of the flowering period during seed maturation *A. montbretiana* underwent generalized senescence causing dehydration and death of the whole plant (fig. S12E). The *A. montbretiana* plants therefore did not survive seed production and were defined as semelparous and annual.

Generation of an *Arabis montbretiana* X *Arabis alpina* F1 hybrid. In order to investigate the flowering behavior of a hybrid of an annual and a perennial plant, *A. montbretiana* flowers were emasculated and cross-pollinated with *A. alpina* acc. Pajares pollen. Since several trials of reciprocal crosses did not yield any viable seed, immature siliques were harvested for embryo culture according to a protocol adapted for *A. thaliana* embryo culture (Sauer and Friml 2008) with some alterations like using *Arabidopsis* GM instead of AM. Only one embryo out of >100 rescued developing seeds started to develop further. It started producing chlorophyll and was transferred to GM medium containing 1% of sucrose. Cultivation with the plate turned upside down and shielded from light was continued until the embryo started developing leaves. Then it was transferred to fresh medium and left to grow in a Petri dish until it had a height of approximately 5 mm. Upon reaching this height it was transplanted to a jar covered with a lid and cultivated first on GM with phytagel, then on autoclaved sand drained with liquid GM and finally on autoclaved soil which was regularly watered with autoclaved water. Eventually the plant was transferred to greenhouse conditions (long day, 16h light). As the plant had reached a suitable size it was propagated by cuttings. Crosses with *A. alpina* as mother did yield any viable seeds at all.

Illumina sequencing of *Arabis montbretiana*, contig assembly, *AmFLC* contig detection and analysis. For *A. montbretiana* an Illumina library with an insert size of 400 bp suitable for paired end

sequencing was generated at the Cologne Center for Genomics/University of Cologne/Germany. 187 million reads with an average length of 113 bp were obtained and subsequently loaded into the CLC Genomics Workbench Version 4.7. Settings were adjusted for library size and a *de novo* assembly was performed. The assembly resulted into ~29,000 contigs and after removing contigs with clear blast hits other than plant and contigs shorter than 200 bp the assembly had a total length of ~199 Mbp (28,775 contigs). The N50 was 21.6 kB and the longest contig had a length of 312 kB. This Whole Genome Shotgun project has been deposited at DDBJ/EMBL/GenBank under the accession LNCH00000000. The version described in this paper is version LNCH01000000. The resulting contigs were indexed as blast library which was then searched for the contig/contigs containing *FLC*. Depending on the analysis the obtained contig were trimmed to the region covering the *FLC* gene as well as the last exon of the upstream gene and the downstream sequence orthologous to what has been described as COOLAIR promoter (Swiezewski et al. 2009) in *Arabidopsis*, the sequence orthologous to the *FLC* gene including all exons and introns or only the sequence orthologous to the first intron of *FLC*. Sequences were aligned by GATA (Nix and Eisen 2005) for detecting duplications and rearrangements within the CDS (*A. montbretiana* versus *A. alpina* published in (Wang et al. 2009) and both orthologues against *FLC* (www.arabidopsis.org)), mVISTA (Frazer et al. 2004; Mayor et al. 2000) (*AmFLC* and *FLC* genomic region against *A. alpina* published in (Wang et al. 2009) covering partial (orthologue of) *At5g10150*, all intergenic sequence between (the orthologue of) *At5g10150* and (the orthologue of) *FLC* and the *COOLAIR* promoter) or YASS (Noe and Kucherov 2005) (*AmFLC* genomic region and *AmFLC* cDNA). A diagram showing the structure of *AaPEP1*, *AmFLC* and *FLC* was generated manually using Adobe Illustrator. Percent similarity of DNA sequences was calculated by ClustalW (Goujon et al. 2010; Larkin et al. 2007; McWilliam et al. 2013). For monitoring the changes in SNP distribution among *AmFLC* and *PEP1* across the locus *AmFLC* and *PEP1* including all exons and introns were re-aligned by mVISTA and the resulting alignment was adjusted manually. SNPs between *AmFLC* and *PEP1* were called. A sliding window (window size 200 bp, shift of 20 bp) was applied and the resulting data were plotted.

Details *PEP1* duplication. In *A. alpina* acc. Pajares the duplicated segments can be differentiated by a 416 bp sequence that is only present in the copy of intron 1 adjacent to exon 1A. This 416 bp is also found in *A. thaliana FLC*. Furthermore, the *A. alpina* duplicated segments of intron 1 are differentiated by 4 SNPs of which 3 are located in the sequence 3' of the 416 bp segment while one is located just upstream of the 416 bp segment. The sequence alignments revealed that the first 950 bp of intron 1 in *A. montbretiana* contain the 416 bp sequence characteristic of intron 1A of *PEP1* (fig. S5D) and all 4 SNPs indicative of intron copy 1B (fig. S13). This combination suggests that *AmFLC* corresponds to the ancestral gene structure that contained both the 416 bp sequence and the SNPs characteristic of copy 1B in *PEP1*. In *A. alpina* duplication of the ancestral sequence followed by deletion of the 416 bp in the intron segment adjacent to exon 1B and 4 point mutations in the intron segment next to intron 1A produced the derived *PEP1* structure present in *A. alpina* acc. Pajares (fig. S5D).

Illumina sequencing of *Arabis nordmanniana*, contig assembly, *AnFLC* contig detection and analysis and estimation of genome size and ploidy level. The genome of *A. nordmanniana* was sequenced by Illumina sequencing of a 400 bp insert size library. Potential adaptor sequences were removed from the raw sequencing reads (~415 million reads) using Cutadapt (Martin 2011). The reads were further trimmed, by removing LEADING and TRAILING nucleotides with quality scores below 15 using Trimmomatic (Bolger et al. 2014). All reads shorter than 50 nucleotides were discarded. ~377 million out of the ~390 million cleaned reads matched in the *de novo* assembly of the *A. nordmanniana* genome and ~300 million of these reads could be assembled in pairs using the CLC Genomics Workbench Version 8.5. The total length of the assembly was ~342 Mbp (267228) after removal of contigs yielding blast hits other than plant or that were shorter than 200bp. The N50 was 4.9 kB and the longest contig had a length of 476 kB.
This Whole Genome Shotgun project has been deposited at DDBJ/EMBL/GenBank under the accession LNCG00000000. The version described in this paper is version LNCG01000000. A blast

database was generated from the contigs using the CLC Genomics Workbench Version 8.5. Searching the blast database generated from the contig assembly resulted in several blast hits to *FLC* or flanking genes. One contig contained the orthologue of *At5g10150* as well as the partial (exon 1 until partial intron 6) *A. nordmanniana* orthologue of *FLC* (hereafter referred to as *AnFLC A*). A second contig could be identified as containing the remaining part of intron 6 as well as exon 6 and some sequence downstream of *AnFLC A*. Amplification of a fragment spanning exon 2 to 7 and sequencing of the end of the fragments confirmed that both contigs can be joined manually. Further analysis of the results of the blast search revealed a third contig that covered the orthologue of *At5g10150*, exon 1, intron 1, exon 2 and parts of intron 2 of the orthologue of *FLC*. Three more contigs were found to make up the remaining part (rest of intron 2, exon 3 to exon 7) of *AnFLC*. Several PCR reactions and subsequent sequencing of the PCR products were used to confirm that these contigs can be used to form a scaffold. Furthermore all four contigs were found to have overlaps (fig. S15) which may result from heterozygosity. For simplicity in the display of the complete alleles these overlaps were removed. Together these contigs make up a second copy of the orthologue of *FLC* hereafter referred to as *AnFLC B*. Aligning the manually scaffolded contigs to *PEP1* and *AmFLC* showed that also in *A. nordmanniana FLC* is encoded by a single gene with 7 exons and 6 introns (alignment in table S14). Interestingly the duplication of the COLDAIR region was only found in *AnFLC A* and not in *AnFLC B*. However, it cannot be concluded if COLDAIR-like 1 (the duplicated copy) was lost in *AnFLC B* or if *AnFLC B* represents the ancestral form of *AnFLC*.
In order to find out if the presence of *AnFLC A* and *AnFLC B* is due to a duplication of *AnFLC* or the result of a whole genome duplication the genome size was estimated from the kmer-frequency distribution using a previously described method (Liu et al. 2014). In brief, the genome size can be estimated by the dividing the total number of kmers in the sequencing data by the expected kmer depth.

The expected kmer depth was obtained by first determining the abundance of individual kmers from the cleaned sequence reads using JELLYFISH (Marçais and Kingsford 2011) and then inspecting the kmer-abundance distribution (fig. S14). In order to limit the influence from low frequency kmers that are likely correspond to sequencing errors we removed all k-mers with an abundance ≤ 4. The total number of kmers in the cleaned sequence reads was 30,676,346,003 and the expected kmer-depth was found to be 44 (fig. S14). Based on these numbers the genome size was estimated to be around 697.18 Mb.

The *A. nordmanniana* assembly was annotated using the MAKER pipeline (Cantarel et al. 2008). As no transcriptomic sequence information was available, the genome was annotated using protein sequences from the following species downloaded from the Phytozome v10 (http://phytozome.jgi.doe.gov/pz/portal.html): *A. thaliana, A. lyrata, Capsella rubella, Capsella grandiflora, Brassica rapa* and *Eutrema salseginium*. In addition to these species, we also used proteins from *A. alpina* and *A. montbretiana*. The initial protein coding genes predicted by MAKER were filtered by performing BlastP (Altschul et al. 1997) searches (e-value threshold $1e^{-5}$) with the encoded proteins against the same protein database used during the annotation. Blast hits were only considered as valid if at least 60% of amino acids of the subject protein were represented in the alignment. *A. nordmanniana* genes without any significant hit were discarded.

Using this workflow we annotated a total of 41,610 protein-coding genes in *A. nordmanniana*. In order to determine the completeness of the predicted protein coding gene space, we searched the predicted proteins against a set of core-eukaryote genes (Parra et al. 2007) using BlastP (e-value threshold: $1e^{-20}$). Significant hits were found against 437 (˜95.4%) of the 458 *A. thaliana* proteins in the core-eukaryote gene set. This analysis suggests that the *A. nordmanniana* assembly covers a very large fraction of its gene space. The predicted proteomes of *A.nordmanniana*, *A.alpina* and *A.montbretiana* (personal communication) were clustered into 20840 potential gene-families using OrthoMCL (Li et al. 2003). Next, we searched groups in which the three species were represented in a 1:1:2 ratio. These groups were assumed to correspond to species-specific duplication events. In total, 4737 groups

harbouring species-specific duplication were identified. The fraction of groups containing *A. alpina* specific duplications (145: ˜3.0%) was similar to the fraction of groups containing *A. montbretiana* specific duplications (160: ˜3.4%). The number of groups with *A. nordmanniana* specific duplications was substantially higher (4432: ˜93.6%). The genome size (approximately double the genome size of *A. alpina* as well as the high abundance of duplicated genes in *A. nordmanniana* make it likely that our accession is a tetraploid individual and that whole genome duplication rather than a single gene duplication is the reason for the presence of two copies of *AnFLC*.

Amplification and sequencing of marker sequences. Four nuclear and two plastidic marker sequences were amplified and sequenced for use in phylogenetic reconstruction. Orthologues of the *A. thaliana* gene *At2g13360* had been used successfully for phylogenetic reconstruction in the Brassicaceae (Duarte et al. 2010). In order to design primers specifically amplifying the locus in the Arabideae (and *Pseudoturritis turrita* as outgroup) the orthologue of *At2g13360* was pulled out of the genome assembly of *A. alpina* acc. Pajares (Willing et al. 2015). Synteny of the respective genomic regions of *A. thaliana* and *A. alpina* was tested using the program GATA (Nix and Eisen 2005). When syntenie was confirmed primers were designed using the program primer3 (Rozen and Skaletsky 2000). Primer sequences, including the ones for the amplification of *ITS*, *trnL*, the *trnL-F* IGS, *CHS* and *ADH* are given in table S2. Marker sequences were amplified by PCR and sequenced directly or after cloning by Sanger sequencing. Details are given in supplementary methods and results.

The PCR mix contained 20-40 ng template DNA, 10 pmol of each primer, 2.5 nmol of each nucleotide, 1x My Budget or GoTaq PCR reaction buffer, 0.1 µl My Budget or Mango Taq polymerase and a final concentration of 2.5 mM $MgCl_2$. Amplification followed a standard 3-step PCR protocol [2' initial denaturation at 95°C, 30 cycles of 30'' denaturation at 95°C, 30'' annealing at a primer pair specific temperature and 1' elongation at 72°C, followed by a final elongation step at 72°C for 5'].

Quality of PCR products was controlled by agarose gel electrophoresis. PCR products were purified using the Macherey & Nagel PCR Purification Kit according according to the manufacturer's protocol or the Promega Wizard SV Gel and PCR Clean-Up Kit prior to cloning (*chs* and *adh* fragments cloned into chemical competent *E. coli* strain JM109 using the pGEM-T vector system (Promega)) followed by Sanger sequencing using the universal T7 and SP6 primers or direct Sanger sequencing using the same primers as for the PCR reaction (forward and reverse) or the universal primers M13FP [5'-TGTAAAACGACGGCCAGT-3'] and M13RP [5'-CAGGAAACAGCTATGACC-3'].

SUPPLEMENTARY FIGURES AND TABLES

Table S1 Details of accessions used in this study stating country of origin, provider of seeds and sequences used in the phylogenetic analysis and their genebank accession numbers; for the pairs of grey shaded accessions marker sequences were used in combination to obtain a full set for the phylogenetic reconstructions

Accession number (Karl and Koch, 2013)	Taxon	author	*ITS*	*trnL*	*trnF*	*ADH*	*CHS*	*360*	State	Seed Number or Donator	number herbarium voucher HEID/KUN/B
	Arabis alpina	L.	KU321355	0	0	KC814706	JQ919880	KU321519	Spain: Pajares	A. alpina Pajares accession (as in Wang et al. 2009)	
RK001	*Arabis alpina*	L.		GU181934	GU181999				ex cult. BG Heidelberg; originally: Slowakia: Carpathian Mountains	from cutting	HEID-809695/6
A-Kili	*Arabis alpina*	L.	JQ919835	JQ919813	JQ919857	KC814698	JQ919879	KU321520	ex cult. BG Heidelberg; originally: Tanzania, Kilimanjaro	plant vegetatively propagated	
RK041	*Arabis aucheri*	Boiss.	HM046191	HM046243	HM046216	KC814699	JQ919881	0	Türkey: B7 Elazig: 38th km from Sivrice to Maden, 1232 m, N 38.28.613 E 39.35.363, 28/5/2002, B.Mutlu & A.A.Dönmez	provided by Birol Mutlu	
RK018	*Arabis auriculata*	Lam.	GU182084	GU181952	GU182017	KC814700	JQ919882	KU321517	ex cult. BG Heidelberg; originally: Italy: S. Pio delle Camere (AQ), 830mNN.	B-2006-0556	HEID-809771/2
	Arabis collina	Ten.	KU321356	0	0	KC814707	JQ919884	KU321515	Italy: Gole Raiano (AQ) 389mNN.	B-2006-0564	
JR045	*Arabis collina*	Ten.		HQ646785	HQ646724				ex cult. BG Heidelberg; originally: Italy: SirenteVelino, Campo Velice, Fosso Ferruccio, 1300mNN	B-2005-0837	HEID-809760/1
JR038	*Arabis hirsuta*	(L.) Scop.	FJ187937	FJ188234	FJ188083	KC814701	JQ919885	KU321514	ex cult. BG Heidelberg; originally: France: St. Martin-sur-Armancon, Yonne (89)	B-2005-0581/B2009-0104	HEID-809750/1
RK043	*Arabis montbretiana*	Boiss.	HM046193	HM046245	HM046218	KC814708	JQ919886	KU321516	ex cult. BG Heidelberg; originally: Turkey; C10 Hakkari: Between Şemdinli - Şapatan passageway,2. km from Şemdinli, rocky slopes, N: 37.19.70.8-E: 44.33.50.8	provided by Birol Mutlu (BM7968)	HEID-809801

Accession number (Karl and Koch, 2013)	Taxon	author	*ITS*	*trnL*	*trnF*	*ADH*	*CHS*	360	State	Seed Number or Donator	number herbarium voucher HEID/KUN/B
RK219	*Arabis nordmanniana*	Rupr.	KF547353	KF547826	KF547624	KC814702	JQ919887	KU321518	ex cult. BG Heidelberg	B-2006-0170	HEID-809792/3
	Arabis nova ssp. iberica		KU321353	KU321361	KU321359	KU525033	KU525031	KU321504	Spain: Madrid, San Agustín de Guadalix, path besides Guadalix river, next to Isabel II channel facilities; P. Jiménez Mejías & J.E. Rodríguez, 18PJM13, 28.04.2013	provided by Dr. Santiago Martín-Bravo	HEID-504744-58
RK127	*Arabis nova ssp. nova*	Vill.	JQ919839	JQ919817	JQ919861	KC814709	JQ919888	KU321506	Slowakia: centralis Velka Fatra in declivibus petrosis in fundo valis Dedosova, australis.		B10 0201566
	Arabis purpurea	Sm.	KU321357	0	0	KC814703	JQ919889	KU321509	Cyprus: along river, between Millomeri and village, 30.03.2009	provided by Maarten Koorneeff	
RK004	*Arabis purpurea*	Sm.		GU181938	GU182003				ex cult. BG Heidelberg; originally: Cyprus: slopes of Kalopanaiotis, Olymbos Troodos.	B-2006-1043	HEID-809699/700
RK025	*Arabis setosifolia*	Al-Shehbaz	GU182089	GU181957	GU182022	KC814713	JQ919899	KU321508	China: Xizang (Tibet), Gongjue (Gongjo) Xian, ca. 8.6 km in a straight line NW of city of Gongjue; N 30°54'25", E 98°11'37"		KUN
RK212	*Arabis verna*	(L.) R. Br.	JQ919840	JQ919818	JQ919862	KC814704	JQ919890	KU321513	ex cult. BG Heidelberg; originally: Spain, S-Spain, calcareous soil, near Ronda, Málaga.	B-2006-1039	HEID-809837/8
RK214	*Aubrieta canescens ssp. macrostyla*	Cullen & Hub.	HQ646642	HQ646763	HQ646702	KC814705	JQ919891	KU321510	ex cult. BG Heidelberg; originally: Turkey, NOTurkey, Prov. Sivas.	B-2010-0002	HEID-501009; HEID-504636
Dra0002	*Draba aizoides*	L.	KF547181	GU202868	GU202800	KC814710	JQ919893	KU321505	ex cult. BG Heidelberg; originally: Slowakia: Carpathian Mountains; Okres Poprad: Tatranská Lomnica, Botanical Garten		HEID-404443
Dra0026	*Draba hispanica*	Boiss.	JQ919844	JQ919822	JQ919866	KC814711	JQ919894	KU321512	ex cult. BG Heidelberg; originally: Morocco: HighAtlas, Jbel Toubkal, 2200mNN	B-2006-0510	
	Draba nemorosa	L.	KU321354	KU321360	KU321358	KU525032	KU525030	KU321511	ex cult. BG Heidelberg; originally: Turkey: Prov. Artvin, Kackar-Mountains, 2100mNN (Tolimir, 1714)	B-2002-0157	

Accession number (Karl and Koch, 2013)	Taxon	author	*ITS*	*trnL*	*trnF*	*ADH*	*CHS*	*360*	State	Seed Number or Donator	number herbarium voucher HEID/KUN/B
RK237	*Pseudoturritis turrita*	(L.) Al-Shehbaz	JQ919845	JQ919823	JQ919867	KC814712	JQ919896	KU321507	ex cult. BG Heidelberg; originally: Italy, Monte Cetona/Cetona (Siena), bordo bosco (950 m s.l.m.)	B-2008-0093	HEID-809857/8

Table S2 Primers used in this study for amplification of phylogenetic marker sequences, qPCR and amplification of the mRNA of FLC orthologues as well as the genomic loci

**marker**	**forward**	**reverse**	**published by**
At2g13360	5'-GCAACACCACTTCCCATTACA-3'	5'-AACCGGAACAACGAGGATTA-3'	
ITS	5'-GGAAGGAGAAGTCGTAACAAGG-3'	5'-TCCTCCGCTTATTGATATGC-3'	(White et al. 1990), (Mummenhoff et al. 1997)
trnL	5'-CGAAATCGGTAGACGCTACG-3'	5'-GGGGATAGAGGGACTTGAAC-3'	(Taberlet et al. 1991)
trnL-F	5'-GGTTCAAGTCCCTCTATCATCCC-3'	5'-GATTTTCAGTCCTCTGCTCTAC-3'	(Taberlet et al. 1991), (Dobeš et al. 2004)
CHS	5'-CATCTGCCCGTCCATCAAACCTACC-3'	5'-TTAGAGAGGAACGCTCTGCAAGAC-3'	(Koch et al. 2000)
CHS (orthologue specific)	5'-GGCACARAGAGCTGATGGA-3'	5'-AGAGAAGATGAGAGCRACWCG-3'	
ADH	5'-ACTACTGGTCAGATTATTCGATGC-3'	5'-AAGCACCCATGGTAATGATGC-3'	(Koch et al. 2000)
ADH (alternative reverse primer)		5'-CTAACCCAGTAGATAAACCACAAC-3'	(Koch et al. 2000)
ADH (alternative forward primer)	5'-GTTAGTTGTGGTTTATCTACTGG-3'		(Koch et al. 2000)
ADH REV-5		5'-GCAACACTTTGACCTTTCTT-3'	(Koch et al. 2000)
ADH REV-6		5'-CATTCAAATGCTTGAATCATAGC-3'	(Koch et al. 2000)
PP2A (qPCR) *A. alpina*	5'-AGTATCGCTTCTCGCTCCAG-3'	5'-AACCGGTTGGTCGACTATTG-3'	(Bergonzi et al. 2013)
RAN3 (qPCR)	5'-CACAGGAAAAACCACATTCGT-3'	5'-CCATCCCTAAGACCACCAAAT-3'	(Wang et al. 2009)
AnRAN3 (qPCR)	5'-GTGTACAGGAAAAAGGAGATTTGT-3'(for1) 5'-GTGGACAGGAAAAACTACATTCGT-3'(for2)	5'-ATCCATCCCTAAGACCACCAAATTTC-3'	
AnFLCA (qPCR)	5'-CTTGTCGTCTCCGCCTCCGG-3'	5'-TCCAGCTGAACGAGGGTATCGAC-3'	
AnFLCB (qPCR)	5'-CTCGTCATCTCCGCCTCCGG-3'	5'-TCCAGCTTAACGAGGGAATCGTT-3'	
AauFLC (qPCR)	5'-CTCGTCGTCTCCGCCTCCGG-3'	5'-AGAGGGCAGTTTCAAGGTGTTCC-3'	
AmFLC (qPCR)	5'-CTTGTCGTCTCCGCCTCTGG-3'	5'-AGAGAGCAGTTTCAAGGTGTTCC-3'	
PEP1(qPCR)	5'-CTTGTCGTCTCCTCCTCTGG-3'	5'-AGAGAGCAGTTTCAAGGTGTTTT-3'	
AauPP2A(qPCR)	5'-AGTATCTCTTCTCGCTCC AG-3'	5'-AACCGGTTGCTCAACTATTG-3'	
amplification of intron 1 to exon 2	5'-ACTCTACAGCTTCTCCTCCGGTGAT-3'	5'-CCATATCGATCAAGGATCTTGACCA-3'	Renhou Wang, unpublished
amplification of exon 2 to exon 7	5'-CTGGTCAAGATCCTTGATCG-3'	5'-GCTTAATTGAGTAGTGGGAGAG-3'	Maria Albani, unpublished
amplification of intergenic region between orthologue of At5g10150 and FLC orthologue	5'-AATGTCATCATCGAAGCAAATC-3'	5'-ACATCAATTATGCATCACAACG-3'	forward designed for this study, revers Maria Albani, unpublished
sequencing cDNA	5'-GCCGACAAGTCACCTTCTC-3'	5'-AGCTTCTGCTCCCACAAGAG-3'	Maria Albani, unpublished

Table S3 (separate file Table_S3.txt) Alignment of marker sequences used for phylogenetic analysis; the alignment is given in nexus format and partitions are indicated at the end of the file

Table S4 Results of Model Testing and Bayesian analyses for the individual single marker dataset and the combined dataset

	orthologue of At2g13360 dataset	adh dataset	chs dataset	ITS dataset	*trnLF dataset*	combined dataset
chosen evolutionary model (AIC)	K80+Γ	TrN+Γ	TIM2ef+Γ	SYM+Γ	*TPM1uf+Γ*	5 partitions with the respective evolutionary models for each marker
No. of generations	2 million	2 million	2 million	15 million	*30 million*	2 million
No. of taxa	17	18	18	18	*18*	18
No. of characters	915	1087	1161	632	*756*	4551
Ln likelihood [harmonic mean]	-2729.38	-4187.91	-4214.25	-2496.21	*-2006.07*	-15768.96
Average standard deviation of split frequencies	0.0074	0.0080	0.0045	0.0092	*0.0115*	0.0016

Table S5 (separate file Table_S5.txt) Alignment of marker sequences (ADH, CHS, *At2g13360*) used for the maximum parsimony analysis including both co-orthologues of *A. nordmanniana* for each marker. Introns were not included in the analysis and therefore deleted

Table S6 Vernalization response of selected Arabideae; plants were grown either in long day for several months or in long day for 6 weeks, transferred into vernalization for 12 weeks and then returned to warm temperatures and long day conditions. Leaf number (TLN) at the main shoot was determined at the time of flowering; plants responding to vernalization should flower with less leaves when vernalized (facultative vernalization requirement) or only after vernalization (obligate vernalization requirement)

**Taxon**	**Country**	**Vernalization response**	**Vernalization requirement obligate**	**Leaf number flowering without vernalization**	**Leaf number flowering after vernalization**
Arabis alpina L.	Spain	yes	yes	-	Wang et al. 2009
Arabis auriculata Lam.	Italy	yes	no	70.3 +/- 3.5	38 +/- 1.4
Arabis montbretiana Boiss.	Turkey	yes	no	48.2 +/- 2.9	23.6 +/- 1
Arabis nova ssp. *iberica*	Spain	yes	no	74.2 +/- 11.1	38 +/- 1.9
Arabis nordmanniana Rupr.		yes	yes	-	-
Arabis purpurea	Cyprus	no	-	-	-
Arabis aucheri	Tuerkey	yes	yes	-	too complex for leaf count

Table S7 Polymorphic sites in *Arabis alpina* Pajares and *Arabis montbretiana* and the polymorphisms found in the hybrid individual in the ITS confirming the hybrid identity of the plant. Alignment positions in the ITS alignment are given above the SNPs, for the hybrid the ambiguous code is given at each SNP position

alignment position	56	83	137	199	203	211	216	225	226
Sanger read in Hybrid	Y	Y	Y	W	K	R	Y	K	W
Sanger read in *A. alpina*	T	C	T	T	G	A	C	T	T
Sanger read in *A. montbretiana*	C	T	C	A	T	G	T	G	A

Table S8 Primers used for genotyping the F1 plant and introgression lines carrying a genomic fragment including *AmFLC* . Markers after the black bar in the Table were used to genotype the introgression lines and are numbered as in fig. S5. The table states marker position in *A. alpina*, length of the amplified fragment in *A. alpina* and *A. montbretiana* as well as the primer sequence. In case of the F1 plant the resulting genotype is given and all were heterozygous.

no.	*A. alpina* chromosome	position in Mbp in *A. alpina*	*A. alpina* fragment length (bp)	*A. montbretiana* fragment length (bp)	*A. montbretiana x A. alpina*	primer sequence (F: forward, R: reverse)
-	1	14,4	518	574	heterozygous	F: TGACACGTAAGCTCAAAATGG<br>R: ATCCCGAAAGAAACACTCTGC
-	1	25,2	813	878	heterozygous	F: CTTCCGGACTAACGCACC<br>R: CAGAAGATTATATTGTTCGAAACCC
-	1	3,1	146	210	heterozygous	F: GGAGGAAGCAGAAGGAAACTC<br>R: GAGGCGCAAAACTCCTTGAG
-	2	0,8	533	600	heterozygous	F: ACTGAGTGAGTGAGTGAGTG<br>R: GTGTGTGTGTGTTTTTGCTC
-	2	7,3	359	294	heterozygous	F: GCAGTGTCACACTTCTCACG<br>R: GGGAGACGAAATAAACAAATCAGC
-	2	14,5	175	248	heterozygous	F: TGTAGGACCTTGTAAAGATGAAGG<br>R: ACGAAAGGTTGCAACATTATCC
-	2	20,2	110	180	heterozygous	F: GGGTTAACTGTGGCTGCTAGG<br>R: CCTGAGAAGGAGCGGCATATC
-	2	25,8	551	500	heterozygous	F: CCCAGCTCTCGCTGTCCAAGC<br>R: CCCGGGCACTGTTGAGAGGT
-	2	28,1	422	368	heterozygous	F: AATGGTGCAAAGACGGAAG<br>R: CTCGTAATTTGGTAAAAGCC
-	3	3,07	293	367	heterozygous	F: GGTCCTCATCTTGATGCTCG<br>R: CGTTGGATTTTGACTGCGATG
-	3	10	481	417	heterozygous	F: GTTCAACAAGACTATTCTGC<br>R: TACGTACAATTTGGACCGTCG
-	3	27,4	504	422	heterozygous	F: GCCATTTCAAGCGCTTCCTC<br>R: CCACATCACTACTCAAAAATACAC
-	4	0,07	794	849	heterozygous	F: CTCAAATCGGGCTTACACAGG<br>R: CATCTCTTGCATGAAATCGC
-	4	4	374	300	heterozygous	F: CGTTGCTCTACACTCGTTCC<br>R: GTCTCCACCTTTGTGAATTG
-	4	9	519	592	heterozygous	F: GCTTCTTCTATGTTGTTTCATGTGC<br>R: GTTCATACCCACCGGTGATGG
-	4	14	290	328	heterozygous	F: CGAGGAACCATATTGCCACCAATC<br>R: GATTTAGGATCCTACTGATCGGGA ATG
-	4	18,1	179	153	heterozygous	F: TTAAATTGTACATTATTAAATTC<br>R: TTCATGGTTTGGTTAGTGTG
-	4	23,15	794	884	heterozygous	F: GCAATTGAAAGACCGACGCAT<br>R: TGCTCATCAAACTTGTGCTTGT
-	5	0,01	533	482	heterozygous	F: CTGGGACTTTGCGGCTTCTGC<br>R: GCGGCTGGCCTGAGTGTGAG
-	5	3,6	177	248	heterozygous	F: CATTGAATGACTCGTAGCCGC<br>R: ACCAGACTGAAACTTGGATAAACC
-	5	10,9	392	452	heterozygous	F: AGGGTCGACTTAACAGCATCC<br>R: TTATAGGAGACACACGTGGCG
-	5	14,4	243	294	heterozygous	F: ACTCGAGAGTTAAGTCCACTCG<br>R: AAACCTGAAATCCGAGACTACC
-	5	18	189	248	heterozygous	F: TCAAGAGTGGTTAACTTGACAGC<br>R: TGAGATTTTATGTGTGGCAGCC
-	5	22,6	824	892	heterozygous	F: GGCCTACGATAATCTCATTTCCC<br>R: TTGGCTAACTACGCCTGTTCG
-	6	0,23	296	215	heterozygous	F: CATCCTTAGCCTTCTCAACCC<br>R: TTCGTCTCCGGTGTTTTCTGG
-	6	6,7	353	407	heterozygous	F: GTTTTCAATCCGTCAATTCGAC<br>R: CGTGTTTACCTTTTGTGTGCT
-	6	12,2	531	600	heterozygous	F: GATGGCGTTACTCCTTTTCAC<br>R: TTGGTAAGCAAAAGGTCACGG
-	6	25,2	207	286	heterozygous	F: GGTTTAGCAGATTTCAGGTGG<br>R: AGTTTATTAAGAAAAGAGCTGAGC
-	6	32,9	710	616	heterozygous	F: GTCGTTCTATGGGTGGTCCTC<br>R: GTGCAGGCAATTTTGTGATC

-	7	0,08	311	343	heterozygous	F: CATATATACATTATTAATAACTTAC<br>R: TTGATTTCTATTTATCAATGTAC
-	7	10	736	798	heterozygous	F: CAGGGATCAACCGAAACTCC<br>R: ATGGAAGGAGACAGGTCCAG
-	7	14,5	667	742	heterozygous	F: CGAGAGGCGCATCGTTCC<br>R: AACGTTCGCCAGCATATTCCC
-	7	18,1	188	266	heterozygous	F: GTTCGTATCGGTTTCCCCTCC<br>R: CCACATCACCCTGGTGTATGG
-	7	26,2	241	187	heterozygous	F: GGATTCACCGGAATTTCACCG<br>R: GCTCGATGTTCCAGTGAGTC
-	7	28,8	877	793	heterozygous	F: CTCAGAGTTCCATCTGCGCTG<br>R: GTGGTTGATGTGAAAGAGGCG
-	8	0,05	638	705	heterozygous	F: GATCTTGTTCTAATTGCTGTGC<br>R: GACCCACGATCAACAATTTACC
-	8	7	473	528	heterozygous	F: GTCGTCTTCTCGATTCTTCTTCC<br>R: CTGTACAACCCTAGTTTGCTGC
-	8	4	533	584	heterozygous	F: TATCCTCTTCTCACCATGCGC<br>R: AAAGAAGACATCGAGGCGAGC
-	8	21,08	897	822	heterozygous	F: GTTTGTGCATTAGAAAGCGC<br>R: GTCAGTAAAATGGAAGCTCC
-	8	35,14	886	812	heterozygous	F: CTCCGTACTCCGATCAATTAAACC<br>R: GATTCCCCGAAGTAACAGCC
1	1	0,1	553	631		F: GAAGAAGATGCAGCAGTCTCCAC<br>R: GGTGGTGCAGCTCCTGGTGT
2	1	3,1	146	210		F: GGAGGAAGCAGAAGGAAACTC<br>R: GAGGCGCAAAACTCCTTGAG
3	1	7,2	797	859		F: TTCCCTAACACAGAGCTGG<br>R: GTAGAACGCGCTTCCTTCC
4	1	10	470	406		F: ATCCGCCTACATTTCCACGAC<br>R: GAGTTCACCGCTATTCGCAAG
5	1	14,4	518	574		F: TGACACGTAAGCTCAAAATGG<br>R: ATCCCGAAAGAAACACTCTGC
6	1	18	820	886		F: TCATCTCTTTGATCTGCCGGG<br>R: TTCCCTGCTCCAATCCAAACC
7	1	20,27	781	855		F: ACACTCGGCCCTGTTTTCAC<br>R: CAACGATTATTGCAAACGAAAC
8	1	25,2	813	878		F: CTTCCGGACTAACGCACC<br>R: CAGAAGATTATATTGTTCGAAACCC
9	2	0,8	600	533		F: ACTGAGTGAGTGAGTGAGTG<br>R: GTGTGTGTGTGTTTTTGCTC
10	2	7,3	359	294		F: GCAGTGTCACACTTCTCACG<br>R: GGGAGACGAAATAAACAAATCAGC
11	2	9,1	251	348		F: GCATACTTGAGGATGCTCATG<br>R: ACACAACCCAAACTTGTCCT
12	2	14,5	175	248		F: TGTAGGACCTTGTAAAGATGAAGG<br>R: ACGAAAGGTTGCAACATTATCC
13	2	20,2	180	110		F: GGGTTAACTGTGGCTGCTAGG<br>R: CCTGAGAAGGAGCGGCATATC
14	2	25,8	551	500		F: CCCAGCTCTCGCTGTCCAAGC<br>R: CCCGGGCACTGTTGAGAGGT
15	2	27,4	208	278		F: CACACGACGAGGAAGTGTTT<br>R: CCTGTCCATCAGCAACGATC
16	2	28,1	422	368		F: AATGGTGCAAAGACGGAAG<br>R: CTCGTAATTTGGTAAAAGCC
17	3	0,06	600	529		F: ACTACGTGGCCCATTGAAGT<br>R: TTCCGAGACGACCGATGTAG
18	3	3,07	367	293		F: GGTCCTCATCTTGATGCTCG<br>R: CGTTGGATTTTGACTGCGATG
19	3	7,2	522	439		F: AAAACCCACACAAAAGGTTCG<br>R: TGATCTCTGATTCTTTTGTTCATCG
20	3	10	417	481		F: GTTCAACAAGACTATTCTGC<br>R: TACGTACAATTTGGACCGTCG
21	3	14,4	753	803		F: ATGAGATCATCAGCGTAGACG<br>R: TGAAGGACTTCAATGGTGGGG
22	3	20,5	429	516		F: CGACTACACCAACCACTCGA<br>R: GTCACCACGTGATCAGATGG
23	3	27,4	422	504		F: GCCATTTCAAGCGCTTCCTC<br>R: CCACATCACTACTCAAAAATACAC
24	4	0,07	794	849		F: CTCAAATCGGGCTTACACAGG<br>R: CATCTCTTGCATGAAATCGC

25	4	2,15	174	263		F: CATGCCAAATGAATGGTCTC<br>R: GAGAGGCCGTACGAAACAAG
26	4	4	374	300		F: CGTTGCTCTACACTCGTTCC<br>R: GTCTCCACCTTTGTGAATTG
27	4	7,2	527	477		F: TGTTGAGTTTAGGCTAATGTTTTGG<br>R: TCAAGGTCACAAACAGAGAGG
28	4	9	519	592		F: GCTTCTTCTATGTTGTTTCATGTGC<br>R: GTTCATACCCACCGGTGATGG
29	4	14	290	328		F: CGAGGAACCATATTGCCACCAATC<br>R: GATTTAGGATCCTACTGATCGGGAATG
30	4	18,1	179	153		F: TTAAATTGTACATTATTAAATTC<br>R: TTCATGGTTTGGTTAGTGTG
31	4	23,15	794	884		F: GCAATTGAAAGACCGACGCAT<br>R: TGCTCATCAAACTTGTGCTTGT
32	5	0,01	482	533		F: CTGGGACTTTGCGGCTTCTGC<br>R: GCGGCTGGCCTGAGTGTGAG
33	5	3,6	177	248		F: CATTGAATGACTCGTAGCCGC<br>R: ACCAGACTGAAACTTGGATAAACC
34	5	10,9	392	452		F: AGGGTCGACTTAACAGCATCC<br>R: TTATAGGAGACACACGTGGCG
35	5	14,4	243	294		F: ACTCGAGAGTTAAGTCCACTCG<br>R: AAACCTGAAATCCGAGACTACC
36	5	18	189	248		F: TCAAGAGTGGTTAACTTGACAGC<br>R: TGAGATTTTATGTGTGGCAGCC
37	5	21,6	493	549		F: AACCGGCATTTCATATTCCGG<br>R: TCCATGACGGTTCTGCTTCC
38	5	22,6	824	892		F: GGCCTACGATAATCTCATTTCCC<br>R: TTGGCTAACTACGCCTGTTCG
39	6	0,23	215	296		F: CATCCTTAGCCTTCTCAACCC<br>R: TTCGTCTCCGGTGTTTTCTGG
40	6	2,8	532	595		F: GCAAGGTGCAAGATCGTGTT<br>R: CAAGAGAATCATCACGGTGAAGC
41	6	3,9	524	591		F: CTGCCTTGCTACTGTGGG<br>R: CAGGAGCTACATTTCATATTACTCC
42	6	6,7	407	353		F: GTTTTCAATCCGTCAATTCGAC<br>R: CGTGTTTACCTTTTGTGTGCT
43	6	7,2	218	270		F: CTGCTTTTGTTCTGGAATAATAAGC<br>R: GTAATTGCGGTAGCCACAACC
44	6	12,2	600	531		F: GATGGCGTTACTCCTTTTCAC<br>R: TTGGTAAGCAAAAGGTCACGG
45	6	16,3	331	241		F: TAAGGTTGGTGGAATGGGACC<br>R: TGGGTTTCCAAGCTTAGCGAT
46	6	20,5	875	807		F: CCGTTTTCACTCTCGGTTGAC<br>R: GGTTTTACCTTTGTCGATGGC
47	6	21,8	630	541		F: TGCCTCAAAATAGTACAAAAATGC<br>R: AGGATTCCCAGTGATTCGACC
48	6	25,2	207	286		F: GGTTTAGCAGATTTCAGGTGG<br>R: AGTTTATTAAGAAAAGAGCTGAGC
49	6	29	684	626		F: AACAATGCATTGACCTTGAGA<br>R: TCTTCTTACAAGGGCGTGAGA
50	6	32,9	616	710		F: GTCGTTCTATGGGTGGTCCTC<br>R: GTGCAGGCAATTTTGTGATC
51	6	36,9	359	442		F: ACAAAGAAATGACCAAAACTCCCC<br>R: CCGGAATAGTGGCAGATACCG
52	7	0,08	~300	~280		F: CATATATACATTATTAATAACTTAC<br>R: TTGATTTCTATTTATCAATGTAC
53	7	5	339	417		F: GTTACAACATGTGAGTGAAGG<br>R: GTCTTGTGACTTATGAAAATGCCTG
54	7	8,1	182	246		F: GCCCCTCATCACCTATCTCG<br>R: ATAACCGATCCGACCAGTACC
55	7	10	735	798		F: CAGGGATCAACCGAAACTCC<br>R: ATGGAAGGAGACAGGTCCAG
56	7	14,5	667	742		F: CGAGAGGCGCATCGTTCC<br>R: AACGTTCGCCAGCATATTCCC
57	7	18,1	188	266		F: GTTCGTATCGGTTTCCCCTCC<br>R: CCACATCACCCTGGTGTATGG
58	7	20,8	432	495		F: GGCTGCATGCTCATATCCTC<br>R: CCCTTTCAGAGCTATAACACC
59	7	26,2	187	241		F: GGATTCACCGGAATTTCACCG<br>R: GCTCGATGTTCCAGTGAGTC
60	7	28,8	793	877		F: CTCAGAGTTCCATCTGCGCTG

						R: GTGGTTGATGTGAAAGAGGCG
61	8	0,05	705	638		F: GATCTTGTTCTAATTGCTGTGC<br>R: GACCCACGATCAACAATTTACC
62	8	0,5	813	889		F: TTTCGATCCATCATACCCACC<br>R: GGTAAACGTTCTTCTCTATCCGC
63	8	0,6	364	493		F: CAACCTGGCTGACATGTGGC<br>R: CCCATTGGGCCCATATTTCATTC
64	8	1,2	142	196		F: GGTTCAATAGCCGTGAGATGC<br>R: CGGCAATACGAACACTTCAGC
65	8	2	550	606		F: AGCCTACAACATCAAAGTAGC<br>R: TTCTTCCCACAAAAGCCATCC
66	8	2,5	357	455		F: TCATCGTCCAAACAGAGAGCC<br>R: TGGGCTTTAGTTAGATCCGGC
67	8	4	533	584		F: TATCCTCTTCTCACCATGCGC<br>R: AAAGAAGACATCGAGGCGAGC
68	8	5	456	506		F: ACTGATCCCAAGTAACCAAAGC<br>R: ATGGCGTTTTGACCTCTCTCC
69	8	6,1	313	366		F: TCTTCGTTTCAGGTTTTGGG<br>R: AACATATTCCAAAACACTGCACC
70	8	6,5	634	690		F: GATGAACACGAGGAGAGCTGG<br>R: TCAGACAACTTCGTTCACAAATCC
71	8	7	473	528		F: GTCGTCTTCTCGATTCTTCTTCC<br>R: CTGTACAACCCTAGTTTGCTGC
72	8	7,2	209	142		F: GAGGGCTTTTGAATGATGGGC<br>R: CATGTCAACTGAAGGCTCTCG
73	8	10,8	240	180		F: CCTATGTACTCTTCTATGGGACCC<br>R: GCTCTGAGACTCAAAGCATTGC
74	8	15,4	563	619		F: CCTAGAAAATCTTAGAAACAGC<br>R: CCAACCACCAAGATTCTCAC
75	8	18	563	653		F: TGGTGGAGCATGGATTTCTCG<br>R: TCAACGTCCATCCAATCCACC
76	8	21,08	822	822		F: GTTTGTGCATTAGAAAGCGC<br>R: GTCAGTAAAATGGAAGCTCC
77	8	25,3	445	496		F: TGGACACCTTGTTCAAAAGC<br>R: AGGGATGATTCCGACCTTCC
78	8	28,8	600	677		F: TTCTCTGCAATCAAAGCAAAGC<br>R: CAAACCCTTCAAGCCTCAAGC
79	8	35,14	886	812		F: CTCCGTACTCCGATCAATTAAACC<br>R: GATTCCCCGAAGTAACAGCC
80	8	38,5	200	255		F: CAAGCAAACATGTTTTGATAC<br>R: CATCTCCTGGTAGCCGTTCTC

Table S9 Number of SNPs and indel polymorphisms detected in different features of *PEP1* versus *AmFLC*, *Am* = *A. montbretiana*, *Aa* = *A. alpina*, *At* = *A. thaliana* (exons, regulatory sequences known from *FLC* (proximal promoter (Sheldon et al. 2002); nucleation region is the sequence homologous to the region showing elevated H3K27me3 before vernalization in *Arabidopsis* (Yang et al. 2014); *COLDAIR* is the regions homologous to the COLDAIR RNA encoding region (Heo and Sung 2011); Vernalization Responsive Element (VRE) indicates the region homologous to the VRE of *A. thaliana* identified in (Sung et al. 2006))

feature	length of feature in *Aa* and *Am*	SNPs	indels	lenght of indels in order of appearance	% identity *Am* and *Aa*	% identity *Aa* and *At*	% identity *Am* and *At*
full gene (ATG to stop)	Aa: 13,766 Am: 6,186						
proximal promoter	Aa: 251 Am: 261	7	5	12, 1, 1, 1, 1	89.20	86.18	87.50
exon 1	Aa: 185 Am: 185	2	0	none	98.92	92.43	92.43
exon 2	Aa: 57 Am: 57	1	0	none	98.25	89.47	91.23
exon 3	Aa: 73 Am: 73	0	0	none	100.00	89.04	89.04
exon 4	Aa: 95 Am: 95	4	0	none	95.79	78.95	82.11
exon 5	Aa: 42 Am: 42	0	0	none	100.00	88.10	88.10
exon 6	Aa: 42 Am: 42	0	0	none	100.00	95.24	95.24
exon 7	Aa: 108 Am: 108	1	0	none	99.07	65.69	64.71
proximal nucleation region	Aa: 1010 Am: 996	37	9	2, 1, 2, 2, 10, 1, 5, 9, 4	94.22	66.84	67.17
VRE-like 1	Aa: 499 Am: 147	5	4	233, 131, 2, 2	87.76	63.14	60.42
VRE-like 2	Aa: 226 Am: 227	9	1	1	95.58	not present in *At*	not present in *At*
COLDAIR-like 1	Aa: 651 Am: 550	20	8	119, 2, 11, 3, 4, 1, 1, 2	87.06	65.42	63.19
COLDAIR-like 2	Aa: 691 Am: 698	11	4	1, 6, 4, 10	96.78	not present in *At*	not present in *At*

Table S10 (separate file Table_S10.txt) Alignment of the *PEP1*, *AmFLC*, *AlFLC1* and *FLC* loci comprising a partial sequence of the next upstream gene or its orthologues from *A. thaliana* (*At5g10150*), all intergenic sequence between *At5g10150* and its orthologues and *FLC* and orthologues as well as all sequences orthologues/homologous to exons and introns of *FLC* (including indels) and approximately 500 bp of sequence downstream of the stop codon

Table S11 Summary of the comparison of *AtFLC*, *AmFLC*, *AlFLC1* and *AaPEP1*. For each feature (exons, intergenic/intronic segments) position in the respective sequence, length of the feature and % similarity in respect to *A. thaliana* are given. According to their order in the intron sequences were named VRE-like 1 (more 5’ located) and VRE-like 2 (downstream of VRE-like 1) as well as COLDAIR-like 1 (more 5’ located) and COLDAIR-like 2 (downstream of COLDAIR-like 1)

Feature	*A. alpina* position; length; % identity to *At*	*A. montbretiana* Position; length; % identity to *At*	*A. lyrata* position; length; % identity to *At*	*A. thaliana* position; length
partial (orthologue of) *At5g10150*	1 to 74; 74	1 to 68; 74	1 to 56; 56	1 to 56; 56
Conserved sequence stretches 1 = partial (orthologue of) 3’UTR *At5g10150* which aligns to *A. alpina* Pajares (rankVISTA region)	39681 to 39876; 197; 82.53%	123 to 319; 196; 83.23%	105 to 276; 172; 91.36%	112 to 278; 167
duplicated promoter copy A	48083 to 49531; 1446	No duplication	No duplication	No duplication
conserved proximal promoter (until ATG)	49279 to 49531; 274; 85.53	2741 to 3003; 284; 86.61%	1531 to 1789; 288; 91.41%	2628 to 2892; 294
5’ UTR in *Arabidopsis thaliana* (TAIR)	49401 to 49532; 131; 88.89%	2874 to 3003;130; 91.59%	1680 to 1789;110; 92.45%	2784 to 2892; 109
Exon 1 A	49532 to 49716; 185; 92.43%	3004 to 3188; 185; 92.43%	1790 to 1974; 185; 98.38%	2893 to 3077; 185
intron 1, copy A, part I	49717 to 50073; 357	3189 to 3531; 343	1975 to 2329; 354	3078 to 3409; 332
Intron 1, copy A, insertion	50074 to 50489; 416	3532 to 3947; 416	2330 to 2676 (partial); 346	3410 to 3795; 386
intron 1, copy A, part 2	50490 to 50690	not present	not present	3796 to 3983
duplicated promoter, copy B	54604 to 56049; 1446	not present	not present	not present
exon 1 B	56050 to 56234; 185; 92.43%	not present	not present	not present
intron 1, copy B, part 1	56235 to 56783; 548	not present	not present	not present
intron 1, copy B, part 2	56592 to 56792	3948 to 4148	not present	not present
rest intron 1	56793 to 60838; 4247	4149 to 6767; 2627	2677 to 4527; 1851	3984 to 6571; 2596
exon 2	60838 to 6095; 58; 89.66%	6767 to 6824; 58; 91.38%	4527 to 4584; 58; 96.55%	6571 to 6628; 58
exon 3	61447 to 61535; 89; 90.32%	7376 to 7437; 62; 90.32%	4759 to 4820; 62; 91.94%	6807 to 6868; 62
exon 4	61627 to 61725; 99; 80%	7529 to 7627; 99; 83%	4909 to 5008; 99; 90%	6959 to 7057; 99
exon 5	61807 to 61849; 42; 88.10%	7709 to 7751; 42; 88.10%	5086 to 5128; 42; 90.48%	7136 to 7178; 42
exon 6	62064 to 62105; 42; 95.24%	7966 to 8007; 42; 95.24%	5327 to 5368; 42; 97.62%	7373 to 7414; 42
exon 7	63190 to 63297; 108; 88.89%	9082 to 9189; 108; 87.88%	6889 to 6990; 102; 95.10%	8407 to 8508; 102
3' UTR in *Arabidopsis thaliana and A. alpine* Pajares	63298 to 63511; 214; 77.20%	9190 to 9412; 223; 78.64%	6991 to 7214; 224; 95.02%	8509 to 8735; 228
conserved downstream sequence (including homologue of COOLAIR promoter and R-loop	63298 to 63687	9190 to 9580	6991 to 7372	8509 to 8912
*VRE-like 1**	57020 to 57520	4377 to 4523	2691-2831	4221 to 4509
*VRE-like 2**	59426 to 59664	5381 to 5620	2691-2831	4221 to 4509
*COLDAIR-like 1 promoter***	57020 to 57358	4377 to 4482	absent	4221 to 4319
*COLDAIR-like 2 promoter***	59426 to 59501	5381 to 5457	absent	4221 to 4319
*COLDAIR-like 1***	57359 to 58009	4483 to 5032	2691 to 3685	4320 to 5393
*COLDAIR-like 2***	59502 to 60192	5458 to 6155	2691 to 3685	4320 to 5393

Table S12 Comparison of *VRE*/*VRE-like* and *COLDAIR*/*COLDAIR-like* sequences found in the first intron of *PEP1* and *AmFLC*; intron 1 in *A. alpina* refers to the sequence homologous to *A. montbretiana* intron 1. According to their order in the intron sequences were named VRE1 (more 5' located) and VRE2 (downstream of VRE1) as well as COLDAIR1 (more 5' located) and COLDAIR2 (downstream of COLDAIR1)

		% identity VRE-like 1 vs VRE-like 2	VRE-like 1 % identity to At VRE	VRE-like 2 % identity to At VRE
VRE-like 1 vs VRE-like 2	*A. alpina*	79.17	63.14	59.72
	A. montbretiana	72.60	60.42	60.85
		% identity COLDAIR-like 1 vs COLDAIR-like 2	COLDAIR-like 1 % identity to At COLDAIR	COLDAIR-like 2 % identity to At COLDAIR
COLD-like 1 vs COLD-like 2	*A. alpina*	78.67	65.42	64.07
	A. montbretiana	77.28	63.19	63.48

Table S13 Associated *p*-values for the respective SH tests based on the different single marker datasets. *P*-values indicated in bold are above the threshold of *p* = 0.05. In these cases the hypothesis that the respective alternative topology explains the data equally well is accepted. P-values below the threshold of *p* = 0.05 are indicated with an asterisk. In those cases the hypothesis that the respective alternative topology explains the data equally well is rejected

	orthologue of *At2g13360* dataset	adh dataset	chs dataset	ITS dataset	*trn*LF dataset
all 100 random topologies	0.000*	0.000*	0.000*	0.000*	0.000*
orthologue of *At2g13360* topology	best	**0.259**	**0.385**	**0.279**	**0.456**
ADH topology	**0.620**	best	**0.658**	**0.343**	**0.478**
CHSX topology	**0.658**	**0.798**	best	**0.416**	**0.478**
ITS topology	**0.352**	0.022*	**0.205**	best	**0.334**
*trn*LF topology	**0.129**	0.011*	**0.220**	**0.146**	best

Table S14 (separate file Table_ S14.txt) Alignment of both *FLC* orthologues from *A. nordmanniana* (*AnFLC A* and *B*) to *PEP1* and *AmFLC*; a stretch of N was inserted between the two contigs joined to form *AnFLC A*. The alignment includes a partial sequence of the orthologue of *At5g10150*, exons 1 (A and B in *A. alpina*) to 7 including all intronic sequences (partial intron 6 in *AnFLC A*) as well as ~280bp sequence downstream of the stop codon (includes the COOLAIR promoter (Castaings et al. 2014; Csorba et al. 2014; Swiezewski et al. 2009)

Figure S1 Alignment of cDNA sequences of *AmFLC*, *AauFLC* and *PEP1* used for designing primers for qPCR

```
AauFLC_cDNA   : ATGGG AGAAAAAAACT GAAATCAAACGAATTGAGAACAAAAGTAGCCGACAAGTCACCTTCTCTAAAC :  70
AmFLC_cDNA    : ATGGGTAGAAAAAAACTCGAAATTAAACGAATTGAGAACAAAAGTAGCCGACAAGTCACCTTCTCTAAAC :  70
PEP1_FJ755930 : ATGGGTAGAAAAAAACTCGAAATCAAACGAATTGAGAACAAAAGTAGCCGACAAGTCACCTTCTCTAAAC :  70

                        80         *       100         *       120         *       140
AauFLC_cDNA   : GTCGCAACGGTCTCATCGAGAAAGCTCGTCAGCTTTCTGTTCTCTGCGATGCTTCCGTCGCTCTTCTCGT : 140
AmFLC_cDNA    : GTCGCAACGGTCTCATCGAGAAAGCTCGTCAGCTTTCTGTTCTATGTGATGCCTCCGTAGCTCTCCTTGT : 140
PEP1_FJ755930 : GTCGCAACGGTCTCATCGAGAAAGCTCGTCAGCTTTCTGTTCTATGTGATGCCTCCGTAGCTCTCCTTGT : 140

                         *       160         *       180         *       200         *
AauFLC_cDNA   : CGTCTCCGCCTCCGGCAAACTCTACAGCTTCTCCTCCGGTGATAACCTGGTCAAGATCCTTGATCGATAT : 210
AmFLC_cDNA    : CGTCTCCGCCTCTGGCAAACTCTACAGCTTCTCCTCCGGTGATAACCTGGTCAAGATCCTTGATCGATAT : 210
PEP1_FJ755930 : CGTCTCCTCCTCTGGCAAACTCTACAGCTTCTCCTCCGGTGATAACCTGGTCAAGATCCTTGATCGATAT : 210

                       220         *       240         *       260         *       280
AauFLC_cDNA   : GGAAAAAAACATGAAGATGATCTAAAAGCCCTGGATCTTCAGTC-------------------------- : 254
AmFLC_cDNA    : GGAAAACAACATGCAGATGATCTCAAAGCCCTGGATCTTCAGTC-------------------------- : 254
PEP1_FJ755930 : GGAAAACGACATGCAGATGATCTCAAAGCCCTGGATCTTCAGTCCCTTCTCCATGGACAGGATCTTCAGT : 280

                         *       300         *       320         *       340         *
AauFLC_cDNA   : -AAAAGCTCTGAACTATGGTTCACACCAAGAGCTTCTAGAACTTGTGGAAAGCAAGCTTATGGAACCAAA : 323
AmFLC_cDNA    : -AAAAGCTCTGAACTATGGTTCACACCATGAGCTACTAGAAATTGTTGAAAGCAAGCTCGTGGCACCAAA : 323
PEP1_FJ755930 : CAAAAGCTCTGAACTATGGTTCACACCATGAGCTACTAGAAATTGTTGAAAGCAAGCTCGTGGCACCAAA : 350

                       360         *       380         *       400         *       420
AauFLC_cDNA   : TATCGATAATATAAGTGTCGATACCCTCGTTCAGCTGGAGGAACACCTTGAAACTGCCCTCTCCGTAACT : 393
AmFLC_cDNA    : TGTCAATAACGTAAGTGTCGACACTCTCGTTCAGCTGGAGGAACACCTTGAAACTGCTCTCTCCGTAGTT : 393
PEP1_FJ755930 : TGTCAATAACGTAAGTTTCGACACTCTCGTTCAGCTGGAAAAACACCTTGAAACTGCTCTCGCCGTAGTT : 420

                         *       440         *       460         *       480         *
AauFLC_cDNA   : AGAGCTAAGAAGACAGAACTAATGTTGAAGCTTGTTGAGAGCCTTAAAGAAAAGGAGAAGTTGCTGAAAG : 463
AmFLC_cDNA    : AGAGCTAAGAAGACAGAACTAATGTTGAAGCTTCTTGAGAGCCTTAAAGAAAAGGAGAAATTGCTGAAAG : 463
PEP1_FJ755930 : AGAGCTAAGAAGACAGAACTAATGTTGAAGCTTCTTGAGAGCCTTAAAGAAAAGGAGAAATTGCTGAAAG : 490

                       500         *       520         *       540         *       560
AauFLC_cDNA   : AAGAGAACAAGATTTTGGCTAGCCAGATGGAGAAGAAGAATCTTGTGGGAGCAGAAGCTGATGAGAATAT : 533
AmFLC_cDNA    : AAGAAAACCAGGTTTTGGCTAGCCAGATGGAGAAGAAGACTCTTGTGGGAGCAGAAGCTAATGATAATAT : 533
PEP1_FJ755930 : AAGAAAACCAGGTTTTGGCTAGCCAGATGGAGAAGAAGACTCTTGTGGGAGCAGAAGCTGATGATAATAT : 560

                         *       580         *       600         *       620
AauFLC_cDNA   : GGAGATATCACCTGGAGAAATCTCCGACATCAATCTTCCGGNNNNNNNNNNNNNNNNNNNNNNN : 597
AmFLC_cDNA    : GGAGATATCACCTGGAGAAATCTCCGACATCAATCTTCCGGNNNNNNNNNNNNNNNNNNNNNNN : 597
PEP1_FJ755930 : GGAGATATCACCTGGAGAAATCTCCGACATCAATCTTCCGGTAACTCTCCCACTACTCAATTAA : 624
```

Figure S2 Maximum Parsimony (MP) phylogenetic reconstruction calculated in MEGA5 (Tamura et al. 2011) based on the orthologues of *ADH*, *CHS* and *At2g13360*. For *A. nordmanniana* both co-orthologues were included. The tree is mostly congruent with the tree in Fig. 1 and indicates that the second allele in *A. nordmanniana* not used in the phylogeny in Fig. 1 is sister to the entire group including *A. nordmanniana*, *A. alpina*, *A. purpurea*, *A. montbretiana* and *A. nova* ssp. *iberica*

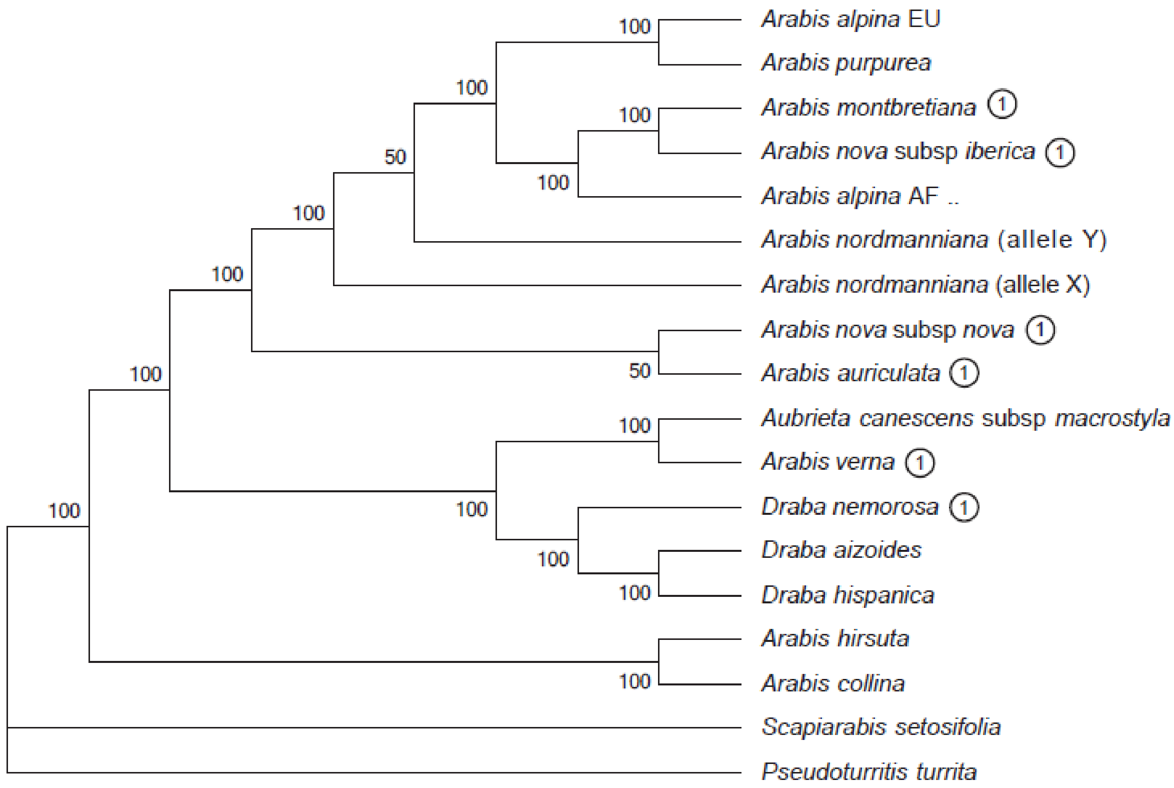

Figure S3 Vernalization response of (A) *A. montbretiana* and (B) *A. auriculata* measured in leaf number. *A. montbretiana* responds already to vernalization when shifted to cold conditions at the age of one week and *A. auriculata* already responds to vernalization at least at an age of 3w; flowering time is given as total leaf number on the main shoot (TLN); before and after vernalization plants were grown under long day (LD) conditions in (A) either in the greenhouse (light gray bars; n=4-8) or in a growth cabinet (dark gray bars; n=12) with similar growth conditions (see methods) or in (B) under different greenhouse conditions (n=11-12); for both species both experiments showed comparable results for each time point n=X, error bars represent standard deviation.

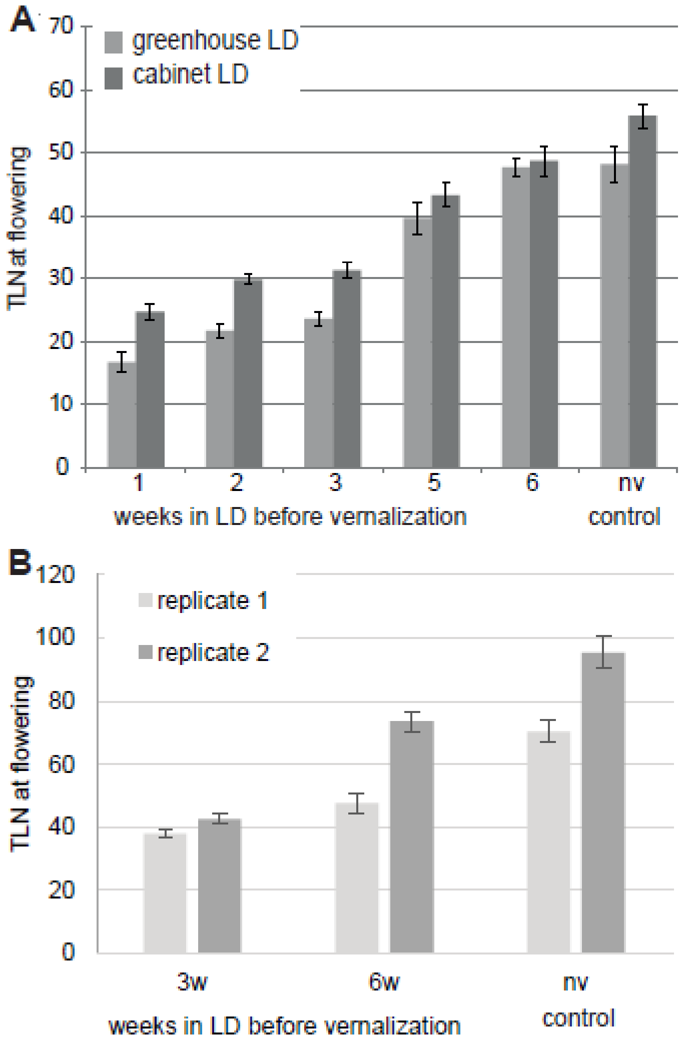

Figure S4 Biological replicates of expression data shown in fig. 2. Relative expression of *FLC* orthologues in perennial *A. alpina* and tetraploid *A. nordmanniana* (allele A and B) and annual *A. montbretiana* and *A. auriculata*. All examined orthologues are expressed before vernalization (0w = 0 weeks vernalization = 3 or 6 weeks at warm temeparture) and repressed by vernalization (12 wv = 12 weeks vernalization at 4 °C). However, only in the perennial species the *FLC* orthologues are derepressed after vernalization while in the annual species they stay stably repressed (12 wv + 2/3w = 12 weeks vernalization plus 2 or 3 weeks at warm temperature). Expression values are relative to the respective orthologues of *PP2A* or *RAN3* (*A. nordmanniana*) and relative to 0 weeks (0w) of vernalization. *AnFLC A* and *B* were amplified together. Error bars represent standard deviation of technical qPCR replicates.

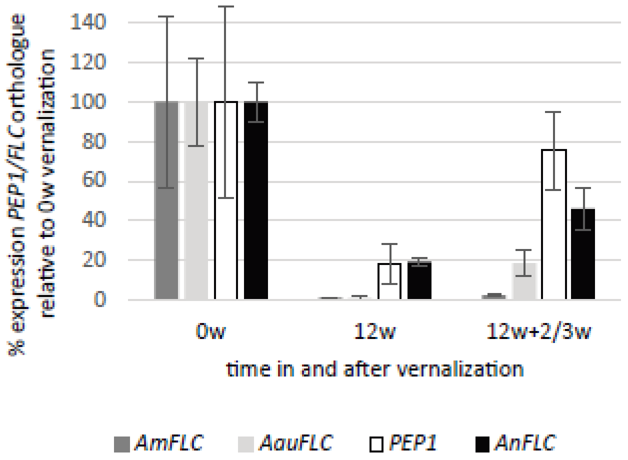

Fig S5 Genotypes of introgression lines (*A. montbretiana* in *A. alpina* acc. Pajares background) used in the expression analysis of *AmFLC* and *PEP1* for confirming the results obtained from the two cuttings of the original F1 plant (*A. montbretiana* x *A. alpina* acc. Pajares). Plants were genotyped with length polymorphism markers and genotypes were plotted by Flapjack (Milne et al. 2010); one row corresponds to a single plant; one column corresponds to one marker; yellow = *A. alpina* homozygous, red = *A. montbretiana* homozygous, red/yellow box = heterozygous, white = missing data; the location of *PEP1*/*AmFLC* is indicated by an arrow; marker numbers as in table S8.

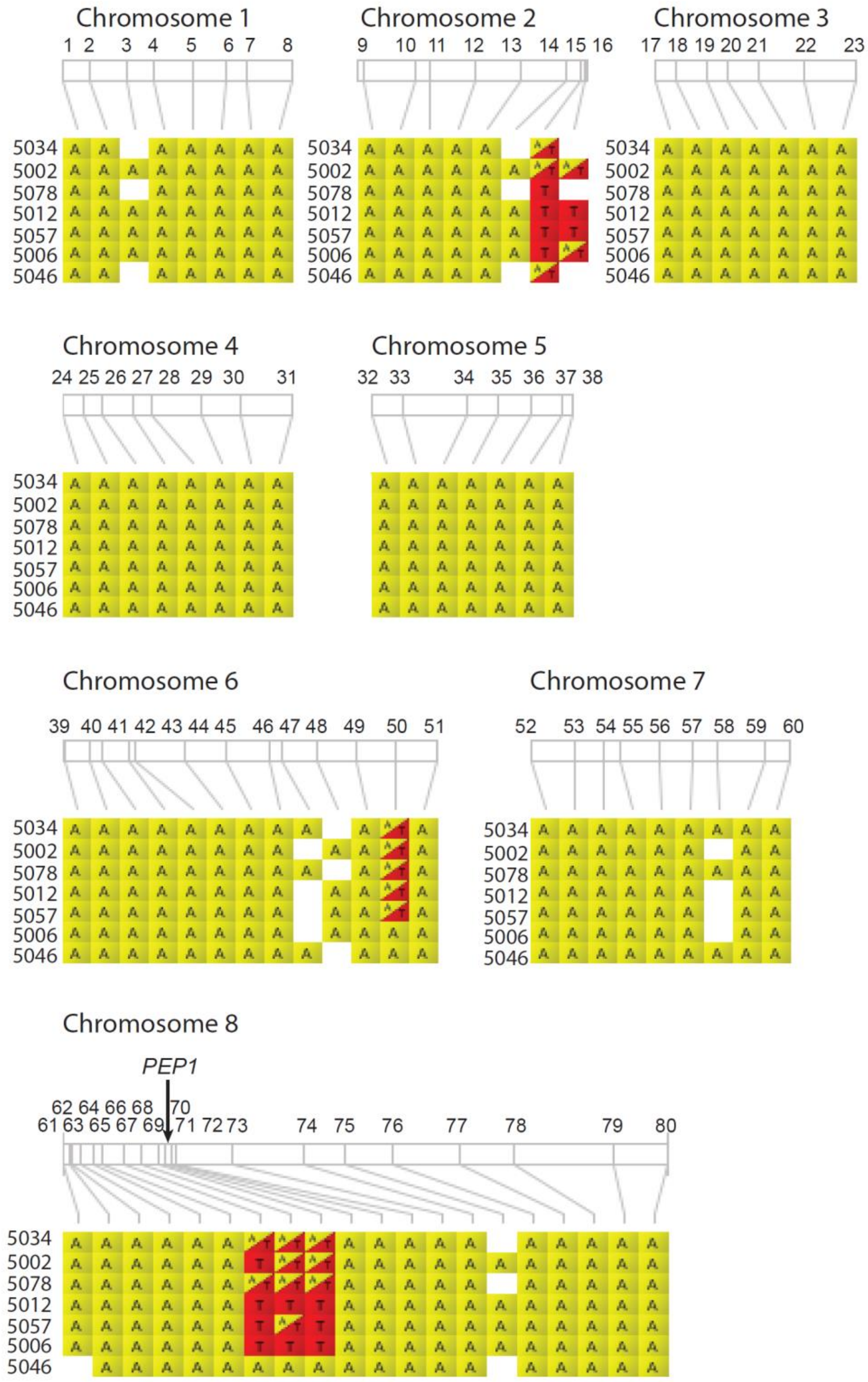

Figure S6 (A) Dot plot of the *AmFLC* genomic region (last exon upstream gene until the end of the COOLAIR promoter) vs. *AmFLC* cDNA; 7 exons can be detected, no exon has been duplicated (B, C, D) Comparison of the *PEP1*, *AmFLC* and *FLC* sequences by GATA plots. Sequences compared include the transcriptional start site, all exons and introns and end with the stop codon of exon 7. (B) GATA plot comparing *AmFLC* and *FLC*; the structure of *AmFLC* resembles the simple structure of *FLC* (C) GATA plot comparing *PEP1* (starting with the ATG of exon 1A) and *FLC* showing the duplication of the first exon and an adjacent segment of the intron as well as of the sequence orthologous to *COLDAIR* (referred to as *COLDAIR*-like 1 and *COLDAIR*-like 2) (D) GATA plot comparing *PEP1* (starting with the ATG of exon 1A) and *AmFLC*; the plot confirms the simpler structure of *AmFLC* but also the presence of *COLDAIR*-like 1 and 2; the 416 bp insertion which allows the differentiation of the duplicated intron next to exon 1A or exon 1B is annotated (E) Schematic view of *PEP1*, *AmFLC*, *FLC* as well as the *A. lyrata* orthologue *AlFLC* showing all exons and introns; sequences with known regulatory function as well as sequences providing insights into the sequence evolution of the locus are color coded (figure p. 30)

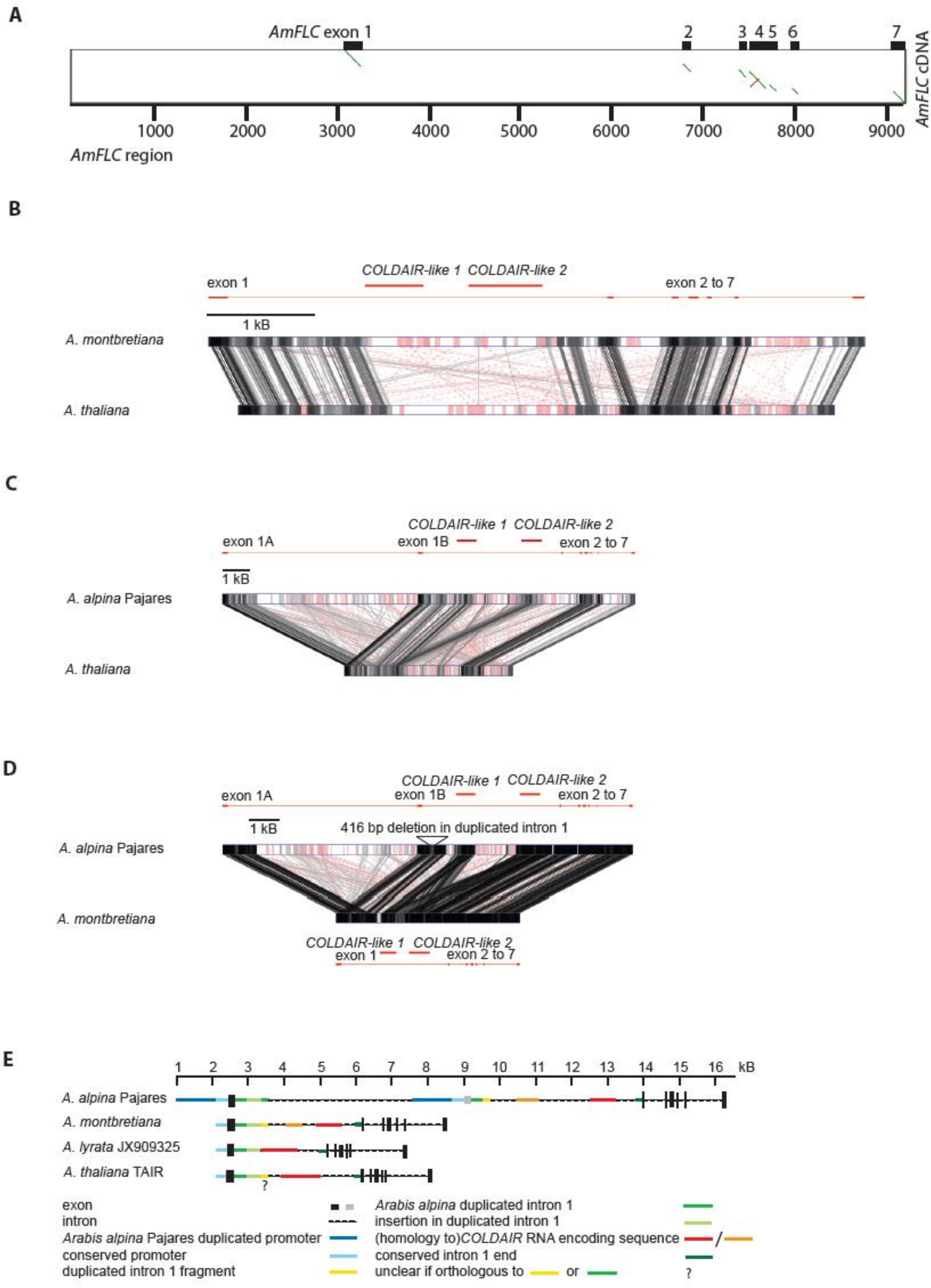
A
AmFLC exon 1
2
3 4 5 6
7
AmFLC cDNA
1000 2000 3000 4000 5000 6000 7000 8000 9000
AmFLC region
B
exon 1
COLDAIR-like 1 COLDAIR-like 2
exon 2 to 7
1 kB
A. montbretiana
A. thaliana
C
COLDAIR-like 1 COLDAIR-like 2
exon 1A
exon 1B
exon 2 to 7
1 kB
A. alpina Pajares
A. thaliana
D
COLDAIR-like 1 COLDAIR-like 2
exon 1A
exon 1B
exon 2 to 7
1 kB
416 bp deletion in duplicated intron 1
A. alpina Pajares
A. montbretiana
COLDAIR-like 1 COLDAIR-like 2
exon 1
exon 2 to 7
E
1 2 3 4 5 6 7 8 9 10 11 12 13 14 15 16 kB
A. alpina Pajares
A. montbretiana
A. lyrata JX909325
A. thaliana TAIR
?
exon
intron
Arabis alpina Pajares duplicated promoter
conserved promoter
duplicated intron 1 fragment
Arabis alpina duplicated intron 1
insertion in duplicated intron 1
(homology to)COLDAIR RNA encoding sequence
conserved intron 1 end
unclear if orthologous to or
?

Figure S7 Statistical test on the SNP distribution in the alignment of *AmFLC* and *PEP1*. The analysis measures whether the observed SNP number in a window is different from a random distribution; the left panel shows the number of SNPs per window, the right panel shows the –log10 of the associated p-values; the dashed line is the threshold above which statistical significance was reached; values were corrected for FDR; five peaks were found to be statistically significant; these peaks are all located in the 5' region of intron 1

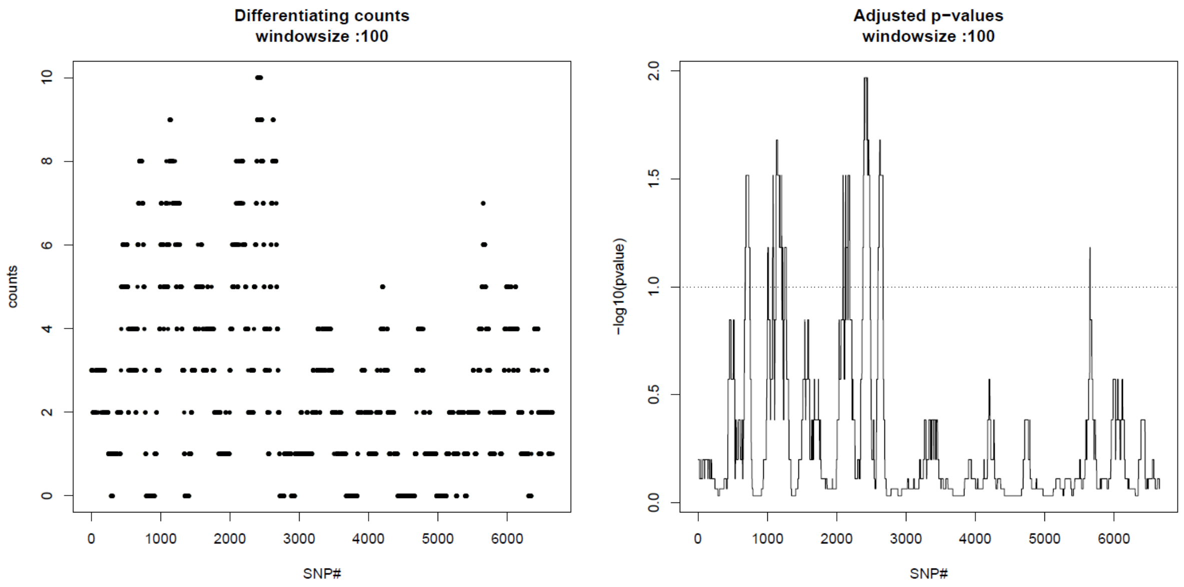

Figure S8 Alignment of the proximal promoter region including UTR of *FLC*, *AmFLC* and *PEP1*; the proximal promoter is defined as the region homologous to what was described as *FLC* minimal promoter (Sheldon et al. 2002)

```
                      *        20         *        40         *        60         *
Aalpina    : ATGC--------GGGAACACGTGGCATATTTTGTCTCGAAATCCCTCACATA---------TTTTATTGT :  53
Amontbreti : ATGC--------GGGTACACGTGGCATATTTTGTCTCTAAATCCCTCACATA---------TTTTATTGT :  53
Athaliana  : ACGCTCGTCATGCGGTACACGTGGCA-ATCTTGTCTTCAA-----------AACACAACGTTTTTATTCA :  58

                     80         *       100         *       120         *       140
Aalpina    : CAT-------------------CTCTCATTTACCCCCAAAC-------------CTTG----ACAAA--- :  84
Amontbreti : CAT-------------------CTCTCATTTACCCCCAAACCAAACAAAAAA-CCTTG----A-AAA--- :  95
Athaliana  : CATATTTGGTTTTTTTGCATCACTCTCGTTTACCCCCAAAAA-AAAAAAAAATATCTGGCCCGACGAAGAA : 127

                      *        160        *        180        *        200        *
Aalpina    : -TAGTAGATTTCACACTAAAAAAAGAAACAAATAAAAGCAAAAA--GGTAAGAAAAAAAA-CACAAAAAA : 150
Amontbreti : -AAGTAGATTTCACACTAAAAAAAGAAACAAATAAAAGCAAAAA--GGGAAGAAAAAAAAACACAAAAAA : 162
Athaliana  : AAAGTAGATAG-GCACAAAAAATAGAAAGAAAT-AAAGCGAGAAAAGG-AAAAAAAAAAA---------- : 184

                    220         *       240         *       260         *       280
Aalpina    : AGAGTGAGAATAGAAAAA-----AAAACGCTTAGTATCTCAGGCGACTTAAGAACCG-AACCTGAGGATC : 214
Amontbreti : -GAGAGAGAATAGAAAA------AAAACGCTTAGTATCTCCGGCGACTTAAGAACCG-AACCTGAGGATC : 224
Athaliana  : ----------TAGAAAGAG----AAAACGCTTAGTATCTCCGGCGACTT--GAACCCAAACCTGAGGAT- : 237

                      *       300         *
Aalpina    : CAAATTAGGGCACAAAGCCTTCTCGGAGACAGAAGCC : 251
Amontbreti : CAAATTAGGGCACAAAGCCCTCTCGGAGACAGAAGCC : 261
Athaliana  : CAAATTAGGGCACAAAGCCCTCTCGGAG--AGAAGCC : 272
```

Figure S9 Alignment of *COLDAIR* and *A. montbretiana* and *A. alpina COLDAIR -like 1* and *COLDAIR -like 2*; yellow positions indicate ancestral positions maintained in *COLDAIR -like 1* while pink positions indicate conservation in *COLDAIR -like 2*; more ancestral positions are maintained in *COLDAIR -like 1*; COLDAIR-likes are defined as regions showing homology to the *Arabidopsis* COLDAIR sequence (Heo and Sung 2011)

```
                             *        20         *        40         *        60         *
Aa_COLDAIR-like_2 : ACCA-CCACACAACCTTG-T-ATCTATTGCA-GTGAA--AATAGACAATTACGCAACC-TTTTGTCTTGC :  63
Am_COLDAIR-like_2 : ACAA-CCACACAACCTTA-T-ATATATTGCA-GTGAA--AATCGACAATGACGCAACC-TTTTGTCTTGC :  63
Aa_COLDAIR-like_1 : ACAAT-CACACAACCTTG-T-GTCTATTCCCCATCAACCAATCGATAATTACACTACC-TTGTATCTTGC :  66
Am_COLDAIR-like_1 : ACAAT-CACACAGCCTTG-T-GTCTATT------------------------------------------- :  25
At_COLDAIR        : ACAATC--CACAACCTCAATCTTTTGTT----GTGAA--AATCGACAATCACACAACCTTTGTATCTTGT :  62

                            80         *       100         *       120         *       140
Aa_COLDAIR-like_2 : ------------------------------GTCTTT-GTCATGGAAATGGACAATAACA-CTGTACCTTT : 101
Am_COLDAIR-like_2 : ------------------------------GTCTTT-GTCATGGAAATGGACAATAACA-CTGTACCTTT : 101
Aa_COLDAIR-like_1 : ------------------------------GTTTTTT-TCATGGAAATGGACAATAACA---CTAACTTT : 102
Am_COLDAIR-like_1 : ------------------------------GTCTTTC--TAAG--A---------AATA---CCAACT-- :  47
At_COLDAIR        : GTCTTTTGTCACACAACCTTTGTATCTTGTGTCTTTTGTCATGGAAATTGTCATTCACACAGCCTTGTTT : 132

                             *       160         *       180         *       200         *
Aa_COLDAIR-like_2 : CTTTGGTGCCTCTTGGAAATCAATAATCCCACCAAAAAACTTTTTTC-TTTCCGGAAGAAATACCAAC-T : 169
Am_COLDAIR-like_2 : CTTTGGTGCCTCTTGGAAATCAATAATCCCACCAAAAAACTTTTTTC-TTTCCGGAAGAAATACCAAC-T : 169
Aa_COLDAIR-like_1 : CTTTGGTGCCTCTTGGAAATCGACAATCCCACCAAAAAACTTGC---CTTTCTAGAAGAAA--------- : 160
Am_COLDAIR-like_1 : ----------------------------------------ATTTAC------------------------ :  53
At_COLDAIR        : CTTTGGTGCCTCTAGGAAATTGAAAATCCCAC---AACACTTGTCTT---CATGTAAGAAATACCAACCT : 196

                           220         *       240         *       260         *       280
Aa_COLDAIR-like_2 : CTTTAGTACGGGTCTATGATGAATTAATATGATCCTACAA------TG-TCAAATTGAATTT-GGTCTGA : 231
Am_COLDAIR-like_2 : CTTTAGTAC-GGTCTATGATGAATTAATATGATCCTACAT------TG-TCAAATTGAATTT-GGTCTGA : 230
Aa_COLDAIR-like_1 : ---TAGTACAGGTCTATGGTGATTCAGTATGATCCTACAT------TGATCAAATTAAATT-TGGCCTGA : 220
Am_COLDAIR-like_1 : ------TACAGGTGTATGGTGATTCAGTATGATCCTACAT------TGATCAAATTAAATT-TGGCCTGA : 110
At_COLDAIR        : CTTTGGTACGGATCTATAATGAATCAATATAATCCTATATATAAGTTGTCAAAATTGAATCT-GG--TGT : 263

                             *       300         *       320         *       340         *
Aa_COLDAIR-like_2 : AGTGTTTACTAAAACCCTCCAACAT--TAACCAAGTAGTTGAAGTGGGTTGGCTATGCTGGTCAAGATCA : 299
Am_COLDAIR-like_2 : AGTGTTTACTAAAACCCTCCAACAT--TAACCAAGTAGTTGAAGTGGGTTGGCTATGCTGGTCAAGATCA : 298
Aa_COLDAIR-like_1 : AGTGTTTACTAGAACCCTCCA--AT--TATCCATGTAGTTGAAGTAGTTTGGCCATGCTGGTCAAGATCA : 286
Am_COLDAIR-like_1 : AGTGTTTACTAAAACCCTCCA--AT--TATCCATGTAGTTTAAGTAGTTTGGCCATGCTGGTCAAGATCA : 176
At_COLDAIR        : AGTGTCTACTACAACCCTCCAATATAATAACCAAATGGTTGTAGTAGTTTGGCCATGTTGGTCAAGATCG : 333

                           360         *       380         *       400         *       420
Aa_COLDAIR-like_2 : TAGGCCGATTCTCACTTGATGCATAATTTGCTAGGATTTGTTCAGGCCGTATACTAAATGTTGGGTCCAA : 369
Am_COLDAIR-like_2 : TAGGCCGATTCTCACTTGATGCATAATTTGCTAGGATTTGTTCAGGCCGTATACTAAATGTTAGGTCCAA : 368
Aa_COLDAIR-like_1 : CAGGTCGATTCTCACTTGATGCATAATTTGCTAGGATTTGTTCAGGCCTTGTACTGAATGCTAGGTCCAA : 356
Am_COLDAIR-like_1 : CAGGTCGATTCTCACTTGATGCATAATTTGCTAGGATTTGTTCAGGCCTTATACTGAATGCTAGGTCCAA : 246
At_COLDAIR        : CTGGCCGATTCTCACTTGATGCATACTTTGTTAGGATTTGTTCACCCCT---------AGTTAGGTCCAG : 394

                             *       440         *       460         *       480         *
Aa_COLDAIR-like_2 : TCTTGGAATAGTC---AAAACTCCAGACTAGGACTTCTGATATTACACATGAAAATCTCAATA----GCT : 432
Am_COLDAIR-like_2 : TCTTGGAATAGTC---AAAACTCCAGACTAGGACTTCTGATATTACACATGAAAATCTCAATA----GCT : 431
Aa_COLDAIR-like_1 : CCTTGGAATAGTC---AAAACTCCAGACTAGGACTTCTGGTATTACA-----AAATCTCAATA----GTT : 414
Am_COLDAIR-like_1 : CCTTGGAATAGTCGTCAAAACTCCAGACTAGGACTTCTGGTATTACA-----AAATCTCAATATATAGTT : 311
At_COLDAIR        : CCTTGGAATTGTC---GAGACACCTGACTAGAACTCCTGGT---------------CTTAA-------TT : 439

                           500         *       520         *       540         *       560
Aa_COLDAIR-like_2 : GTG--TGAGCAAAAATCAAGCCTTTTAGGAATTGGAAACCCTTAGTCAGACAATAACTCATTTGTACATC : 500
Am_COLDAIR-like_2 : GTG--TGAGCAAAAATGAAGCCTTTTAGGAATTGGAAACCCTTAGTCAGACA------CATTTGTACATC : 493
Aa_COLDAIR-like_1 : GTG--TGAGCAAAAAAAA-GCGTTTTAGAAAATGGAAACCCTTAGTCATTCAGTTAATCATTTGTGCATC : 481
Am_COLDAIR-like_1 : GTG--TGAGCAAAAAAAAAGCCTTTTAGAAAATGGAAACCCTTAGTCATTCAGTTAATCATTTGTGCATC : 379
At_COLDAIR        : ATGATTTAATAAAGAAGAAGCCTTTTAGAACGTGG-AACCCTTAGTTACTCAGTTACTCTTTT-GCATA : 507

                             *       580         *       600         *       620         *
Aa_COLDAIR-like_2 : CTTGATTTTGGTGCAAAATAGCTT----AACTTCACAA-------------TA-TTAAGACAAACTATTA : 552
Am_COLDAIR-like_2 : CTTGATTTTGGTGCAAAATAGCTTAATTAACTTCACAACATT---GAAACCTA-TTAACACAAACTATTA : 559
Aa_COLDAIR-like_1 : CTTAATTTCGTTGCAAAGAGCTT----AACTTCATAATATT---GAAACCTAACTAACTCAAACTGTTA : 544
Am_COLDAIR-like_1 : CTTAATTTTGGTGCAAAGAGGTT----AACTTCATAATATT---GAAACCTATTTAACTCAAACTGTTA : 442
At_COLDAIR        : CTTAGGTT-GATG-CAAAGAGCTT----AACTTCACAATAGGACTGATATCTA-TTAACAAAACAAATTA : 570

                           640         *       660         *       680         *       700
Aa_COLDAIR-like_2 : AGTGAACTAGTGGAACTTTCGTCAAATCTGCTTTGACATCTGTAAGGCCGTAATATAGAAAAATATAGTA : 622
Am_COLDAIR-like_2 : AGTGAACTAGTGGAACTTTCGTCAAATCTGCTTTGACATCTGTAAGGCCGTAATATAGAAAAATATTGTA : 629
Aa_COLDAIR-like_1 : AGTCAATTAGTGGAATTTTCGTCAAATTTGCTTTGACATATTTAAGTC----ATATAAAAAAATATTGTA : 610
Am_COLDAIR-like_1 : AGTCAATTAGTGGCATTTTCGTCAAATTTGCTTTGACATCTATAAGTC----ATATAAAAAAATATTGTA : 508
At_COLDAIR        : AGT---------GAAGTTTTGTCAAAATTG--TTGG-ATCTTCTAGG-----TCA-------ATAT-GTA : 615

                             *       720         *       740
Aa_COLDAIR-like_2 : GCTGTAGTTTCCCCTAA-TTTTA-TCTGTCGGTTCCTTTTCTA : 663
Am_COLDAIR-like_2 : GCTGCAGTTTCCCCTAA-TTTTA-TCTGTCGGTTCCTTTTCTA : 670
Aa_COLDAIR-like_1 : GTTGTAGTTTCCCCGAT-TTTTG-TATGTCGTTTCCTTCTATA : 651
Am_COLDAIR-like_1 : GTTGCAATTCCCCAATT-TTTTTTATGTCGTTTCCTTCTATA : 550
At_COLDAIR        : GTT-TAGTTTTTAT-CTGTCTTA----GTCGCTTCCTTCTATG : 652
```

Figure S10 Alignment of *VRE* and *A. montbretiana* and *A. alpina VRE -like 1* and *VRE -like 2*; VRE-like sequences are defined as sequences showing homology to the Vernalization Response Element of *FLC* in *Arabidopsis thaliana* (Sung et al. 2006).

```
Am_VRE_like_2 : TCTT------------------------------------------------------------------
Aa_VRE_like_2 : TCTT------------------------------------------------------------------
Aa_VRE_like_1 : TATATACTCCCTCTGTTGCAATTTAGTTGTCATTCTACATGATTTCACGTATATTAAGAAAACATATTAA
Am_VRE_like_1 : TATA------------------------------------------------------------------
VRE           : TAGA------------------------------------------------------------------

Am_VRE_like_2 : ----------------------------------------------------------------------
Aa_VRE_like_2 : ----------------------------------------------------------------------
Aa_VRE_like_1 : AAATTCTATTATACCCTTAAAAACAAAGTTAACTCTTGGTATTCTTCATAAAATTTGAGGTTAAAGTTGG
Am_VRE_like_1 : ----------------------------------------------------------------------
VRE           : ----------------------------------------------------------------------

Am_VRE_like_2 : ----------------------------------------------------------------------
Aa_VRE_like_2 : ----------------------------------------------------------------------
Aa_VRE_like_1 : TATTCAAAGCTAAAAATTACATTGAAAATTGGAAACGACAACTAATTTGAAACATAAAATTAAGTCTAAA
Am_VRE_like_1 : ----------------------------------------------------------------------
VRE           : ----------------------------------------------------------------------

Am_VRE_like_2 : ------------------------------GGTAAATTTT-----------------------CACCCTT
Aa_VRE_like_2 : ------------------------------GGTAAATTTT-----------------------CACCCTT
Aa_VRE_like_1 : ACGACAACTAATTTGCAACGGAGGGAGTATGCTAAATATTTAAGTGATTGGATATCAA-TTATAACCCTT
Am_VRE_like_1 : ---------------------------TATGCTAAATATTTATGTGATTGGATATCAC-TTATAACCCTT
VRE           : ---------------------------TTTGCCTCATATTTATGTGATTGTATATCAAT-TATCGCCCTT

Am_VRE_like_2 : AATCTTATATTTG---TGTGCAATGCATAAATTTAGCTTGTAGCTTTGGTCATTGAAAT--CGACAACCA
Aa_VRE_like_2 : AATCTTATATCTG---TGTGCAATGCATAAATT-AGCTTGTAGCTTTGGTCACT-AAAAC-CGACCACCA
Aa_VRE_like_1 : AATCT-ATATCTGTTGTGTGCAATGTATGAATTTAACTGGTAGCTTTGGTCGTTGGAAA--TGACAATCA
Am_VRE_like_1 : AATCT-ATATCTGTTGGGTGCAATGTATGAATTTAACTGGTAGCTTTGGTCGTTGGAAA--TGACAATCA
VRE           : AATCTTATCATCGTTGTGTTCATT-TATGACTTTGT--------TCCTATTCGTTAAAA-TTGACAATC-

Am_VRE_like_2 : CACAACCTTATAT-ATATT-GCA--GTGAAAATCGACAATGACGCAACCTTTTGT--CTTGCG-------
Aa_VRE_like_2 : CACAACCTTGTAT-CTATT-GCA--GTGAAAATAGACAATTACGCAACCTTTTGT--CTTGCG-------
Aa_VRE_like_1 : CACAACCTTGTGT-CTATTCCCCATCAACCAATCGATAATTACACTACCTT--GTATCTTGCG-------
Am_VRE_like_1 : CACAGCCTTGTGT-CTATT-------------------------------------------G-------
VRE           : CACAACC-T-CAATCTTTT-GTT--GTGAAAATCGACAATCACACAACCTT-TGTATCTTGTGTCTTTTG

Am_VRE_like_2 : -----------------------TCTTT-GTCATGGAAATGGACAATAACACTGTACCT----TTCTTTG
Aa_VRE_like_2 : -----------------------TCTTT-GTCATGGAAATGGACAATAACACTGTACCT----TTCTTTG
Aa_VRE_like_1 : ------------------------TTTTTTTCATGGAAATGGACAATAACACT--AACT----TTCTTTG
Am_VRE_like_1 : -----------------------TC-TTTCTAA--GAAAT------------------------------
VRE           : TCACACAACCTTTGTATCTTGTGTCTTTTGTCATGGAAATTGTCATTCACACA--GCC-TTGTTTCTTTG

Am_VRE_like_2 : GTGCCTCTTGGAAATCAATAATCCCACCAAAAAC-TTTTTTCTTTCCGGAAGAAATAC
Aa_VRE_like_2 : GTGCCTCTTGGAAATCAATAATCCCACCAAAAAC-TTTTTTCTTTCCGGAAGAAATAC
Aa_VRE_like_1 : GTGCCTCTTGGAAATCGACAATCCCACCAAAAAC---TTGCCTTTCTAGAAGAAA---
Am_VRE_like_1 : ----------------------------------------------------------
VRE           : GTGCCTCTAGGAAATTGAAAATCCCACAACA-----CTTGTCTTCATGTAAGAAATAC
```

Figure S11 Alignment of the *A. montbretiana* and *A. alpina* region homologous to the nucleation region in *A. thaliana* defined by the ChIP primers from (Yang et al. 2014)

```
                          *        20         *        40         *        60         *
A_alpina        : ATGGGTAGAAAAAAACTCGAAATCAAACGAATTGAGAACAAAAGTAGCCGACAAGTCACCTTCTCTAAAC :  70
A_montbretiana  : ATGGGTAGAAAAAAACTCGAAATTAAACGAATTGAGAACAAAAGTAGCCGACAAGTCACCTTCTCTAAAC :  70
A_thaliana      : ATGGGAAGAAAAAAACTAGAAATCAAGCGAATTGAGAACAAAAGTAGCCGACAAGTCACCTTCTCCAAAC :  70
A_lyrata        : ATGGGGAGAAAAAAACTAGAAATCAAGCGAATTGAGAACAAAAGTAGCCGACAAGTCACCTTCTCCAAAC :  70

                         80         *       100         *       120         *       140
A_alpina        : GTCGCAACGGTCTCATCGAGAAAGCTCGTCAGCTTTCTGTTCTATGTGATGCCTCCGTAGCTCTCCTTGT : 140
A_montbretiana  : GTCGCAACGGTCTCATCGAGAAAGCTCGTCAGCTTTCTGTTCTATGTGATGCCTCCGTAGCTCTCCTTGT : 140
A_thaliana      : GTCGCAACGGTCTCATCGAGAAAGCTCGTCAGCTTTCTGTTCTCTGTGACGCATCCGTCGCTCTTCTCGT : 140
A_lyrata        : GTCGCAACGGTCTCATCGAGAAAGCTCGTCAGCTTTCTGTTCTCTGTGACGCATCCGTCGCTCTTCTCGT : 140

                          *       160         *       180         *       200         *
A_alpina        : CGTCTCCTCCTCTGGCAAACTCTACAGCTTCTCCTCCGGTGATAAGTATGACTTCTCCTTTTCTTGATTT : 210
A_montbretiana  : CGTCTCCGCCTCTGGCAAACTCTACAGCTTCTCCTCCGGTGATAAGTATGACTTCTCCTTTTCTTGATTT : 210
A_thaliana      : CGTCTCCGCCTCCGGCAAGCTCTACAGCTTCTCCTCCGGCGATAAGTACGCCTTTTCCTTACC-TGGGTT : 209
A_lyrata        : CGTCTCTGCTTCCGGCAAGCTCTACAGCTTCTCCTCCGGCGATAAGTACGCCTCTTCCTTATC-TGGGTT : 209

                        220         *       240         *       260         *       280
A_alpina        : TTCCTTTATTTCCCCTTTGTTTTTAAGCTTCCC-----TTTACTTTTCTGACAAAAAAAATAACCA---A : 272
A_montbretiana  : TTCGTTTATTTCCCCTTTGTTTTTAAGCTTCCC-----TTTACTTTTCTGACAACAAAAA-AACT----- : 269
A_thaliana      : TTCATTTGTTCCCCCTTTTATCTTCTGTTTTGTGCTCTTTTACTTTTCTGAGAAA------------ATA : 267
A_lyrata        : TCCATTTATTTCCCCTTCAATCTTCAGTTTTGTGCTC-TTTACTTTTCTGAAAAATAAAAATAAAAGACA : 278

                          *       300         *       320         *       340         *
A_alpina        : AAAAAAAAAACACAATTATTGAAATAGTTT----TGATTTTTCCGGCGAATCTATTGTGGGTTTCTTGAG : 338
A_montbretiana  : AAAATAAAAACACAATTATTGAAAAAGTTT----TGATTTTTCCGGCGAATCTATTGTGGGTTTCTTGAG : 335
A_thaliana      : AAAATAAAAAAACAATTAATATACCGTTTG----GTTTTTTTCCGGCGGATCTCTTGT-TGTTTCTCGGT : 332
A_lyrata        : AAAATAAAAAAACAATTAATAAACCGTTTGTTTTTTTTTTTTCCGGCGAATCTCTTGT-TGTTTCTCGGT : 347

                        360         *       380         *       400         *       420
A_alpina        : TTTGTGTTTGTTACTGG-TTTTTCTATGGACATGAGAGATACATGAGTCAACTAAATTTGAGAAAGAACA : 407
A_montbretiana  : TTTGTGTTTGTTACTAG-TTTTTCTATGGACATGAGAGATACATGAGATAACTAAATTTGAGAAAGAACA : 404
A_thaliana      : TCTGTGTTTGTTTGTGTTTTTTTCTGCGACCATGATAGATACATGAGATAACCAAATTTAAGGAAGAACA : 402
A_lyrata        : TCTGTGTTTGTTAGTGTCTTTTTCTATGACCATGAGAGATACATGAGATAACCTAATTTAAGGAAGAACA : 417

                          *       440         *       460         *       480         *
A_alpina        : ATGTCGTGAT---------GAGC--TAATGCTTCTTATACTTTCTTTCACTTCTCTCTCTCTCTTTTTCT : 466
A_montbretiana  : ATGTCGTGAT---------GAGC--TAAAGCTTCT--TACTTTCTTTCACTTCTCTCTCTCTCTCT---- : 457
A_thaliana      : ATGTCGTGAA--------GAAGCTTTTTAGCTTCT--TACTTTTGTTCATTTCTCTCTC--T-------- : 452
A_lyrata        : ATGTCGTGAACTGGTGATGAAGC--TAAAGCTTCT--TACTTTTGTTCATTTCTCTCTCTAT-------- : 475

                        500         *       520         *       540         *       560
A_alpina        : TGATTTTCTTGACTTTCTTGACCTTTTTTTGCTGCATGGTTTT--TTCTTTATATTGGTATAAAATTTGC : 534
A_montbretiana  : ---TTTTCTTGATTTTCTTGAAC-TTTTTTGCTGCATGTTTTT--TTATTT-TATTGGTTAAAAATTTGC : 520
A_thaliana      : ---ATTTCTTAAAAAAAAAAATT--------CTGCATGGATTTCATTATTT-CCTTGGAAAAAAAT-TGC : 509
A_lyrata        : ---ATTTCTT---------AATTTTTTTTTCCTGCATGGATTTTATTATTT-TCTTTGGAAATAATATGC : 532

                          *       580         *       600         *       620         *
A_alpina        : ACGTCTTTCAAGATCTTTTTGATACGTTGTGATG--------------CATAATTGATGTTGTTTAGTGT : 590
A_montbretiana  : ATGTGTTTCAAGATGTTTTTGATACGTTGTGATG--------------CATAATTGATGTTGTTTAGTGT : 576
A_thaliana      : ATGTCATTCACGATTTGTTTGATACGATCTGATG--------------CGTGCTCGATGTTGTTGAGTGA : 565
A_lyrata        : ATGTCCTTCACGATTTGTTTGATACGATCTGATGCGTGCTCCGTGCTCCGTGCTCGATGTTGTTTAGTGA : 602

                        640         *       660         *       680         *       700
A_alpina        : AGTTTCAAGCTATCTTTGATTGTTTGTTTGTTCATCACTTGTTAATTGGCGTTAAAGAGTTGTTTACTAG : 660
A_montbretiana  : AGTTTCAAGCTATCTTTGATTGTTTGTTTGTTCATCACTTGTTAATTGGCGTTAAAGAGTTGTTTACTAG : 646
A_thaliana      : AGTTTCAAGCCATCTTTGATTGTTTCTTACCTTTAGAGATTCCTTAAGTTTTTGAAGAGTTAATTAT--- : 632
A_lyrata        : AGTTTCAAGCCATCTTTGATTGTTACTTTGCTTTAGAGATTCCTTAAGTTTTTGAAGAGTT--------- : 663

                          *       720         *       740         *       760         *
A_alpina        : ATTGATTAATGAATAACGATTACTGTTTCTGTTTGTAGAGTAATTGAAA-TCATTGGATCTCTCGGATTT : 729
A_montbretiana  : ATTGATTAATGAATAACGATTACTGTTTCTGTTTGTAGAGTAATTGAAA-TCATTGGATCTCTCGGATTT : 715
A_thaliana      : -ATATCACAAGACTAATGATTAATG--TCTCTTTTCAGAGTGATTAAAATTCATTGGATCTCTCGGATTT : 699
A_lyrata        : ----------GACTAATGATTAATGTCTCTCTTTTCAGAGTGATTAAAATTCATTGGATCTCTCGGATTT : 723

                        780         *       800
A_alpina        : GTATGCAATGCAATACAATGCACAT-CGGGA : 759
A_montbretiana  : GTATGCAATG-----CAATGCACAT-CGGGA : 740
A_thaliana      : GTATGC----------AATGCACTTACGGGA : 720
A_lyrata        : GTATGCAATG-----CAATGCACCTACGGGA : 749
```

Figure S12 *Arabis montbretiana* life cycle monitored under greenhouse conditions. Also under controlled conditions the species grows as an annual undergoing complete senescence after flowering and setting seeds (A) at 24 days long day (LD), (B) at 50 days LD, (C) at 71 days LD, (D) at 99 days LD, (E) after senescence

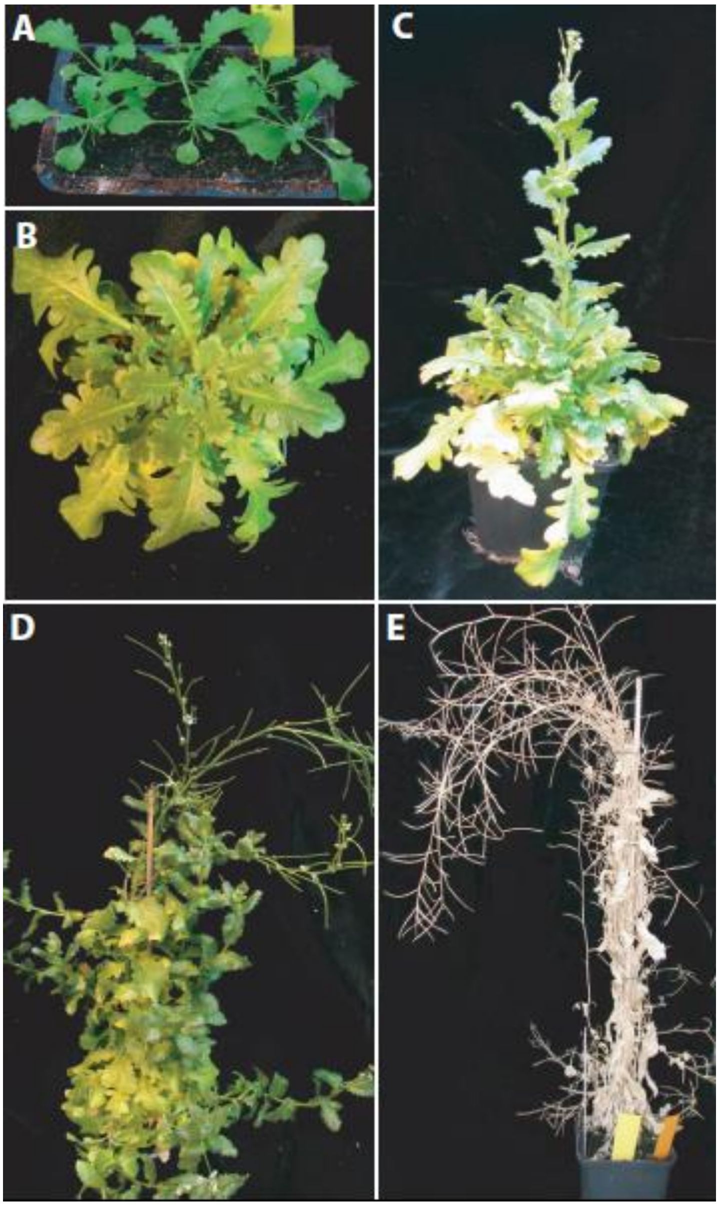

Figure S13 Alignment of *PEP1* exon 1A and 1B and *AmFLC* exon 1 along with adjacent intron sequences; the 416 bp used to differentiated copy 1A and copy 1B are indicated by $ while the SNPs differentiating the duplicated segment of intron 1 in PEP1 are marked by *

```
Aa_1A  : ATGGGTAGAAAAAAACTCGAAATCAAACGAATTGAGAACAAAAGTAGCCGACAAGTCACCTTCTCTAAAC :   70
Aa_1B  : ATGGGTAGAAAAAAACTCGAAATCAAACGAATTGAGAACAAAAGTAGCCGACAAGTCACCTTCTCTAAAC :   70
Am     : ATGGGTAGAAAAAAACTCGAAATTAAACGAATTGAGAACAAAAGTAGCCGACAAGTCACCTTCTCTAAAC :   70

Aa_1A  : GTCGCAACGGTCTCATCGAGAAAGCTCGTCAGCTTTCTGTTCTATGTGATGCCTCCGTAGCTCTCCTTGT :  140
Aa_1B  : GTCGCAACGGTCTCATCGAGAAAGCTCGTCAGCTTTCTGTTCTATGTGATGCCTCCGTAGCTCTCCTTGT :  140
Am     : GTCGCAACGGTCTCATCGAGAAAGCTCGTCAGCTTTCTGTTCTATGTGATGCCTCCGTAGCTCTCCTTGT :  140

Aa_1A  : CGTCTCCTCCTCTGGCAAACTCTACAGCTTCTCCTCCGGTGATAAGTATGACTTCTCCTTTTCTTGATTT :  210
Aa_1B  : CGTCTCCTCCTCTGGCAAACTCTACAGCTTCTCCTCCGGTGATAAGTATGACTTCTCCTTTTCTTGATTT :  210
Am     : CGTCTCCGCCTCTGGCAAACTCTACAGCTTCTCCTCCGGTGATAAGTATGACTTCTCCTTTTCTTGATTT :  210

Aa_1A  : TTCCTTTATTTCCCCTTTGTTTTTAAGCTTCCCTTTACTTTTCTGACAAAAAAAATAACCAAAAAAAAAA :  280
Aa_1B  : TTCCTTTATTTCCCCTTTGTTTTTAAGCTTCCCTTTACTTTTCTGACAAAAAAAATAACCAAAAAAAAAA :  280
Am     : TTCGTTTATTTCCCCTTTGTTTTTAAGCTTCCCTTTACTTTTCTGACAACAAAAA-AACT--AAAATAAA :  277

Aa_1A  : AACACAATTATTGAAATAGTTTTGATTTTTCCGGCGAATCTATTGTGGGTTTCTTGAGTTTGTGTTTGTT :  350
Aa_1B  : AACACAATTATTGAAATAGTTTTGATTTTTCCGGCGAATCTATTGTGGGTTTCTTGAGTTTGTGTTTGTT :  350
Am     : AACACAATTATTGAAAAAGTTTTGATTTTTCCGGCGAATCTATTGTGGGTTTCTTGAGTTTGTGTTTGTT :  347

Aa_1A  : ACTGGTTTTTCTATGGACATGAGAGATACATGAGTCAACTAAATTTGAGAAAGAACAATGTCGTGATGAG :  420
Aa_1B  : ACTGGTTTTTCTATGGACATGAGAGATACATGAGTCAACTAAATTTGAGAAAGAACAATGTCGTGATGAG :  420
Am     : ACTAGTTTTTCTATGGACATGAGAGATACATGAGATAACTAAATTTGAGAAAGAACAATGTCGTGATGAG :  417

Aa_1A  : CTAATGCTTCTTATACTTTCTTTCACTTCTCTCTCTCTCTTTTTCTTGATTTTCTTGACTTTCTTGACCT :  490
Aa_1B  : CTAATGCTTCTTATACTTTCTTTCACTTCTCTCTCTCTCTTTTTCTTGATTTTCTTGACTTTCTTGACCT :  490
Am     : CTAAAGCTTCT--TACTTTCTTTCACTTCTCTCTCTCTCTCT-------TTTTCTTGATTTTCTTGAAC- :  477

                                                      *  $$$$$$$$$$$$$$$$$$$$$$
Aa_1A  : TTTTTTGCTGCATGGTTTTTTCTTTATATTGGTATAAAATTTGCACGTCTTTCAAGATCTTTTTGATACG :  560
Aa_1B  : TTTTTTGCTGCATGGTTTTTTCTTTATATTGGTATAAAATTTGCATGT---------------------- :  538
Am     : TTTTTTGCTGCATGTTTTTTTATTT-TATTGGTTAAAAATTTGCATGTGTTTCAAGATGTTTTTGATACG :  546

         $$$$$$$$$$$$$$$$$$$$$$$$$$$$$$$$$$$$$$$$$$$$$$$$$$$$$$$$$$$$$$$$$$$$$$
Aa_1A  : TTGTGATGCATAATTGATGTTGTTTAGTGTAGTTTCAAGCTATCTTTGATTGTTTGTTTGTTCATCACTT :  630
Aa_1B  : ---------------------------------------------------------------------- :    -
Am     : TTGTGATGCATAATTGATGTTGTTTAGTGTAGTTTCAAGCTATCTTTGATTGTTTGTTTGTTCATCACTT :  616

         $$$$$$$$$$$$$$$$$$$$$$$$$$$$$$$$$$$$$$$$$$$$$$$$$$$$$$$$$$$$$$$$$$$$$$
Aa_1A  : GTTAATTGGCGTTAAAGAGTTGTTTACTAGATTGATTAATGAATAACGATTACTGTTTCTGTTTGTAGAG :  700
Aa_1B  : ---------------------------------------------------------------------- :    -
Am     : GTTAATTGGCGTTAAAGAGTTGTTTACTAGATTGATTAATGAATAACGATTACTGTTTCTGTTTGTAGAG :  686

         $$$$$$$$$$$$$$$$$$$$$$$$$$$$$$$$$$$$$$$$$$$$$$$$$$$$$$$$$$$$$$$$$$$$$$
Aa_1A  : TAATTGAAATCATTGGATCTCTCGGATTTGTATGCAATGCAATACAATGCACATCGGGAGATCTATGGAA :  770
Aa_1B  : ---------------------------------------------------------------------- :    -
Am     : TAATTGAAATCATTGGATCTCTCGGATTTGTATGCAATGCAA-----TGCACATCGGGAGATCTATGGAA :  751

         $$$$$$$$$$$$$$$$$$$$$$$$$$$$$$$$$$$$$$$$$$$$$$$$$$$$$$$$$$$$$$$$$$$$$$
Aa_1A  : TTGCTATAGGGTTAATGCTTAAATACTAATACATCATCAATTTTGATTTGAATGTATGCCTCATTGTGGC :  840
Aa_1B  : ---------------------------------------------------------------------- :    -
Am     : TTGCTATGGGGTTAATGCTTAAATACTAATAGATCAACAATTTTGATTTGAATGTATGCCTCATTGTGGC :  821

         $$$$$$$$$$$$$$$$$$$$$$$$$$$$$$$$$$$$$$$$$$$$$$$$$$$$$$$$$$$$$$$$$$$$$$
Aa_1A  : AGCTA---------TTATTATATTGTAGATATCACTGTACCACGGATGTATTACTTGAAGATTATCCAAA :  901
Aa_1B  : ---------------------------------------------------------------------- :    -
Am     : AGCTAGTGACTGAAAGATTATACTGTAGATCTCACTTTACCACGGATGTATTACTTGAAGATTATCCAAA :  891

         $$$$$$$$$$$$$$$$$$$$$$$$$$$$$$$$$$$$$$$$$$$$$$$$$$$$$$$$$$$$$$$$$$$$$$
Aa_1A  : TTCATGAAGGTTTCTAAATGAATCTTAATTATCATATATCTATATATGTAGTACTTTTGAATTTTGGTAG :  971
Aa_1B  : -----------------------------------------------------CTTTTGAATTTTGGTAG :  555
Am     : TTCATGAAAGTTTCTAAATGAATCTTAATTATCA----CGTATATATATAGTACTTTTGAATTTTGGTAG :  957

                                                                              *
Aa_1A  : ATTCAACAGATTAAGGAAATTCATGATGCTTACAATTAGTACAAAACAGCACATATTACTTATGTGATCT : 1041
Aa_1B  : ATTCAACAGATTAAGGAAATTCATGATGCTTACAATTAGTACAAAACAGCACATATTACTTATGTGATCC :  625
Am     : ACTCGACAGATTAAGGAAACCCATGAAGCTTACAATTAGTACAAAACAGCACATATTACTTATGTGATCC : 1027

                                       *
Aa_1A  : GGTATCGATTGTGATTCTTTTAATTGCAATAGTTGCGGAAATATATAACACTTTGTGCTGTACATTAAGT : 1111
Aa_1B  : GGTATCGATTGTGATTCTTTTAATTGCAATCGTTGCGGAAATATATAACACTTTGTGCTGTACATTAAGT :  695
Am     : GGTGTCGATTGTGATTCTTTTAATTGCAATCGTTGCGAAAATATATAACACTTTGTGCTGTACACAAAGT : 1097

                                                 *
Aa_1A  : ACATTACATCAATTAGTCTATTTTTAGACTATTAAATCACAATTCTCT : 1159
Aa_1B  : ACATTACATCAATTAGTCTATTTTTAGACTATTAAATCACGATTCTCT :  743
Am     : ACAGTACATCAATTAGTCTATTTTTAGACTATTAAATCACGATTCTCT : 1145
```

Figure S14 Kmer abundance distribution. The genome size can be estimated by the dividing the total number of kmers in the sequencing data by the expected kmer depth. The expected kmer depth is denoted by the red line.

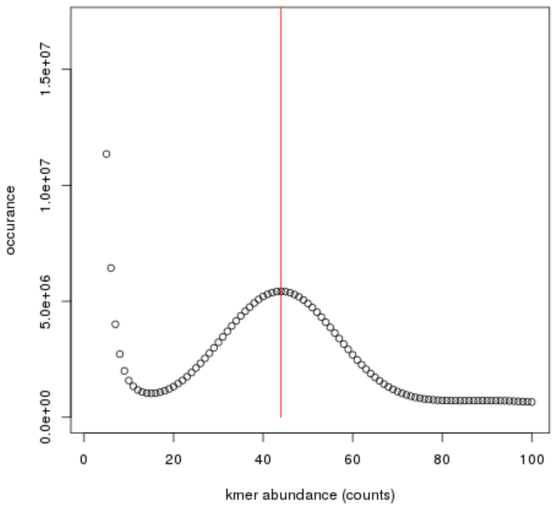

Figure S15 Ends of concatenated contigs which were found to show homology to *FLC* and which were shown to be part of the same molecule (*AnFLC B*) based on PCR and subsequent Sanger sequencing; All contigs showed overlapping ends. The beginning of the overlaps is indicated in green. The end of the first and beginning of the second contig are shown in (A), the end of the second contig used and the beginning of thrid contig used as well as the end of the third contig and the beginning of the fourth contig are shown in (B)

A

```
AnFLC_A    : --------TCTTCTCTCCAGCCTGGTCAAGATCCTTGATCGATATGGAAAACAACATGCAGATGATCTCA :
AnFLC_B    : TTTTTTTGTCTTCTCTCCAGCCTGGTCAAGATCCTTGATCGATATGGAAAAGACAGGCAGATGATCTCA :

AnFLC_A    : AAGCCCTGGTAAAACAAATAAATTCGAGTCTTTTCCATTGTGTATTT---TTTATGGCATAGGTTTAGTAA :
AnFLC_B    : AAGCCCTGGTAAAACAAGCATTTTGAATATTTTCCTTGCTGCAG---TGTTTGAGGCATATATTTACCAA :

AnFLC_A    : AGGCCTAGAGAGTTCCGTACTGG--------TCAGAGACAACTCATAGCCTTGG---------------- :
AnFLC_B    : AGGCCTAGAGAGTTCCGTACTGGTTAGAGGCTCAGAGCCAATTCATAGCATCGAGTTTCAGCTTCCATTT :

AnFLC_A    : ---------------------------------------------------------------------- :
AnFLC_B    : TATGGGCATGGGGCCATGGNNNNNNNNNNTTACCAAAGGCCTAGAGAGTTCCGTACTGGTTAGAGGCTCA :

AnFLC_A    : --------------------GTTTCAGCTTGTTGTATTTTGTGAGCATGG--------ACTCACA-CTCA :
AnFLC_B    : GAGCCAATTCATAGCATCGAGTTTCAGC---TTCCATTTTATGGGCATGGGGCCATGGGCTCACACCTCA :

AnFLC_A    : TATAATCTTTGCGTATATAGTTACT-AGTTTGGAGCTGTAGTTATCAAAGTTTATTTTCAAGTTCATCAA :
AnFLC_B    : TATAATGTTTGCACATATAGTTACTCATTTTGGAGCTGTA-------------GTTATCAAGT---ACAA :

AnFLC_A    : ATAGGTTATGGAGAGA----TTAATGCGCCTTAATTAGGGAATGCATGTTCTATAGACATGAGTAA-ACT :
AnFLC_B    : ATAGGTTTTGGAGAGTTTAATTAATGCTCGTGATCTGGGAATAAATATTTCATAGACATGAGTTTGACG :
```

B

```
AnFLC_A    : -------------------TATATATATGAGGTGGTGGATCCACATGCATATATACTCGAATTCTCAAAG :
AnFLC_B    : -----------AATGATCCTATACATATGAGGTGGTGGATCCA----CATGCATACCCAGATTTTCAAAG :

AnFLC_A    : CCATTA-TCTTTTACTGCCTCTTCT---------------------------GTCCCTTCTCCATTGACA :
AnFLC_B    : CCATTA-TCTTTTACCACCTTTTCT---------------------------GTCCCTTCTCCATGGACA :

AnFLC_A    : GGATCTTCAGTCAAAAGCTCTGAA----------------------------------CTATGGTTCACA :
AnFLC_B    : GGATCTTCAGTCAAAAGCTCTGAACTATGGTTCACACCATNNNNNNNNNNGCTCTGAACTATGGTTCACA :

AnFLC_A    : CCATGAGCTACTAGAACTTGTGGAAAGGTTAGTAC------TAACTAAGAATATTTTTTCTCTCCTCGTT :
AnFLC_B    : CCATGAGCTACTAGATCTTGTGGAAAGGTTA------GTTCTAACTAAGACTATTTTTACTCTCCTCCAT :

AnFLC_A    : TGATCACAAAGGAATTAGGGTTTTTTGTCAATCTATGAATATATGCAGCAAGCTCGTGGAACCAAATATC :
AnFLC_B    : TGATCACTAAGGAATTAGGGTTTCTTGTCAATCTATGAATATATGCAGCAAGCTCATGGAACCAAATATC :

AnFLC_A    : GATAATGTAAGTGTCGATACCCTCGTTCAGCTGGAGGAACACCTCGAGACTGCCCTCTCCGTAACTAGAG :
AnFLC_B    : GATAATATAAGTATCGATTCCCTCGTTAAGCTGGAGGAACACCTTGAAACTGCCCTCTCCGTAACTAGAG :

AnFLC_A    : CTAAGAAGGTATGTTGATTCCATATTGTTTTCTCTTTTCTTACTTTGTTTGTGGAGAACAACCGTGCAC- :
AnFLC_B    : CTAAGAAGGTATGTTGATTCCATATTGTCTTCTCTTTTCTTACTTTGTTTGTCGAAAACAGCGGTGCACT :

AnFLC_A    : ---------------------------------TTACATTTATATTGTTGCAGACAGAACTCATGTTGAA :
AnFLC_B    : TATAGTAATTTTGTTNNNNNNNNNNCGGTGCACTTATAGTAATTTTGTTGCAGAAAGAGCTAATGTTGAA :

AnFLC_A    : GCTTGTTGAGAGCCTTAAAGAAAAGGTTAGAAA-TTGCTTCCAAGTTTATAGCTCTTTATATATCTTCTC :
AnFLC_B    : GCTTGTTGAGAGCCTTAAAGAAAAGGTTAGAGATTTGCTTCCAAGTTTTTCGCACTTTATATATCTTCTC :
```

Figure S16 Statistical test on the SNP distribution in the alignment of (A) *AmFLC* and *PEP1* and (B-C) *FLC* and *AlFLC1*. The test measures whether the observed SNP number in a window is different from a random distribution (A and B more SNPs than expected, C fewer SNPs than expected); the left panel shows the number of SNPs per window, the right panel shows the –log10 of the associated p-values; the dashed line is the threshold above which statistical significance was reached; values were corrected for FDR.

A

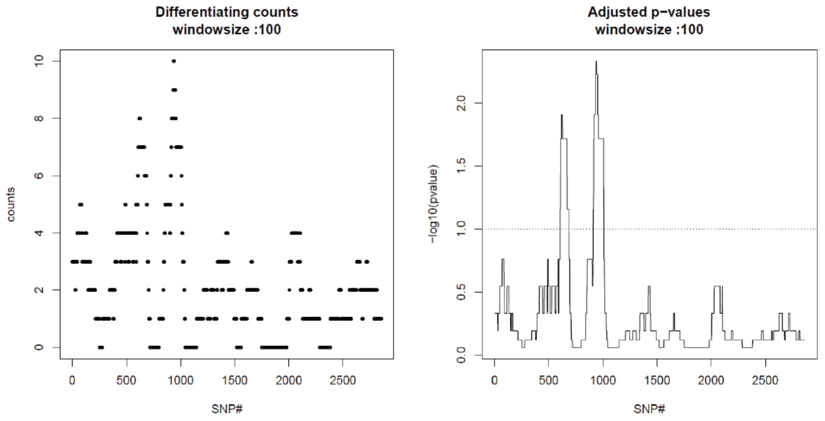

B

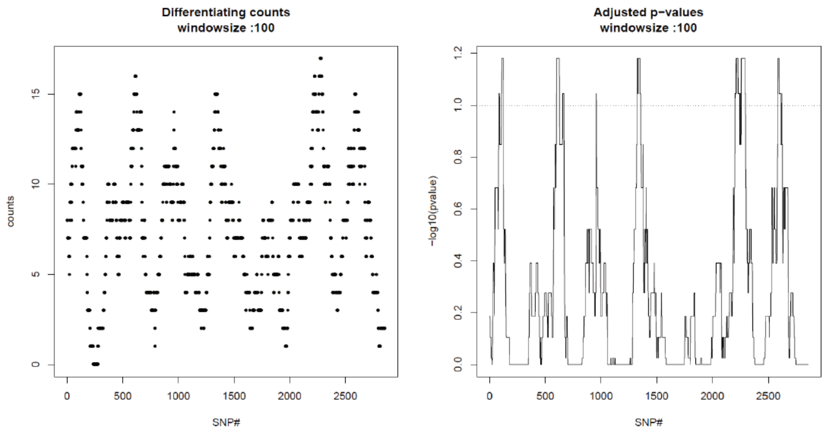

C

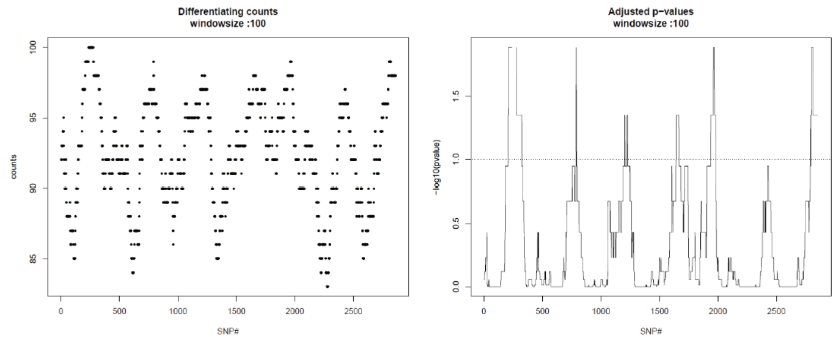

Figure S17 Statistical test on the SNP distribution in the alignment of (A) *AnFLC-A* and *PEP1* and (B) *AauFLC* and *AmFLC*. The testmeasures if the observed SNP number in a window is higher than a random distribution; the left panel shows the number of SNPs per window, the right panel shows the –log10 of the associated p-values; the dashed line is the threshold above which statistical significance was reached; values were corrected for FDR.

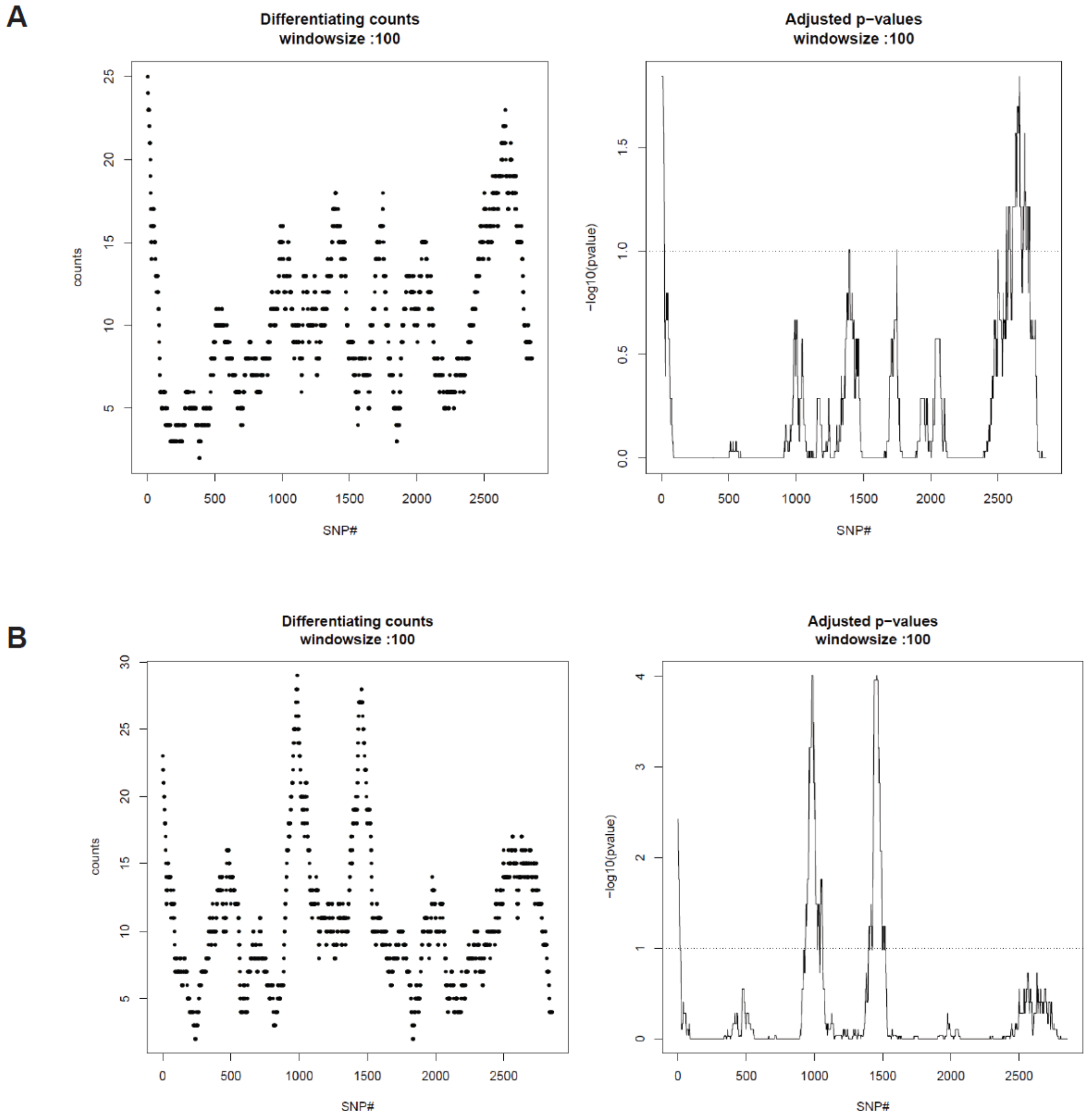

Figure S18 Statistical test on the SNP distribution in the alignment of (A) *AnFLC-A* and *PEP1* and (B) *AauFLC* and *AmFLC*. The test measures whether the observed SNP number in a window is lower than a random distribution; the left panel shows the number of SNPs per window, the right panel shows the –log10 of the associated p-values; the dashed line is the threshold above which statistical significance was reached; values were corrected for FDR.

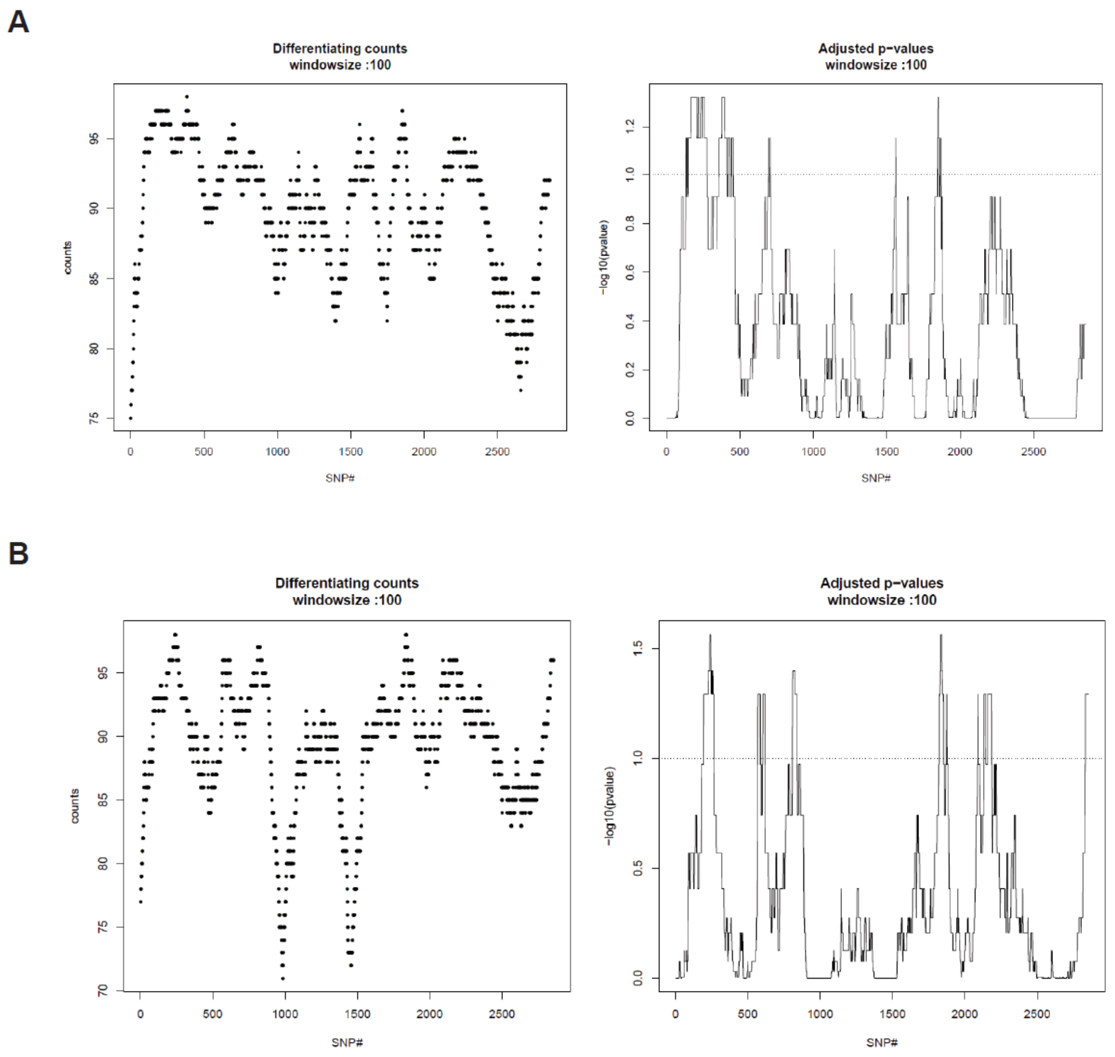

Figure S19 Sequence alignment of six Brassicaceae species in the segment showing more SNPs than expected by chance in the nucleation region from fig. 5A

```
PEP1     : GAACAATGTC--------GTGATGA-GC--T---------------------------------------
AmFLC    : GAACAATGTC--------GTGATGA-GC--T---------------------------------------
AlFLC1   : GAACAATGTCGTGAACTGGTGATGAAGC--T---------------------------------------
FLC      : GAACAATGTC--------GTGAAGAAGCTTT---------------------------------------
AnFLC-A  : GAAAAATGTC--------GTGATGA-GC--TAATGCCATATTTATGCATAATTTGTAATAGTATCATTAT
AauFLC   : GAACAATGTC--------GTGATGA-GC--T---------------------------------------

PEP1     : ----------------------------------------------------------------------
AmFLC    : ----------------------------------------------------------------------
AlFLC1   : ----------------------------------------------------------------------
FLC      : ----------------------------------------------------------------------
AnFLC-A  : CATTTGTTCAATTGTAATGAAACTTTTATAAAATATCATCTCCATCCACATGAAACTTTATGCAAAAGTT
AauFLC   : ----------------------------------------------------------------------

PEP1     : ----------------------------------------------------------------------
AmFLC    : ----------------------------------------------------------------------
AlFLC1   : ----------------------------------------------------------------------
FLC      : ----------------------------------------------------------------------
AnFLC-A  : TCGTAAAGATGATACTTTGCAAAAATTTCATTGAAAATTAATATTAAACAAATGCTAATGATACTCTGGC
AauFLC   : ----------------------------------------------------------------------

PEP1     : -------------------------------------AATGCTTCTTATACTTTCTTTCACTTCTCTCTC
AmFLC    : -------------------------------------AAAGCTTCT--TACTTTCTTTCACTTCTCTCTC
AlFLC1   : -------------------------------------AAAGCTTCT--TACTTTTGTTCATTTCTCTCTC
FLC      : -------------------------------------TTAGCTTCT--TACTTTTGTTCATTTCTCTCTC
AnFLC-A  : AAATCACGAATAACGATACTATTTCAAATTAATATTAAAAGCTTCT--TACTTTCTTTCACTTATCTCTC
AauFLC   : -------------------------------------AAAGCTTCT--TACTTTCTTTCACTTCTCTCTC

PEP1     : TCTCTTTTTCTTGATTTTCTTGACTTTCTTGACCTTTTTTT-GCTGCATGG-TTTTTTCTTTATATTGGT
AmFLC    : TCTC-TCTTTTTCTT------GATTTTCTTGAACTTTTTT--GCTGCATG--TTTTTTTATTTTATTGGT
AlFLC1   : TATA--------------------TTTCTTAATTTTTTTTT-CCTGCATGGATTTTATTATTTTCTTTGG
FLC      : --TA--------------------TTTCTTAAAAAAAAAAATTCTGCATGGATTTCATTATTT-CCTTGG
AnFLC-A  : TTTC-CCTC---------------TTTCTTGATTTTTTTT--CCTGCATG---TTTTTTATTTTATTGGT
AauFLC   : TCTCCTCTT---------------TTTCTTGATTTTTTT----CTGCATGGATTTTTTT-----ATTTGT

PEP1     : ATAAAATTTGCACGTCTTTCAAGATCTTTTTGATACGTTGTGATG--------------CATAATTGATG
AmFLC    : TAAAAATTTGCATGTGTTTCAAGATGTTTTTGATACGTTGTGATG--------------CATAATTGATG
AlFLC1   : AAATAATATGCATGTCCTTCACGATTTGTTTGATACGATCTGATGCGTGCTCCGTGCTCCGTGCTCGATG
FLC      : AAAAAAATTGCATGTCATTCACGATTTGTTTGATACGATCTGATG--------------CGTGCTCGATG
AnFLC-A  : TTAAAATTTGCATGTGTTTCAAGATCTTTTTGATACGTTGTGATG--------------CATAATTGATG
AauFLC   : TAAAAATTTGCATGTGTTTCAAGATCTCCTTGATACGTTGTGATG--------------CATACTTGATG

PEP1     : TTGTTTAGTGTAGTTTCA-A
AmFLC    : TTGTTTAGTGTAGTTTCA-A
AlFLC1   : TTGTTTAGTGAAGTTTCA-A
FLC      : TTGTTGAGTGAAGTTTCA-A
AnFLC-A  : TTGTTTAGTGTAGTTTCACA
AauFLC   : TTGTTTGGTGTAGCTTCA-A
```

Figure S20 Sequence alignment of six Brassicaceae species in the segment showing more SNPs than expected by chance upstream/in the region homologous to the VRE from fig. 5A

```
PEP1     : CCACGGATGTATTACTTGAAGATTATCCAAA-TTCATGAAGGTTTCTAAATGAATCT-----TAATTATC
AmFLC    : CCACGGATGTATTACTTGAAGATTATCCAAA-TTCATGAAAGTTTCTAAATGAATCT-----TAATTATC
AlFLC1   : TCGTGGACCTATTACTTGTTGATTATCTAAA-----T---------------------------------
FLC      : CTGTGGACCTATTACTTGGTGATTATCCAAA-----TTAGTGTTTTTAATTGATTCATA----TTTTTCA
AnFLC-A  : CCAC--ATGTAT---TTGAAGATTATCCA-ATTTAATGAAGGTTTCTATATGAATCT-----TAATTATC
AauFLC   : CCATGGATGTATTACTTGAAGATTATCCAAATTTAAGTAAGGTTTCC-AATGAATCTTATCGTAATTATA


PEP1     : ATATATCTATATATGTAGTACTTTTGAATTTT-AA-TTAGCTTGTAGCTTTGGTCACTAAAACCGACCAC
AmFLC    : ACG----TATATATATAGTA-------------AATTTAGCTTGTAGCTTTGGTCATTGAAATCGACAAC
AlFLC1   : ---------------------------------CCTCAACCTTGTAACTTTTGTTGTGAAAATCGACAAT
FLC      : TACACA-----------GTAGTT----------CCACAACC-TCAATCTTTTGTTGTGAAAATCGACAAT
AnFLC-A  : ATA----TATATA---TTTACTTTTGCATTTT-AATTTAATTTGTAGCTTTGGTCGTTGAAAACGACAAT
AauFLC   : GTA----TATAT----TGTACTTTTGAATTTT-ACACAACCTTGCAACTATTGTCGTGAAAATCGACAAT


PEP1     : CACACAACCTT-GTATCTA-------TTGCAGTGAAAATAGACAATTACGCAACCTTTTGTCT-TGCGTC
AmFLC    : CACACAACCTT-ATATATA-------TTGCAGTGAAAATCGACAATGACGCAACCTTTTGTCT-TGCGTC
AlFLC1   : CACACAACCTTTGTATCTTGTGTCTTTTGTCATAGAAATTGTCAATAACACAGCCTTGTTTCT-------
FLC      : CACACAACCTTTGTATCTTGTGTCTTTTGT----------------CACACAACCTTTGTATCTTGTGTC
AnFLC-A  : CACACAACCTT-GTATCTA-------TTGCCGTGAAATTCGGCAAGTACACAACCTGTTATCT-TGCGTC
AauFLC   : TACACAACCTT-GTATCTTGTGTCT-TTGTCATGGAAATGAACAAGAACACAACCTTTCTTTG-------


PEP1     : TTT-GTCATGGA-AATGGACAATAACACTGTACCTTTCTTTGGTGCCTCTTG
AmFLC    : TTT-GTCATGGA-AATGGACAATAACACTGTACCTTTCTTTGGTGCCTCTTG
AlFLC1   : ---------------------------------------TTGGTGCCTCTAG
FLC      : TTTTGTCATGGAAATTGTCATTCACACAGCCTTGTTTCTTTGGTGCCTCTAG
AnFLC-A  : TTT-GTCATGGA-AATGGACAATAACAC--TACCTTTCGTTGGTGCCTTTTG
AauFLC   : ------------------------------------------GTGCTTCTTG
```

Figure S21 Sequence alignment of six Brassicaceae species in the segment showing fewer SNPs than expected in a random distribution in the comparison of *AlFLC1* and *FLC* showing homology to (A) the end of the nucleation region and (B) COLDAIR from fig. 5A; the conserved GGATTTGT-motif is indicated a red square.

A

```
PEP1     : GTTCATCACTTGTTAATT--------GGCGTTAAAGAGT-TGTT---TACTAGATTGATTAATGAATAAC
AmFLC    : GTTCATCACTTGTTAATT--------GGCGTTAAAGAGT-TGTT---TACTAGATTGATTAATGAATAAC
AlFLC1   : GCTTTAGAGAT-TCCTTAA-------GTTTTTGAAGAGTT----------------GACTAAT-------
FLC      : CCTTTAGAGAT-TCCTTAA-------GTTTTTGAAGAGTTAATTATATATCACAA-GACTAAT-------
AnFLC-A  : GTTCATCAATTGTTAATTGGCTTTAAAGGATTAAAGAGT-TGTT---TAAGAGGTTAATTAAT-------
AauFLC   : GTTCATCACCTGTTAATT--------GTTTTTAAAGAGTGTGTT---TACTAGATTGATTAAT-------
```

```
PEP1     : GATTACTGTTTCTGTTTGTAGAGTAATTGAAAT-CATTGGATCTCTCGGATTTGTATGCAATGCAATACA
AmFLC    : GATTACTGTTTCTGTTTGTAGAGTAATTGAAAT-CATTGGATCTCTCGGATTTGTATGCAATGCA-----
AlFLC1   : GATTAATGTCTCTCTTTTCAGAGTGATTAAAATTCATTGGATCTCTCGGATTTGTATGCAATGCA-----
FLC      : GATTAATGTCT--CTTTTCAGAGTGATTAAAATTCATTGGATCTCTCGGATTTGT-----ATGCA-----
AnFLC-A  : GGTTACTGTTTCTGTTTGTAGAGTA----------ATTGGGTCTCTCGGATTTGTATGCAATGCA-----
AauFLC   : GATTACTGTTTCGGTTTGTAGAGTA----------ACTGGATCTCTCGGATTTGT--------------
```

```
PEP1     : ATGCACATC-GGGAGATCTATGGA
AmFLC    : ATGCACATC-GGGAGATCTATGGA
AlFLC1   : ATGCACCTACGGGAGATCTATAGG
FLC      : ATGCACTTACGGGAGATCTATAGA
AnFLC-A  : ATGCACATCGGGGAGATCTATAGA
AauFLC   : ATGCACATCGGGGAGATCTATAGA
```

B

```
PEP1     : ATTTGCTAGGATTTGTTCAGGCCGTA--TACTAAA--TGTT-GGGTCCAATCTTGGAATAGTCAAAACTC
AmFLC    : ATTTGCTAGGATTTGTTCAGGCCGTA--TACTAAA--TGTT-AGGTCCAATCTTGGAATAGTCAAAACTC
AlFLC1   : C----TTAGGATTTGTTCACCCCTTATATACTGAA--AGTT-AGGTCCAGCCTTGGAATAGTCGAGACAC
FLC      : CTTTGTTAGGATTTGTTCACCCCT-------------AGTT-AGGTCCAGCCTTGGAATTGTCGAGACAC
AnFLC-A  : ATTTGCTAGGATTTGTTCAGGCCT----TACTGAA--TGCT-AGGTTTACCTTGGAATAGTCAAAACTC
AauFLC   : ATTTGGTAGGATTTGTTCAGGCCTTA--TACTGAATGTGCC-AAGTCCAACCTTGGAATAGTCAAAACTC
```

```
PEP1     : CAGACTAGGACTTCTGATAT----------------------TACACATGAAAATCTCAATAGCTGTGTG
AmFLC    : CAGACTAGGACTTCTGATAT----------------------TACACATGAAAATCTCAATAGCTGTGTG
AlFLC1   : CTGACTAGAACTCCTGGTCT----------------------TAATTATG-------------ATTTGAT
FLC      : CTGACTAGAACTCCTGGTCT----------------------TAATTATG-------------ATTTAAT
AnFLC-A  : CAGACTAAGACTTCTGATAT----------------------TACACATGGAGATCTCAATAGATGTGTT
AauFLC   : CAGACTAGGACTTCTGGTATTACG------------------TACACATGGAAATCTCAATAGTTGTGTG
```

```
PEP1     : AGCAAAAATCAAGCCTTTTAGGAATTGGAA
AmFLC    : AGCAAAAATGAAGCCTTTTAGGAATTGGAA
AlFLC1   : AAAG---AAGAAGCCTTTTAGAACGTGGAA
FLC      : AAAG---AAGAAGCCTTTTAGAACGTGGAA
AnFLC-A  : AGCAAAA-ACAAGCCTTTTTGGAATTGGAA
AauFLC   : AGCAAAATAAAAGCCTTTTAGAAATTGGAA
```

Figure S22 Sequence alignment of six Brassicaceae species in the segment showing fewer SNPs than expected by chance within the region showing homology to the nucleation region in the annual couple (*AauFLC-AmFLC*) from fig. 5B

```
PEP1     : A-CATGAGAGATACATGAGTCAACTAAATTTGAGAAAGAACAATGTC--------GTGATGA-GC--T--
AmFLC    : A-CATGAGAGATACATGAGATAACTAAATTTGAGAAAGAACAATGTC--------GTGATGA-GC--T--
AlFLC1   : ACCATGAGAGATACATGAGATAACCTAATTTAAGGAAGAACAATGTCGTGAACTGGTGATGAAGC--T--
FLC      : ACCATGATAGATACATGAGATAACCAAATTTAAGGAAGAACAATGTC--------GTGAAGAAGCTTT--
AnFLC-A  : G-CATGAGAGATACATGAGATAACTAAATTTAAGGAAGAAAAATGTC--------GTGATGA-GC--TAA
AauFLC   : A-CATGAGAGATACATGAGATAACTAAATTTAAGGAAGAACAATGTC--------GTGATGA-GC--T--


PEP1     : ----------------------------------------------------------------------
AmFLC    : ----------------------------------------------------------------------
AlFLC1   : ----------------------------------------------------------------------
FLC      : ----------------------------------------------------------------------
AnFLC-A  : TGCCATATTTATGCATAATTTGTAATAGTATCATTATCATTTGTTCAATTGTAATGAAACTTTTATAAAA
AauFLC   : ----------------------------------------------------------------------


PEP1     : ----------------------------------------------------------------------
AmFLC    : ----------------------------------------------------------------------
AlFLC1   : ----------------------------------------------------------------------
FLC      : ----------------------------------------------------------------------
AnFLC-A  : TATCATCTCCATCCACATGAAACTTTATGCAAAAGTTTCGTAAAGATGATACTTTGCAAAAATTTCATTG
AauFLC   : ----------------------------------------------------------------------


PEP1     : ----------------------------------------------------------------------
AmFLC    : ----------------------------------------------------------------------
AlFLC1   : ----------------------------------------------------------------------
FLC      : ----------------------------------------------------------------------
AnFLC-A  : AAAATTAATATTAAACAAATGCTAATGATACTCTGGCAAATCACGAATAACGATACTATTTCAAATTAAT
AauFLC   : ----------------------------------------------------------------------


PEP1     : ----AATGCTTCTTATACTTTCTTTCACTTCTCTCTCTCTCTTTTTCTTGATTTTCTTGACTTTCTTGAC
AmFLC    : ----AAAGCTTCT--TACTTTCTTTCACTTCTCTCTCTC-TCTTTTTCTT------GATTTTCTTGAA
AlFLC1   : ----AAAGCTTCT--TACTTTTGTTCATTTCTCTCTCTATA--------------------TTTCTTAAT
FLC      : ----TTAGCTTCT--TACTTTTGTTCATTTCTCTCTC--TA--------------------TTTCTTAAA
AnFLC-A  : ATTAAAAGCTTCT--TACTTTCTTTCACTTATCTCTCTTTC-CCTC---------------TTTCTTGAT
AauFLC   : ----AAAGCTTCT--TACTTTCTTTCACTTCTCTCTCTCTCCTCTT---------------TTTCTTGAT


PEP1     : CTTTTTTT-GCTGCATG-GTTTTTTCTTTATATTGGTATAAAATTTGCACGTCTTTCAAGAT
AmFLC    : CTTTTTT--GCTGCATG-TTTTTTTATTTT-ATTGGTTAAAAATTTGCATGTGTTTCAAGAT
AlFLC1   : TTTTTTTT-CCTGCATGGATTTTATTATTTTCTTTGGAAATAATATGCATGTCCTTCACGAT
FLC      : AAAAAAAATTCTGCATGGATTTCATTATTT-CCTTGGAAAAAAATTGCATGTCATTCACGAT
AnFLC-A  : TTTTTTT--CCTGCATG---TTTTTTATTTTATTGGTTTAAAATTTGCATGTGTTTCAAGAT
AauFLC   : TTTTTT----CTGCATGGATTTTTTT-----ATTTGTTAAAAATTTGCATGTGTTTCAAGAT
```

Figure S23 Sequence alignment of six Brassicaceae species in the segment showing fewer SNPs than expected by chance within the region showing homology to the nucleation region in the perennial couple (*PEP1-AnFLC-A*) from fig. 5B

```
PEP1     : TAAAATTTGCACGTCTTTCAAGATCTTTTTGATACGTTGTGATG--------------CATAATTGATGT
AnFLC-A  : TAAAATTTGCATGTGTTTCAAGATCTTTTTGATACGTTGTGATG--------------CATAATTGATGT
AlFLC1   : AATAATATGCATGTCCTTCACGATTTGTTTGATACGATCTGATGCGTGCTCCGTGCTCCGTGCTCGATGT
AmFLC    : AAAAATTTGCATGTGTTTCAAGATGTTTTTGATACGTTGTGATG--------------CATAATTGATGT
AauFLC   : AAAAATTTGCATGTGTTTCAAGATCTCCTTGATACGTTGTGATG--------------CATACTTGATGT
FLC      : AAAAAATTGCATGTCATTCACGATTTGTTTGATACGATCTGATG--------------CGTGCTCGATGT


PEP1     : TGTTTAGTGTAGTTTCA-AGCTATCTTTGATTGTT----TGTTTGTTCATCA
AnFLC-A  : TGTTTAGTGTAGTTTCACACCCATCTTTGATTGTTTGTTTGTTTGTTCATCA
AlFLC1   : TGTTTAGTGAAGTTTCA-AGCCATCTTTGATTGTT----ACTTTGCTTTAGA
AmFLC    : TGTTTAGTGTAGTTTCA-AGCTATCTTTGATTGTT----TGTTTGTTCATCA
AauFLC   : TGTTTGGTGTAGCTTCA-AGCTATCTTTGAGTGTT----TGTTTGTTCATCA
FLC      : TGTTGAGTGAAGTTTCA-AGCCATCTTTGATTGTT----TCTTACCTTTAGA
```

LITERATURE CITED

Altschul SF, Madden TL, Schaffer AA, Zhang J, Zhang Z, Miller W, Lipman DJ. (1997) Gapped BLAST and PSI-BLAST: a new generation of protein database search programs. Nucleic Acids Research 25: 3389-3402.

Bolger AM, Lohse M, Usadel B. (2014) Trimmomatic: A flexible trimmer for Illumina Sequence Data. Bioinformatics.

Cantarel BL, Korf I, Robb SMC, Parra G, Ross E, Moore B, Holt C, Sánchez Alvarado A, Yandell M. (2008) MAKER: An easy-to-use annotation pipeline designed for emerging model organism genomes. Genome Research 18: 188-196.

Castaings L, Bergonzi S, Albani MC, Kemi U, Savolainen O, Coupland G. (2014). Evolutionary conservation of cold-induced antisense RNAs of *FLOWERING LOCUS C* in *Arabidopsis thaliana* perennial relatives. Nature Communications 5. doi: 10.1038/ncomms5457

Csorba T, Questa JI, Sun Q, Dean C. (2014) Antisense COOLAIR mediates the coordinated switching of chromatin states at FLC during vernalization. Proceedings of the National Academy of Sciences 111: 16160-16165.

Dobeš C, Mitchell-Olds T, Koch M. (2004) Extensive chloroplast haplotype variation indicates Pleistocene hybridization and radiation of North American *Arabis drummondii*, *A.* × *divaricarpa*, and *A. holboellii* (Brassicaceae). Molecular Ecology 13: 349-370.

Heo JB, Sung S. (2011) Vernalization-mediated epigenetic silencing by a long intronic noncoding RNA. Science 331: 76-79.

Koch M, Haubold B, Mitchell-Olds T. (2000) Comparative analysis of chalcone synthase and alcohol dehydrogenase loci in *Arabidopsis*, *Arabis* and related genera (Brassicaceae). Molecular Biology and Evolution 17: 1483–1498.

Li L, Stoeckert CJ, Roos DS. (2003) OrthoMCL: Identification of Ortholog Groups for Eukaryotic Genomes. Genome Research 13: 2178-2189.

Liu S, Liu Y, Yang X, Tong C, Edwards D, Parkin IAP, Zhao M, Ma J, Yu J, Huang S et al. (2014) The *Brassica oleracea* genome reveals the asymmetrical evolution of polyploid genomes. Nature Communications 5.

Marçais G, Kingsford C. (2011) A fast, lock-free approach for efficient parallel counting of occurrences of k-mers. Bioinformatics 27: 764-770.

Martin M. (2011) Cutadapt removes adapter sequences from high-throughput sequencing reads. EMBnet.journal 17: 10-12.

Milne I, Shaw P, Stephen G, Bayer M, Cardle L, Thomas WTB, Flavell AJ and Marshall D. 2010. Flapjack – graphical genotype visualization. Bioinformatics 26(24), 3133-3134.

Mummenhoff K, Franzke A, Koch MA. (1997) Molecular phylogenetics of *Thlaspi* s. l. (Brassicaceae) based on chloroplast DNA restriction site variation and sequences of the internal transcribed spacers of nuclear ribosomal DNA. Canadian Journal of Botany 75: 469–482.

Nei M, Kumar S (2000) Molecular Evolution and Phylogenetics. New York: Oxford University Press.

Nylander J, Wilgenbusch J, Warren D, Swofford D. (2007) AWTY (Are we there yet?): a system for graphical exploration of MCMC convergence in Bayesian phylogenetics. Bioinformatics 24: 581–583.

Parra G, Bradnam K, Korf I 2007. CEGMA: a pipeline to accurately annotate core genes in eukaryotic genomes. Bioinformatics 23: 1061-1067.

Posada D, Crandall K 1998. MODELTEST: testing the model of DNA substitution. Bioinformatics 14: 817-818.

Sheldon CC, Conn AB, Dennis ES, Peacock WJ 2002. Different Regulatory Regions Are Required for the Vernalization-Induced Repression of *FLOWERING LOCUS C* and for the Epigenetic Maintenance of Repression. Plant Cell 14: 2527-2537.

Shimodaira H, Hasegawa M (1999) Multiple comparisons of log-likelihoods with applications to phylogenetic inference. Molecular Biology and Evolution 16: 1114–1116.

Sung S, He Y, Eshoo T, Tamada Y, Johnson L, Nakahigashi K, Goto K, Jacobsen S, Amasino R. (2006) Epigenetic maintenance of the vernalized state in Arabidopsis thaliana requires *LIKE HETEROCHROMATIN PROTEIN 1*. Nature Genetics 38: 706-710.
Swiezewski S, Liu F, Magusin A, Dean C. (2009) Cold-induced silencing by long antisense transcripts of an Arabidopsis Polycomb target. Nature 462: 799-802.
Taberlet P, Gielly L, Pautou G, Bouvet J. (1991) Universal primers for amplification of three non-coding regions of chloroplast DNA. Plant Molecular Biology 17: 1105-1109.
Tamura K, Peterson D, Peterson N, Stecher G, Nei M, Kumar S. (2011) MEGA5: Molecular Evolutionary Genetics Analysis Using Maximum Likelihood, Evolutionary Distance, and Maximum Parsimony Methods. Molecular Biology and Evolution 28: 2731-2739.
Wang R, Farrona S, Vincent C, Joecker A, Schoof H, Turck F, Alonso-Blanco C, Coupland G, Albani MC. (2009) *PEP1* regulates perennial flowering in *Arabis alpina*. Nature 459: 423-427.
White T, Bruns T, Lee S, Taylor J. (1990) Amplification and direct sequencing of fungalribosomal RNA genes for phylogenetics. In: M. Innis DG, J. Sninsky and T. White, editor. PCR Protocols: a Guide to Methods and Applications. Orlando, Florida: Academic Press. p. 315-322.
Yang Z, Rannala B (1997) Bayesian Phylogenetic Monte Carlo Method Inference Using DNA Sequences: A Markov Chain. Molecular Biology and Evolution 14: 717-724.
Yang H, Howard M, Dean C. (2014) Antagonistic Roles for H3K36me3 and H3K27me3 in the Cold-Induced Epigenetic Switch at Arabidopsis *FLC*. Current Biology 24: 1793-1797.
